# Distinct Roles of Dopamine and Noradrenaline in Physical Fatigue

**DOI:** 10.1002/ejsc.70119

**Published:** 2026-01-27

**Authors:** Y. Laurisa Arenales Arauz, Ana Mali, Elke Lathouwers, Jelle Habay, Leonardo de Sousa Fortes, Romain Meeusen, Uros Marusic, Kevin De Pauw, Bart Roelands

**Affiliations:** ^1^ Human Physiology and Sports Physiotherapy Research Group Faculty of Physical Education and Physiotherapy Vrije Universiteit Brussel Ixelles Belgium; ^2^ Institute for Kinesiology Research Science and Research Centre Koper Koper Slovenia; ^3^ Research Foundation Flanders (FWO) Brussels Belgium; ^4^ LIFE Department Vital Signs and Performance Monitoring Research Unit Royal Military Academy Brussels Belgium; ^5^ Graduate Program in Sport Sciences Federal University of Minas Gerais Belo Horizonte Brazil; ^6^ BruBotics Vrije Universiteit Brussel Brussels Belgium; ^7^ Department of Sports, Recreation, Exercise and Sciences Faculty of Community and Health Sciences University of the Western Cape Cape Town South Africa; ^8^ Department of Health Sciences Alma Mater Europaea University Maribor Slovenia

**Keywords:** dopamine, exercise, fatigue, noradrenaline, performance

## Abstract

This triple‐blinded randomized crossover study investigated the roles of dopamine (DA) and noradrenaline (NA) in regulating exercise performance and fatigue using acute pharmacological manipulation. Eighteen healthy adults (9 males and 9 females; mean age = 23.4 ± 2.2 years) completed three experimental conditions. Participants ingested methylphenidate (MPH; 20 mg; primarily enhancing DA signaling), reboxetine (REB; 8 mg; selectively enhancing NA signaling), or placebo (10 mg lactose) prior to performing dynamic leg extensions until exhaustion. Behavioral, subjective, and physiological responses were assessed to evaluate drug‐specific effects using (generalized) linear mixed‐effects models. The fatiguing task effectively induced exhaustion in all conditions, evidenced by increases in self‐reported fatigue and exertion, heart rate, and blood lactate levels. MPH did not significantly improve the number of leg extension repetitions (+3.1%, *p* = 0.23) or perceived exertion ratings. Perceived performance and vigor increased, while sleepiness decreased across pretask and posttask assessments. Posttask temporal demand and overall task load were also reduced. In contrast, REB significantly reduced the number of leg extension repetitions (−13.2%, *p* < 0.001) without altering perceived exertion, mood, or performance perception. These findings show that DA and NA systems differently affect fatigue regulation. DA mainly influences cognitive and perceptual aspects, improving alertness and mood without significantly enhancing physical performance. In contrast, NA reduced physical performance without altering fatigue perception, indicating a dissociation between subjective fatigue and actual capacity. This study provides new evidence on how DA and NA shape both performance and perception during fatiguing leg‐extension exercise in males and females.

**Trial Registration:** G095422N and identifier NCT05880342

## Highlights

- Elevated brain dopamine levels boost alertness, vigor, and perceived performance without significantly enhancing leg extension performance in males and females.
- Elevated brain noradrenaline levels reduce leg extension performance while leaving perceptual responses unchanged in males and females.
- Dopaminergic and noradrenergic systems play distinct roles in separating cognitive‐perceptual and physical aspects of fatigue.

## 1 Introduction

Physical fatigue is a multifactorial phenomenon arising from both peripheral and central mechanisms that limit sustained voluntary muscle performance (Taylor et al. 2016). Peripheral fatigue originates within the muscle itself, primarily due to the accumulation of metabolites (e.g., inorganic phosphate, lactate, and H^+^) that impair excitation‐contraction coupling, reduce cross‐bridge cycling efficiency, and decrease force generation (Meeusen et al. 2021). Central fatigue stems from the central nervous system and is marked by a gradual decline in voluntary muscle drive, influenced by motor pathway excitability, peripheral inhibitory feedback, and neurochemical changes (Tornero‐Aguilera et al. 2022; Etemadi et al. 2023; Qi et al. 2022). These neurochemical factors are particularly important because they modulate motivation, arousal, and the perception of effort, directly influencing the capacity to sustain prolonged exercise (Meeusen et al. 2021). Despite their central role, the specific contributions of altered brain neurotransmission to exercise performance and fatigue perception remain incompletely understood.

Brain catecholamines, particularly dopamine (DA) and noradrenaline (NA), have been identified as potential modulators of physical fatigue and performance capacity (Meeusen et al. 2021; Meeusen and Roelands 2018). In normal ambient temperatures, methylphenidate (MPH; predominantly a DA reuptake inhibitor) significantly improved the time to exhaustion, with a coinciding increase in physiological strain (heart rate, oxygen consumption, and blood lactate concentration) but no change in rating of perceived exertion (RPE) (Swart et al. 2009). Yet, two other studies (Roelands, Hasegawa, et al. 2008; Klass et al. 2012) reported no improvements in performance following MPH administration. When participants had to perform in the heat (30°C), MPH significantly improved cycling time trial performance compared with placebo (Roelands, Hasegawa, et al. 2008). Despite increased power output and elevated core temperature, no changes in RPE and thermal stress were observed. Similar effects were observed after bupropion (a DA/NA reuptake inhibitor) administration in the heat (Watson et al. 2005; Roelands et al. 2012; Cordery et al. 2017). These results suggest that by sustaining central drive, DA can override regulatory mechanisms, allowing greater power outputs without a corresponding rise in RPE or thermal stress (Zheng and Hasegawa 2017).

Reboxetine (REB; a NA reuptake inhibitor) negatively impacted endurance performance in both normal (Klass et al. 2012; Roelands, Goekint, et al. 2008) and high (Roelands, Goekint, et al. 2008) ambient temperatures. During isometric maximal voluntary contractions (MVC), REB has been shown to enhance voluntary activation and increase corticospinal and spinal excitability (Klass et al. 2018). However, Klass et al. (2016) found that repeated submaximal isometric knee extensions (∼33% MVC) under REB led to reduced endurance, a faster decline in maximal voluntary torque and activation, and greater central and supraspinal fatigue. This indicates that, although REB can boost peak motor output, it may simultaneously impair the ability to sustain submaximal isometric efforts due to increased central fatigue. The effects of NA in dynamic contractions until exhaustion have not yet been investigated, which would more closely reflect real‐world fatigue scenarios.

Despite evidence that DA and NA influence physical fatigue and performance, prior studies typically examined these neuromodulators in isolation, focused on male participants (Swart et al. 2009; Roelands, Hasegawa, et al. 2008; Klass et al. 2012; Watson et al. 2005; Roelands et al. 2012, Roelands, Goekint, et al. 2008; Klass et al. 2018, 2016), and relied on limited outcome measures. This leaves gaps in understanding how DA and NA may differentially affect performance, perception, and physiology across individuals and sexes. The present study addresses this gap by simultaneously investigating both catecholamines within the same crossover protocol, including male and female participants, and integrating behavioral, physiological, and subjective measures. Applying mixed‐effects statistical models further allows us to account for interindividual variability (Lyauk et al. 2016) and potential covariates, providing a more nuanced understanding of how central catecholamines influence both fatigue perception and physical performance.

This study aimed to investigate the effects of MPH and REB versus placebo on exercise performance, fatigue, and perceptual responses during and after dynamic leg extensions to exhaustion in male and female participants. Primary outcomes included maximal repetitions, self‐reported physical fatigue, and RPE. Secondary measures comprised muscle contractile properties, blood lactate, heart rate, and subjective ratings of motivation, mood, task load, and sleepiness. We hypothesized that NA reuptake inhibition (REB) would impair, and DA reuptake inhibition (MPH) would enhance performance. No condition differences in self‐reported physical fatigue or RPE were expected.

## 2 Materials and Methods

### 2.1 Ethics and Study Methodology

#### 2.1.1 Ethics

The study was approved by the Medical Ethics Committee (protocol number: 2022‐002836‐30) in accordance with the Declaration of Helsinki and received additional approval from the Federal Agency for Medicines and Health Products (1311860). This study was part of a large project that was registered on clinicaltrials.gov (NCT05880342) following the SPIRIT guidelines (Chan et al. 2013) and the statistical assessment checklist (Mansournia et al. 2021). The authors declared no potential conflicts of interest, which was disclosed to all participants.

#### 2.1.2 Study Design and Setting

A randomized, counterbalanced, and within‐subject crossover design was used to examine the roles of DA and NA in physical fatigue. The trials took place in the morning (7:00 a.m.–12:00 p.m.) in a sound‐insulated laboratory at the Human Physiology and Sports Physiotherapy research group (MFYS, Vrije Universiteit Brussel). Participants completed one familiarization trial and three experimental trials, each separated by 1–2 weeks. Within each experimental trial, either MPH, REB, or placebo was administered. All procedures were overseen by trained staff and certified medical professionals (GCP certified) (CH Harmonised Guideline 2016). Two researchers were present during each session to ensure smooth execution.

#### 2.1.3 Drug Randomization and Blinding

All parties involved in the study remained blinded (triple‐blind design). A Latin square technique was used to ensure a balanced condition order. The hospital pharmacy prepared participant kits with identical tablets and assigned coded conditions. MPH (Ritalin) was administered at 20 mg (2 × 10 mg) to inhibit DA reuptake; REB (Edronax) at 8 mg (2 × 4 mg) to inhibit NA reuptake; and placebo as 2 lactose tablets (2 × 5 mg). Researchers were unblinded after data analysis was completed.

### 2.2 Participants

Participants were students, aged 18–35 who engaged in physical activity at least once weekly. We excluded individuals with a high level of competitive or elite training to avoid confounding effects of advanced athletic conditioning. All were nonsmokers and showed no signs of general fatigue (Mental Fatigue Index > 57) (Smets et al. 1995; Manoli et al. 2020), depression (BDI‐II > 16) (Wang and Gorenstein 2013), or burnout (BAT ≥ 2.59) (Schaufeli et al. 2020). Exclusion criteria included pregnancy, allergies to study substances (e.g., lactose, gluten), chronic illness, psychiatric history, recent injury (within 6 months), or involvement in other studies or treatments. Participants were asked to avoid strenuous exercise, alcohol, caffeine, and medications for 24 h before each trial. Participants consumed a standardized meal the evening before, had slept at least 7 hours, and arrived in a fasted state to the trial. A pretest checklist confirmed compliance with all instructions.

### 2.3 Experimental Protocol

#### 2.3.1 Familiarization Trial

Prior to participating in the study, all participants underwent a thorough sports medical evaluation conducted by a licensed medical specialist. This assessment was designed to detect medical conditions or risk factors that might affect eligibility or require medical clearance before participation. The evaluation comprised a medical and sports history, current and past medications, anthropometrics (using Tanita TBF‐300), a general clinical examination, and a resting ECG. Participants provided informed consent, received an explanation of the procedures, and followed a sequence mirroring the experimental trial to familiarize themselves with the tasks. Familiarization to the protocol involved a warm‐up of three sets of 10 leg extensions (5 kg, 10 kg, and 15 kg), followed by an estimation of one‐repetition maximum (1RM) to determine individual loading. Participants selected a weight corresponding to their perceived 8–10 RM. If more than 10 repetitions were completed, the load was increased incrementally with 3–5 min of rest between attempts until failure occurred. The final load was used to estimate 1RM using the Lombardi formula: load (kg) × repetitions^0.10^ (Lombardi 1989). Forty percent of the estimated 1RM was used as the individualized load for experimental trials.

#### 2.3.2 Experimental Trial

**FIGURE 1:**
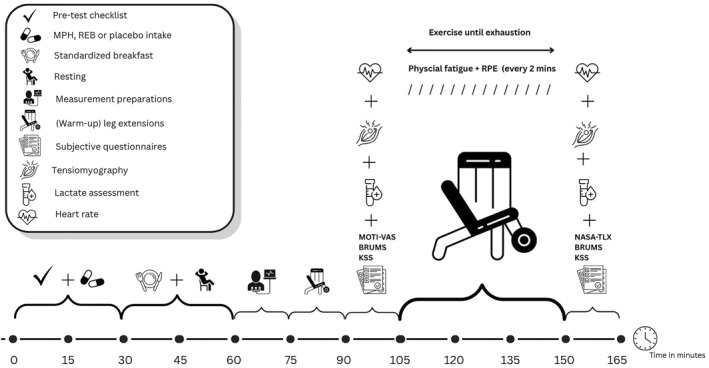
Experimental protocol. BRUMS: Brunel Mood Scale; KSS: Karolinska Sleepiness Scale; MOTI‐VAS: Visual Analog Scale (0–100) for motivation levels; MPH: 20 mg Methylpendidate; NASA‐TLX: National Aeronautics and Space Administration Task Load Index; REB: 8 mg Reboxetine; RPE: Rating of perceived exertion using the OMNI‐RPE scale specified for resistance exercise.

Figure 1 outlines the experimental trial. Upon arrival, participants completed a pretest checklist and ingested their assigned drug (placebo, MPH, or REB). Thirty minutes after ingestion, they consumed a standardized breakfast (620 kcal; 117 g of carbohydrates, 3.05 g of fat, and 26 g of protein) to ensure consistent nutritional status. Participants were then seated in a leg extension device, and their skin was prepared for placement of a 12‐lead ECG. After this, Tensiomyography (TMG) electrodes were positioned on the right vastus medialis according to the SENIAM guidelines (Hermens et al. 2000). After all participants were equipped with the measurement devices, the measurements began approximately 1–1.5 hours after drug ingestion. Participants completed a warm‐up of three sets of 10 leg extensions at 20% of their estimated 1RM. After the warm‐up, baseline measures were assessed, including muscle contractile properties, blood lactate levels, heart rate, and self‐reported motivation, mood state, and sleepiness. For the contractile properties, the TMG sensor was aligned perpendicularly over the marked site, and electrical stimulation intensity was gradually increased until maximal muscle displacement (Dm) plateaued as a baseline measurement (Šimunic et al. 2018; Šimunič 2012). Participants then performed leg extensions to failure, guided by visual cues on a screen. RPE and physical fatigue were assessed at baseline, every 2 minutes, and at task failure. The number of leg extension repetitions was counted and participant's posture was continuously monitored. Immediately after the physically fatiguing task, muscle contractility, heart rate, lactate, and self‐reported state of mood, sleepiness, and task load were assessed.

### 2.4 Devices and Outcomes

#### 2.4.1 Performance Measures

During the leg extension task, participants used a commercially available leg extension device (Absolute Line, Toorx Professional). Each repetition consisted of an active knee extension (concentric quadriceps contraction) followed by a controlled flexion back to the starting position (eccentric return), lasting 1.5 s per phase. Each repetition (3 s total) was guided by visual cues programmed in E‐Prime 3.0 software. Intervals between repetitions varied randomly between 3 and 5 s to prevent anticipation of the next contraction. Failure was defined as the point at which participants could no longer complete a full extension with proper form. Physical performance was recorded as the total number of leg extensions completed until failure using a manual counter.

#### 2.4.2 Subjective Measures

Assessments based on self‐reported questionnaires or scales were collected and managed using the REDCap system (Vanderbilt University Medical Center, Nashville, TN, USA) on a tablet, which is a secure web‐based electronic data capture tool hosted at UZ Brussel (Harris et al. 2019, 2009). The measures included assessments of motivation prior to the task, physical fatigue and RPE during the task, mood and sleepiness both pretask and posttask, and task load following the task. Motivation was assessed using a visual analog scale (VAS) (Wewers and Lowe 1990), where participants responded to the prompt, “How motivated do you feel for the upcoming task?”, by marking a 10 cm line anchored by “not at all” and “extremely motivated.” Physical fatigue was assessed verbally using the question, “How physically fatigued do you feel on a scale of 0–100?” RPE was measured with the OMNI‐RPE scale for resistance exercise (0–10 scale) (Robertson et al. 2003), and it was printed and mounted on the wall in front of participants, with verbal prompts provided as needed. Task load was evaluated using the NASA Task Load Index (NASA‐TLX) (Hernandez et al. 2022), comprising six subscales: mental demand, physical demand, temporal demand, performance, effort, and frustration. Mood was assessed using the Brunel Mood Scale (BRUMS) (Parsons‐Smith et al. 2017), which included subscales for tension, depression, anger, vigor, fatigue, and confusion. Sleepiness was measured with the Karolinska Sleepiness Scale (KSS) (Kaida et al. 2006).

#### 2.4.3 Physiological Measures

Physiological measurements were collected before and immediately after the leg extension task to assess the effects of the fatigue protocol and pharmacological condition. Capillary blood samples were collected from the fingertip to measure blood lactate concentrations (mmol/L), which were enzymatically analyzed using the Biosen 5030 (EKF‐Diagnostic GmbH, Germany). Heart rate was monitored using a 12‐lead ECG device (Cardioflex PC‐ECG), positioned on the participant's chest and connected to CardioFlex software via USB. Pretask and posttask heart rates were recorded to evaluate the success of the fatigue induction.

Muscle contractile properties of the right vastus medialis were assessed using TMG (TMG‐S1, TMG‐BMC Ltd., Ljubljana, Slovenia). The muscle belly was located and marked on the medial thigh proximal to the patella according to SENIAM guidelines. The TMG displacement sensor was positioned over the marked point, and two self‐adhesive electrodes (5 × 5 cm) were placed ∼1 cm next to the sensor. The knee of the measured leg was set at 30° flexion to ensure a relaxed muscle state. For the baseline measurement, the electrical stimulation intensity was gradually increased until maximal muscle displacement (Dm) plateaued. Within ten seconds after exhaustion, the right leg was positioned, the TMG sensor was placed, and two consecutive stimulations were delivered with 10–15 s in between them. The mean of these two responses was used for analysis, and the inter‐stimulus interval was chosen to minimize potentiation or cumulative effects (Šimunic et al. 2018; Šimunič 2012). From each twitch response, contraction time (Tc, the time from 10% to 90% of Dm) and Dm (mm) were extracted.

### 2.5 Statistical Analyses

#### 2.5.1 Data Preparation

Data from REDCap, TMG, and Cardioflex were exported and structured in long format to enable the analysis of both single and repeated measures. Selected variables underwent preprocessing before statistical analysis. NASA‐TLX scores were computed by averaging the subscales (mental demand, physical demand, temporal demand, performance, effort, and frustration). BRUMS subscale scores (tension, depression, anger, vigor, fatigue, and confusion) were derived by summing the relevant item scores.

Physical fatigue and RPE were assessed at varying time points across participants (every 2 minutes). To standardize these data, each assessment was expressed as a percentage of task failure (0% = baseline and 100% = final effort) and intermediate points linearly scaled. Linear interpolation was then used to estimate values at 25%, 50%, and 75% of task failure for each participant. For muscle contractile properties (Dm and Tc), the two measurements per timepoint were averaged to obtain a single value.

#### 2.5.2 Data Analyses

All statistical analyses were performed in RStudio version 4.2.2 (R Core Team) using the following packages: lme4 (Douglas and Bolker 2015), lmerTest (Kuznetsova et al. 2017), emmeans (Lenth 2024), DHARMa (Hartig 2024), glmmTMB (Mollie et al. 2017), and ARTool (Kay and Higgins 2025). The complete dataset and R code are available in Supporting Information S1 and S2.

Data were initially inspected using boxplots and histograms to identify outliers and assess the distribution. Outliers were evaluated and excluded only if they were deemed implausible, defined as values outside the physiologically or instrumentally possible range or inconsistent with the study protocol. Descriptive statistics (means and standard deviations or median and interquartile range) were calculated for each variable, grouped by drug and time point. Depending on the outcome distribution and type, appropriate models were applied (see Table 1). Fixed factors included drug and time, with subject entered as a random factor to account for individual variability. For leg extension performance, trial number and sex were added as fixed effects to control for learning and sex differences.

**TABLE 1:** Model specifications per research question.

Research questions	Dependent variables	Model specifications		
		Model	Fixed factors	Random factor
Primary				
Does drug condition influence the number of repetitions performed?	Number of repetitions	GLMM^a^	Drug (3x) + Trial number (3x) + sex (2x)	Subjects
Does subjective fatigue and exertion increase over the course of the physical fatigue task and does drug condition modulate this effect?	Subjective physical fatigue	ART model	Drug (3x) + Time (5x)	Subjects
	Rate of perceived exertion (RPE)	ART model	Drug (3x) + Time (5x)	Subjects
Secondary				
Do subjective factors change after the fatigue‐inducing task and does drug condition modulate this effect?	Motivation (MOTI‐VAS)	LMM^b^	Drug (3x)	Subjects
	Averaged workload (NASA‐tlx)	ORM	Drug (3x)	Subjects
	– All subscales	ORM	Drug (3x)	Subjects
	Sleepiness (KSS)	ORM	Drug (3x)	Subjects
	Mood state (BRUMS)			
	– Confusion	GLMM^c^	Drug (3x) × Time (2x)	Subjects
	– Tension	GLMM^c^	Drug (3x) × Time (2x)	Subjects
	– Anger	GLMM^c^	Drug (3x) × Time (2x)	Subjects
	– Fatigue	LMM	Drug (3x) × Time (2x)	Subjects
	– Depression	GLMM^c^	Drug (3x) × Time (2x)	Subjects
	– Vigor	LMM	Drug (3x) × Time (2x)	Subjects
Does physiological or metabolic responses change after the fatigue‐inducing task and does drug condition modulate this effect?	Heart rate	LMM^b^	Drug (3x) × Time (2x)	Subjects
	Lactate	LMM^b^	Drug (3x) × Time (2x)	Subjects
	Contraction time (Ct)	LMM	Drug (3x) × Time (2x)	Subjects
	Muscle displacement (Dm)	LMM	Drug (3x) × Time (2x)	Subjects

Abbreviations: GLMM: Generalized linear mixed model; LMM: Linear mixed model; ORM: Ordinal regression model; MOTI‐VAS: Visual analog scale of 0–100 of motivation; KSS: Karolinska Sleepiness Scale; BRUMS: Brunel Mood scale; NASA‐TLX: National Aeronautics and Space administration Task Load Index.

^a^ Poisson distribution.

^b^ log transformed.

^c^ with a negative binomial distribution.

Model assumptions were evaluated using the DHARMa package (Hartig 2024), which produced scaled simulation‐based residuals for (generalized) linear mixed models. Diagnostic plots confirmed that residuals followed the expected uniform distribution, with Q–Q plots and boxplots aligned along the 1:1 diagonal and median near 0.5, indicating good model fit. Homoscedasticity and absence of overdispersion were supported by residuals‐versus‐fitted plots and a non‐significant dispersion test. Zero‐inflation testing confirmed appropriate model specification. When assumptions were violated, data transformations (logarithmic, square root, and inverse) were applied and models refitted. If violations persisted, alternative models were used.

Pairwise contrasts were computed using estimated marginal means, comparing adjacent percentiles of RPE and physical fatigue. A Bonferroni‐corrected significance level of *α* = 0.0167 was applied for the three primary outcomes (repetitions, RPE, and physical fatigue), whereas secondary outcomes were evaluated at *α* < 0.05.

## 3 Results

Table 2 presents a detailed overview of all descriptives and model outputs.

**TABLE 2:** Model specifications per research question.

Outcomes	Descriptives (Mean ± std/median [IQR])			Model output					
	PLA	MPH	REB	Drug effects		Time effects		Interaction effects	
				(Estimate, [95% CI]/F (df1, df2))	*p*	(Estimate, [95% CI]/F (df1, df2))	*p*	(Estimate, [95% CI]/F (df1, df2))	*p*
Performance									
Number of repetitions (count data)	173.5 ± 110.1	175.9 ± 91.2	149.4 ± 87.7	Int (PLA): 135.9, [101.02, 182.8]				/	
				MPH: RR = 1.03, [1.04, 1.15]	0.234	**Visit 2: RR = 1.10, [1.04, 1.16]**	**< 0.001** **		
				**REB: RR = 0.86, [0.82, 0.91]**	**< 0.001** **	**Visit 3: RR = 1.13, [1.04, 1.15]**	**< 0.001** **		
Subjective									
Motivation before the physical task (0–100)	69.9 ± 19.9	72.6 ± 13.4	68.8 ± 18.3	Int (PLA): 66.40, [58.0, 76.0]		/		/	
				MPH: *β*inv = 1.09, [0.97, 1.23]	0.148				
				REB: *β*inv = 0.99 [0.8, 1.10]	0.926				
Perceived workload of the physical task (0–20)									
Mental	7.8 ± 5.5	7.2 ± 6.1	6 ± 5.2	MPH: OR = 0.66, [0.17, 2.56]	0.545	/		/	
				REB: OR = 0.41, [0.11, 1.57]	0.192				
Physical	17.6 ± 1.8	18 ± 1.8	17.9 ± 1.5	MPH: OR = 1.72, [0.51, 5.79]	0.384	/		/	
				REB: OR = 1.58, [0.47, 5.25]	0.457				
Temporal	10.8 ± 4.4	10.1 ± 5.6	10 ± 5.1	**MPH: OR = 0.72, [0.71, 0.72]**	**< 0.001** **	/		/	
				**REB: OR = 0.88, [0.87, 0.88]**	**< 0.001** **				
Effort	17.4 ± 1.8	17.3 ± 2.5	17 ± 2.0	MPH: OR = 1.03, [0.30, 3.5]	0.966	/		/	
				REB: OR = 0.59, [0.18, 1.91]	0.375				
Performance	8.1 ± 5.5	5.2 ± 4.5	7.6 ± 4.8	**MPH: OR = 0.17, [0.05, 0.64]**	**0.008** *	/		/	
				REB: OR = 0.77, [0.23, 2.55]	0.668				
Frustration	8.3 ± 5.1	4.8 ± 5.0	6.3 ± 5.1	MPH: OR = 0.26, [0.07, 1.04]	0.056	/		/	
				REB: OR = 0.28, [0.07, 1.10]	0.068				
Averaged	11.7 ± 1.9	10.45 ± 2.3	10.8 ± 1.7	Int (PLA):12.50, [11.6, 13.6]		/		/	
				**MPH: *β*inv = 0.90, [0.84, 0.96]**	**0.003** *				
				**REB: *β*inv = 0.93, [0.87, 1.00]**	**0.049** *				
Subjective physical fatigue (0–100)	0%: 12.7 ± 14.2	0%: 10.3 ± 15.1	0%: 9.4 ± 10.8	F (2, 255) = 0.81	0.445	**F(4, 255) = 275.8**	**< 0.001** ^a^ ^,^ **	Drug x T: F (8, 255) = 0.53	0.828
	25%: 48.6 ± 19.9	25%: 48.6 ± 19.3	25%: 44.3 ± 18.6						
	50%: 74 ± 17.1	50%: 77.1 ± 11.7	50%: 74.8 ± 13.5						
	75%: 86.6 ± 8.7	75%: 91 ± 10.2	75%: 89.2 ± 9.6						
	100%: 95.8 ± 5.7	100%: 95.2 ± 8.4	100%: 96.2 ± 6.0						
Rating of perceived exertion (0–10)	0%: 4.1 ± 1.9	0%: 4.1 ± 1.6	0%: 3.9 ± 1.7	F (2, 255) = 0.65	0.521	**F(4, 255) = 179.12**	**< 0.001** **^a^** ^,^ **	Drug × T: F (8, 255) = 0.40	0.921
	25%: 5.7 ± 2.1	25%: 5.9 ± 1.6	25%: 5.3 ± 1.8						
	50%: 7.6 ± 1.6	50%: 7.8 ± 1.2	50%: 7.7 ± 1.5						
	75%: 9.0 ± 0.9	75%: 9.0 ± 0.8	75%: 9.0 ± 1.1						
	100%: 9.6 ± 0.6	100%: 9.7 ± 0.65	100%: 9.6 ± 0.7						
Sleepiness (0–10)	PRE 5.3 ± 1.7	PRE 3.9 ± 1.3	PRE 5.4 ± 1.9	**MPH: OR = 0.17, [0.05, 0.58]**	**0.004** *	OR = 0.46, [0.14, 1.53]	0.206	MPH × T: OR = 1.51, [0.28, 8.13],	0.629
	POST 4.7 ± 1.9	POST 3.7 ± 1.3	POST 3.8 ± 0.9	REB: OR = 1.16, [0.34, 3.98]	0.817			REB × T: OR = 0.27, [0.05, 1.49]	0.133
Mood state (0–16)^b^									
Confusion	PRE 0 [0–2] POST 0 [0–2]	PRE 0 [0–1.0] POST 0 [0−0.75]	PRE 0.5 [0–3.0] POST 0 [0–1]	Int (PLA): 0.70, [0.06, 0.99]					
				MPH: IRR = 0.90, [0.48, 1.69]	0.752	IRR = 0.86, [0.45, 1.61]	0.631	MPH × T: IRR = 1.11, [0.45, 2.74]	0.828
				REB: IRR = 1.28, [0.72, 2.29]	0.400			REB × T: IRR = 0.95, [0.41, 2.24]	0.912
Tension	PRE 0 [0–1.0] POST 0 [0−0.75]	PRE 0 [0–1.0] POST 0 [0–0]	PRE 0.5 [0–3.0] POST 0 [0–1.0]	Int (PLA): 0.17, [0.06, 0.52]					
				MPH: IRR = 2.0, [0.97, 4.12]	0.060	IRR = 1.18, [0.53, 2.64]	0.683	MPH × T: IRR = 0.42, [0.14, 1.25]	0.119
				**REB: IRR = 2.45, [1.22, 4.95]**	**0.012** *			REB × T: IRR = 0.53, [0.19, 1.46]	0.182
Anger	PRE 0 [0–1.75] POST 0 [0–0]	PRE 0 [0–0] POST 0 [0–0]	PRE 0 [0−0.75] POST 0 [0–0]	Int (PLA): 0.11, [0.02, 0.74]					
				MPH: IRR = 0.72, [0.32, 1.64]	0.435	*β* = 0.62, [0.27, 1.40]	0.247	MPH × T: IRR = 1.11, [0.31, 3.92]	0.874
				REB: IRR = 0.87, [0.42, 1.82]	0.711			REB × T: IRR = 1.15, [0.36, 3.7]	0.815
Fatigue	PRE 4.5 [2.25–6.75] POST 6 [4.25–9.5]	PRE 2 [0.5–3.0] POST 6 [4.25–7]	PRE 5.5 [2.25–7.25] POST 6.5 [5.0–8.0]	Int (PLA): 4.44, [3.07, 5.82]					
				**MPH: *β* = 0.89, [–3.16, −0.17]**	**0.032** *	***β* = 2.17, [0.67, 3.66]**	**0.006** *	MPH × T: *β* = 0.94, [−1.17, 3.06]	0.384
				REB: *β* = 0.84, [−0.6, 2.8]	0.247			REB × T: *β* = −1.06, [−3.17, 1.06]	0.330
Depression	PRE 1 [0–1.75] POST 0 [0−0.75]	PRE 0.5 [0–1] POST 0 [0–1]	PRE 1 [0–1.0] POST 0 [0–1.75]	Int (PLA): 0.80, [0.40, 1.59]					
				MPH: IRR = 0.77, [0.4, 1.45]	0.425	IRR = 0.61, [0.32, 1.18]	0.191	MPH × T: IRR = 0.37, [0.37, 2.8]	0.976
				REB: IRR = 0.82, [0.44, 1.53]	0.536			REB × T: IRR = 0.51, [0.51, 3.4]	0.577
Vigor	PRE 7 [3.25–8] POST 8 [4.25–10.8]	PRE 8 [5.25–9.8] POST 8 [5.25–11]	PRE 5 [3–8] POST 8 [5–10]	Int (PLA): 5.83, [4.25, 7.41]					
				**MPH: *β* = 1.72, [0.19, 3.26]**	**0.031** *	**β = 1.61, [0.07, 3.15]**	**0.043** *	MPH × T: *β* = −1.50,[−3.67, 0.67]	0.180
				REB: *β* = −0.56, [−2.09, 0.98]	0.480			REB × T: *β* = 0.56,[−1.62, 2.73]	0.618
Physiological									
Heart rate (bpm)	PRE 91.7 ± 13.3 POST 143 ± 23.9	PRE 97.9 ± 14 POST 157.3 ± 21.6	PRE 105.9 ± 15.18 POST 155.6 ± 19.1	Int (PLA): 90.81, [84.7, 97.3]					
				**MPH: *β*inv = 1.07, [1.0, 1.14]**	**0.045** *	***β*inv = 1.55, [1.46, 1.65]**	**< 0.001** **	MPH × T: *β*inv = 1.03, [0.95, 1.13]	
				**REB: *β*inv = 1.15, [1.08, 1.23]**	**< 0.001** **			REB × T: *β*inv = 0.96, [0.87, 1.04]	0.251
Lactate (mmol/L)	PRE 2.8 ± 0.98 POST 4.97 ± 2.45	PRE 2.9 ± 1.03 POST 5.2 ± 1.9	PRE 3.27 ± 2.0 POST 6.2 ± 2.5	Int (PLA): 2.64, [2.15, 3.14]					
				MPH: *β*inv = 1.06, [0.85, 1.32]	0.704	***β*inv = 1.71, [1.37, 2.14]**	**< 0.001** **	MPH × T: *β*inv = 1.01, [0.73, 1.40]	0.948
				REB: *β*inv = 1.09, [0.88, 1.37]	0.505			REB × T: *β*inv = 1.16, [0.83, 1.61]	0.387
Contraction velocity (ms)	PRE 19.5 ± 4.6 POST 17.6 ± 3.9	PRE 19.2 ± 3.2 POST 18.5 ± 2.7	PRE 19.2 ± 2.4 POST 18.5 ± 2.4	Int (PLA): 19.5, [18.0, 21.0]					
				MPH: *β* = −0.32, [−1.70, 1.10]	0.656	***β* = −1.89, [−3.3,−0.48]**	**0.011** *	MPH × T: *β* = 1.21, [−0.8, 3.2]	0.240
				REB: *β* = −0.29, [−1.70, 1.10]	0.690			REB × T: *β* = 1.15, [−0.9, 3.2]	0.263
Muscle displacement (cm)	PRE 3.8 ± 2.20 POST 3.9 ± 2.35	PRE 3.95 ± 2.14 POST 3.52 ± 2.0	PRE 4.2 ± 2.2 POST 3.48 ± 1.9	Int (PLA): 3.46, [2.52, 4.55]					
				MPH: *β* = 0.15, [−0.70, 1.0]	0.734	*β* = 0.12, [−0.7, 1.0]	0.775	MPH × T: *β* = −0.6, [−1.8, 0.6]	0.364
				REB: *β* = 0.36, [−0.50, 1.20]	0.410			REB × T: *β* = −0.8, [–2, 0.4]	0.189

*Note*: Bold values indicate significant results.

Abbreviations: IRR: Incidence Rate Ratio; OR: Odds Ratio; RR: Rate Ratio; *β*: raw estimates; *β*inv: represents back‐transformed estimates (e.g., squared or exponentiated) to return estimates to the original scale of the outcome variable.

^a^ Pairwise comparisons with Bonferroni correction showed no significant differences between time points (*all p* > 0.001).

^b^ Mood scale data are summarized as median and interquartile range due to their non‐normal distribution.

^*^ *p* > 0.05.

^**^ *p* > 0.001.

### 3.1 Participants

An a priori power analysis (G*Power, v3.1) (Faul et al. 2007) indicated that 18 participants were required to achieve 90% power (*α* = 0.05) based on an expected effect size of ηp^2^ = 0.258 (Jacquet et al. 2021). We recruited 19 participants, of whom eighteen (9 males and 9 females; age 23.4 ± 2.2 years; height: 170 ± 9.4 cm; body mass: 66.1 ± 9.9 kg; BMI 22.9 ± 2.6 kg/m^2^; fat percentage 20.8 ± 8.5%) were included in the final analysis. One participant was excluded due to anomalous responses in the placebo condition, where physiological markers and leg extension performance indicated a failure to reach exhaustion. Including these data introduced excessive variance and compromised within‐subject comparisons.

### 3.2 Effects of MPH and REB on Performance

**FIGURE 2:**
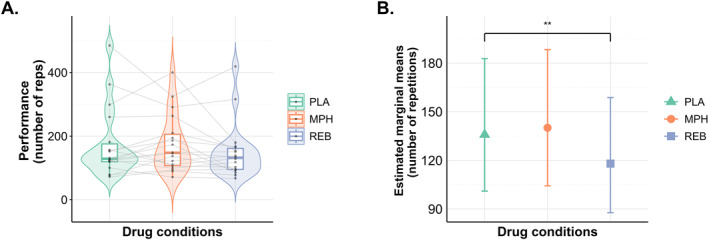
Task performance across drug conditions: Individual data and model‐based estimates. **p < 0.001. (A) Raw performance data (number of repetitions) across drug conditions, shown as individual points overlaid on violin and box plots, with group means ± standard deviations. (B) Model‐estimated marginal means (±95% confidence intervals) derived from a generalized linear mixed‐effects model accounting for within‐subject variability, sex, and learning effects. MPH: Methylphenidate targeting dopamine reuptake inhibition (2 × 10 mg Ritalin); PLA: Placebo condition (2 × 5 mg of lactose tablets); REB: Reboxetine targeting noradrenaline reuptake inhibition (2 × 4 mg Edronax).

Figure 2A shows the raw performance data (number of repetitions) across drug conditions, illustrating both individual variability and group‐level distributions. Participants completed on average 173.6 ± 110.1 repetitions under placebo, 175.9 ± 91.2 MPH, and 149.4 ± 87.7 under REB. Figure 2B shows the model‐estimated marginal means (±95% confidence intervals) derived from the generalized linear mixed‐effects model, providing adjusted estimates that account for within‐subject variability, sex, and learning effects. Model estimates indicated that, relative to placebo, REB reduced the number of leg extension repetitions by −13.2% (*p* < 0.001), whereas MPH did not influence the number of leg extension repetitions +3.1% (*p* = 0.234). These effects were observed after adjusting for individual subjects, significant learning effects (*p* < 0.001), and controlling for sex, which was not significantly associated with performance (*p* = 0.561).

### 3.3 Effects of MPH and REB on Subjective Measures

#### 3.3.1 Physical Fatigue and RPE

**FIGURE 3:**
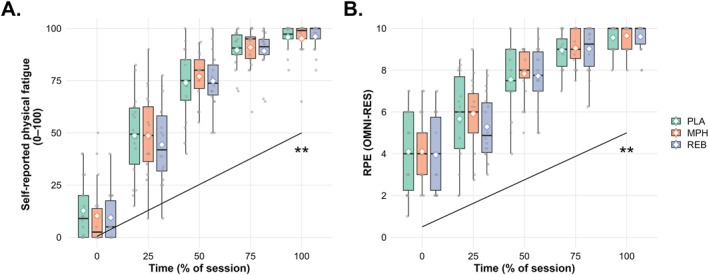
Subjective physical fatigue and rating of perceived exertion at each level of percentage failure. **p < 0.001. Self‐reported fatigue and perceived exertion across drug conditions throughout the session. (A) Physical fatigue (PVAS, 0–100) and (B) Ratings of Perceived Exertion (OMNI‐RES), 0–10) are displayed across session percentiles for each drug condition. Individual participant responses are shown as jittered points (small offsets applied to reduce overlap and improve visibility). Boxplots represent the median and interquartile range, while white‐filled markers indicate the group mean. A main effect of time was observed, with both fatigue and perceived exertion increasing across the session. No drug effects were found. MPH: Methylphenidate targeting dopamine reuptake inhibition (2 × 10 mg Ritalin); PLA: Placebo condition (2 × 5 mg of lactose tablets); REB: Reboxetine targeting noradrenaline reuptake inhibition (2 × 4 mg Edronax).

Figure 3 shows self‐reported physical fatigue and RPE across session percentiles for each drug condition. A significant main effect of time was observed, with both measures increasing progressively throughout the task (*p* < 0.001). Bonferroni‐adjusted pairwise comparisons confirmed significant differences between all percentile levels (all *p* < 0.001). Neither MPH nor REB affected physical fatigue (*p* = 0.445) or RPE (*p* = 0.521), and no significant drug × time interactions were observed.

#### 3.3.2 Motivation

Neither MPH (*p* = 0.148) nor REB (*p* = 0.926) did significantly influence self‐reported motivation prior to the leg extension task as compared to the placebo condition.

#### 3.3.3 Task Load

The overall averaged perceived task load assessed after the fatigue‐inducing task showed a significant reduction in the MPH (*p* = 0.003) and REB (*p* = 0.049) conditions compared to placebo. When examining the subscales, participants reported lower temporal demand in the MPH (*p* < 0.001) and REB (*p* < 0.001) conditions compared to placebo. The NASA‐TLX “Performance” subscale differed significantly between MPH and placebo, with participants reporting more favorable self‐evaluations of performance under MPH (*p* = 0.008). No effects of drug condition were found for “Mental” demand, “Physical” demand, or “Effort”.

#### 3.3.4 Sleepiness

Self‐reported sleepiness did not significantly change from pretask to posttask, indicating no main effect of time (*p* = 0.206). A main effect of drug was observed, with sleepiness ratings significantly lower under MPH compared to placebo (*p* = 0.004), whereas REB did not significantly alter sleepiness (*p* = 0.817). No drug × time interaction was detected.

#### 3.3.5 Mood State

Independent of drug condition, the fatigue‐inducing task elicited significant increases in self‐reported fatigue (*p* = 0.006) and vigor (*p* = 0.043). As compared to placebo, MPH administration was associated with increased self‐reported fatigue (*p* = 0.032) and vigor (*p* = 0.031), whereas REB administration only increased self‐reported tension (*p* = 0.012). Drug condition did not significantly affect self‐reported confusion (MPH: *p* = 0.752 and REB: *p* = 0.400), depression (MPH: *p* = 0.425 and REB: *p* = 0.536), or anger (MPH: *p* = 0.435 and REB: *p* = 0.711). No significant drug × time interactions were found for any subscale.

### 3.4 Effects of MPH and REB on Physiological Measures

#### 3.4.1 Heart Rate

Independent of drug condition, the fatigue‐inducing task elicited a significant increase in heart rate from pretask to posttask (*p* < 0.001). Both drug conditions significantly increased heart rate compared to placebo (MPH: *p* = 0.045 and REB: *p* < 0.001). No significant drug × time interaction was found.

#### 3.4.2 Lactate

Independent of drug condition, the fatigue‐inducing task elicited a significant increase in blood lactate levels from pretask to posttask (*p* < 0.001). Drug condition did not significantly affect lactate compared to placebo (MPH: *p* = 0.704 and REB: *p* = 0.505). No significant drug × time interaction was observed.

#### 3.4.3 Muscle Contractile Properties

Independent of drug condition, contraction velocity of the vastus medialis reduced after the fatigue‐inducing task (*p* = 0.011). No drug effects (MPH: *p* = 0.656 and REB: *p* = 0.690) or interaction effects were found for contraction velocity. For muscle displacement of the vastus medialis, no time (*p* = 0.775) drug (MPH: *p* = 0.734 and REB: *p* = 0.410) or interaction effects were found.

## 4 Discussion

This triple‐blinded randomized crossover study examined how DA and NA modulation affect performance and fatigue perception during dynamic leg extensions to exhaustion in both males and females. By integrating objective performance metrics, physiological responses, and subjective measures in a mixed‐sex cohort, we were able to compare the effects of elevated brain DA and NA via the reuptake inhibitors MPH (primarily DA) and REB (NA) with placebo. MPH and REB elicited distinct effects, offering new insights into how central catecholamines influence both physical performance and fatigue perception.

### 4.1 Confirmation of Task‐Induced Fatigue

Independent of pharmacological condition, the fatiguing task elicited time‐dependent increases in subjective and physiological markers. Exhaustion was confirmed by higher self‐reported physical fatigue, RPE, heart rate, and blood lactate post fatiguing task. Concurrently, participants reported an increase in the feeling of vigor after the task, reflecting a state of high effort accompanied by heightened arousal (St Clair Gibson and Noakes 2004). Although the post‐fatigue reduction in the contraction velocity of the vastus medialis was unexpected (Cè et al. 2020), a similar effect was reported by Kalc et al. (2023) in a comparable protocol (Kalc et al. 2023), highlighting modality‐specific neuromuscular adaptations. These findings confirm that the task successfully induced fatigue, allowing for the evaluation of drug‐specific effects.

### 4.2 The Role of DA in Physical Fatigue

To our knowledge, the present study is the first to examine DA modulation during dynamic resistance exercise, extending prior research that employed aerobic endurance paradigms (Swart et al. 2009; Roelands, Hasegawa, et al. 2008; Klass et al. 2012; Watson et al. 2005; Roelands et al. 2012; Cordery et al. 2017; Roelands and Meeusen 2010). This distinction is important because resistance exercise imposes distinct physiological demands. In dynamic leg extensions, performance is primarily constrained by localized, high intensity muscle contractions and motor unit recruitment (Dinyer et al. 2022), rather than by whole‐body systemic fatigue (Tornero‐Aguilera et al. 2022). In the present study, MPH did not significantly improve leg extension performance, suggesting that elevated brain DA was insufficient to overcome the peripheral and neuromuscular constraints of prolonged resistance exercise. Importantly, these effects were consistent after adjusting for individual subject variability and learning effects, with sex not significantly associated with performance. This suggests that the absence of a performance benefit reflects task‐specific physiological constraints rather than sex differences (Dinyer 2020).

Nonetheless, MPH had pronounced effects on cognitive–perceptual outcomes. MPH predominantly modulates central fatigue via DA reuptake inhibition (Dockree et al. 2017). DA is critically involved in maintaining wakefulness and alertness through projections from the ventral tegmental area to cortical and subcortical regions (Zheng and Hasegawa 2017), which likely underpins the observed reductions in sleepiness and increases in state of vigor. DA also contributes to the subjective valuation of effort (Zheng and Hasegawa 2017; Volkow et al. 2011), biasing individuals to perceive tasks as less demanding or more rewarding even in the absence of objective performance changes (Westbrook and Braver 2016; Salamone and Correa 2012; Westbrook and Braver 2015). The decreased temporal demand, perceived success in performance, and lower overall task load observed in this study appear to reflect this cognitive–perceptual modulation, whereby participants experienced the task as more manageable without a concomitant improvement in physical capacity.

Importantly, these effects could be context dependent. The present fatiguing task was repetitive and monotonous, with no explicit reward or incentive, potentially limiting the influence of DA modulation on performance (Michely et al. 2020). Supporting this, self‐reported motivation declined over time across trials, suggesting that the task may not have been optimally sensitive to DA‐driven motivational effects (Zheng and Hasegawa 2017). Prior studies in aerobic exercise under normal ambient conditions (Roelands, Hasegawa, et al. 2008; Klass et al. 2012) have reported similar findings, with no improvement in performance under MPH. In contrast, when prolonged endurance exercise is performed under heat stress (Roelands, Hasegawa, et al. 2008), the ergogenic effects of DA seem more pronounced, likely through sustained central drive, enhanced arousal, and thermoregulatory support (Meeusen and Roelands 2010). Collectively, these results indicate that although DA can modulate cognitive‐perceptual aspects of task engagement, its influence on localized neuromuscular output may be limited under prolonged resistance exercise conditions.

### 4.3 The Role of NA in Physical Fatigue

REB administration led to a −13.2% reduction in the number of leg extension repetitions. This is consistent with prior evidence that elevated noradrenaline impairs sustained submaximal efforts during repeated isometric knee extensions (Klass et al. 2016) and endurance exercise (Klass et al. 2012; Roelands, Goekint, et al. 2008). Importantly, our study extends these findings to dynamic contractions performed to exhaustion, demonstrating that the detrimental effects of increased NA are not confined to isolated isometric or endurance paradigms. These results highlight that NA modulation is a critical determinant of performance, shaping the capacity to sustain force output independent of sex or task‐specific neuromuscular characteristics.

The performance decrement likely reflects excessive elevated synaptic NA beyond the optimal α2A‐adrenoceptor regulation in the prefrontal cortex, activating low affinity α1‐adrenoceptors that reduce motor drive and performance (Xing et al. 2016; Arnsten 2011). Unlike modest peripheral effects of MPH, REB's potent noradrenergic action exerts strong peripheral sympathetic effects (A. W. Tank and Lee Wong 2015; J. Tank et al. 2003), which may have further compromised task performance.

Notably, this performance decline occurred without substantial changes in self‐reported fatigue, RPE, motivation, sleepiness, or mood (aside from increased tension). Overall perceived task load was reduced, possibly due to lower temporal demand, suggesting earlier task disengagement and a shortened time to exhaustion. This decoupling between performance and perception indicates that neuromuscular limitations can be masked, potentially impairing self‐monitoring and adaptive pacing during fatiguing tasks. While prior studies have suggested a disconnect between perceptual feedback and motor output following NA modulation (Meeusen and Roelands 2018; Klass et al. 2012, 2016), our study provides the first evidence of this phenomenon in dynamic exercise to exhaustion.

### 4.4 Limitations

We used fixed absolute doses of MPH and REB rather than body mass‐adjusted doses. Although this may not fully account for individual differences in body mass, our sample was relatively homogeneous and the doses were consistent with prior studies, allowing direct comparison. Furthermore, understanding the neurochemical basis of central fatigue remains a complex challenge. Given the overlapping and interactive roles of DA and NA (Zheng and Hasegawa 2017), and the use of selective reuptake inhibitors, these findings should be interpreted within the context of their limitations. Despite the relative selectivity of MPH and REB for DA and NA systems (Berridge and Devilbiss 2011), both drugs have off‐target effects on other monoaminergic pathways (Miller et al. 2002; King et al. 2018). In regions rich in noradrenergic innervation, such as the medial prefrontal cortex (Takano et al. 2008), DA concentrations can be modulated indirectly via NA transporters, reflecting a complex neurochemical interplay (Klass et al. 2012). Moreover, other types of fatigue, such as mental fatigue, may share overlapping but distinct neurochemical mechanisms, which were not taken into account (Meeusen and Roelands 2018).

### 4.5 Future Directions

Future research should examine how factors, such as task type, motivation, and environmental stress, interact with neurotransmitter systems to influence performance. Incorporating electrophysiological or neuroimaging techniques could clarify how catecholamine activity relates to perception, motivation, and motor output. Additionally, studies should investigate how neurotransmitters contribute to the interaction between physical and mental fatigue.

### 4.6 Conclusion

This study provides novel evidence on how brain DA and NA shape performance and perception during fatiguing exercise in both males and females using a dynamic leg extension task. DA stimulation enhanced alertness, vigor, and task engagement without improving physical output, highlighting its role in cognitive‐perceptual rather than motor aspects of fatigue. In contrast, NA stimulation impaired performance while leaving subjective fatigue largely unchanged, indicating a dissociation between perceived effort and actual capacity. Together, these findings offer new insight into the neurochemical basis of physical fatigue and emphasize that the balance and context‐specific modulation of these neurotransmitters is critical for optimizing performance and managing fatigue.

## Author Contributions

Conceptualization was carried out by Y.L.A.A., K.D.P., and B.R. funding acquisition was managed by B.R. and UM. resources were provided by K.D.P., U.M., and B.R. project administration was handled by Y.L.A.A. methodology development involved Y.L.A.A., J.H., K.D.P., and B.R. The investigation was conducted by Y.L.A.A. and A.M. formal analyses were performed by Y.L.A.A. and E.L. visualization was completed by Y.L.A.A. The original draft of the manuscript was written by Y.L.A.A., with review and editing contributions from R.M., L.S.F., J.H., E.L., K.D.P., and B.R. data curation was carried out by YLAA. supervision and oversight were provided by UM, KDP, and BR. All authors reviewed and approved the final manuscript. All individuals listed as authors meet the criteria for authorship, and all who qualify have been included.

## Funding

Yahaira Laurisa Arenales Arauz is a recipient of a doctoral grant funded by FWO_WEAVE (G095422N). Kevin De Pauw and Bart Roelands also acknowledge financial support from the European Union (HORIZON‐CSA‐101120150). Jelle Habay is a recipient of a fundamental aspirant fellowship funded by the Research Foundation Flanders (FWO) (project number: 11J6323N). The authors would also like to acknowledge the funding under the Horizon Europe—TBrainBoost, grant agreement No. 101120150.

## Ethics Statement

This study was approved by the commission of medical ethics at the UZ Brussel (protocol: 2022‐002836‐30) in accordance with the declaration of Helsinki. Informed consent was obtained from the participants.

## Consent

All participants gave written informed consent to participate in the present study.

## Conflicts of Interest

Y. Laurisa Arenales Arauz, Ana Mali, Elke Lathouwers, Jelle Habay, Leonardo de Sousa Fortes, Romain Meeusen, Uros Marusic, Kevin De Pauw, and Bart Roelands have no conflicts of interest of any type (i.e. financial, professional, or personal) relevant to the content of this study.

## Permission to Reproduce Material From Other Sources

The authors have nothing to report.

## Supporting information


**Supporting Information S1:** Complete raw dataset for all outcome measures. Raw anthropometric, subjective, behavioral, and physiological data for all participants included in the analyses under each drug condition.

**Supporting Information S2:** R script for statistical analyses. Annotated R script containing data import procedures, custom functions, data description and visualization, model selection, and model assumption checks.

## Data Availability

The data that supports the findings of this study are available in the supplementary material of this article.
