# Supplementary material for: Distinct Roles of Dopamine and Noradrenaline in Physical Fatigue
Source: Eur J Sport Sci. 2026 Jan 27;26(2):e70119. doi: 10.1002/ejsc.70119 (PMC12848314; doi:10.1002/ejsc.70119)
Supplement: Supplementary file 2 — Supporting Information S2: R script for statistical analyses. Annotated R script containing data import procedures, custom functions, data description and visualization, model selection, and model assumption checks. [file EJSC-26-e70119-s001.pdf]

Dataset_WP2

Laurisa Arenales

2024-07-12

PAPER 1 "Distinct Effects of Dopamine and Noradrenaline Modulation on Exercise-induced Fatigue and Performance"

Load libraries

```
library(readxl)
library(psych)
library(ggplot2)
```

```
##
## Attaching package: 'ggplot2'

## The following objects are masked from 'package:psych':
##
##     %+%, alpha
```

```
library(ggpubr)
```

```
## Warning: package 'ggpubr' was built under R version 4.4.3
```

```
library(tidyverse)
```

```
## -- Attaching core tidyverse packages ------------------------ tidyverse 2.0.0 --
## v dplyr     1.1.4     v readr     2.1.5
## v forcats   1.0.0     v stringr   1.5.1
## v lubridate 1.9.3     v tibble    3.2.1
## v purrr     1.0.2     v tidyr     1.3.1

## -- Conflicts ------------------------------------------ tidyverse_conflicts() --
## x ggplot2::%+%()   masks psych::%+%()
## x ggplot2::alpha() masks psych::alpha()
## x dplyr::filter()  masks stats::filter()
## x dplyr::lag()     masks stats::lag()
## i Use the conflicted package (<http://conflicted.r-lib.org/>) to force all conflicts to become error[illegible]
```

```r
library(ggpubr)
library(rstatix)
```

```
##
## Attaching package: 'rstatix'
##
## The following object is masked from 'package:stats':
##
##     filter
```

```r
library(lme4)
```

```
## Loading required package: Matrix
##
## Attaching package: 'Matrix'
##
## The following objects are masked from 'package:tidyr':
##
##     expand, pack, unpack
```

```r
library(lmerTest)
```

```
## Warning: package 'lmerTest' was built under R version 4.4.3
```

```
##
## Attaching package: 'lmerTest'
##
## The following object is masked from 'package:lme4':
##
##     lmer
##
## The following object is masked from 'package:stats':
##
##     step
```

```r
library(emmeans)
```

```
## Welcome to emmeans.
## Caution: You lose important information if you filter this package's results.
## See '? untidy'
```

```r
library(sjPlot)
library(readxl)
library(rlang)
```

```
##
## Attaching package: 'rlang'
##
## The following objects are masked from 'package:purrr':
##
##     %@%, flatten, flatten_chr, flatten_dbl, flatten_int, flatten_lgl,
##     flatten_raw, invoke, splice
```

```r
library(pals)
library(grid)
library(gridExtra)
```

```
##
## Attaching package: 'gridExtra'
##
## The following object is masked from 'package:dplyr':
##
##     combine
```

```r
library(scales)
```

```
##
## Attaching package: 'scales'
##
## The following object is masked from 'package:purrr':
##
##     discard
##
## The following object is masked from 'package:readr':
##
##     col_factor
##
## The following objects are masked from 'package:psych':
##
##     alpha, rescale
```

```r
library(patchwork)
```

```
## Warning: package 'patchwork' was built under R version 4.4.3
```

```r
library(lmtest)
```

```
## Warning: package 'lmtest' was built under R version 4.4.3
```

```
## Loading required package: zoo
```

```
## Warning: package 'zoo' was built under R version 4.4.3
```

```
##
## Attaching package: 'zoo'
##
## The following objects are masked from 'package:base':
##
##     as.Date, as.Date.numeric
```

```r
library(DHARMa)
```

```
## Warning: package 'DHARMa' was built under R version 4.4.3

## This is DHARMa 0.4.7. For overview type '?DHARMa'. For recent changes, type news(package = 'DHARMa')
```

```r
library(glmmTMB)
```

```
## Warning: package 'glmmTMB' was built under R version 4.4.3
```

```r
library(MASS)
```

```
##
## Attaching package: 'MASS'
##
## The following object is masked from 'package:patchwork':
##
##     area
##
## The following object is masked from 'package:rstatix':
##
##     select
##
## The following object is masked from 'package:dplyr':
##
##     select
```

```r
library(ARTool)
```

```
## Warning: package 'ARTool' was built under R version 4.4.3
```

```r
library(ordinal)
```

```
## Warning: package 'ordinal' was built under R version 4.4.3

##
## Attaching package: 'ordinal'
##
## The following object is masked from 'package:dplyr':
##
##     slice
```

```r
library(ez)
```

```
## Warning: package 'ez' was built under R version 4.4.3
```

```r
library(ggeffects)
```

```
## Warning: package 'ggeffects' was built under R version 4.4.3
```

```
library(brant)
```

```
## Warning: package 'brant' was built under R version 4.4.3
```

```
library(viridis)
```

```
## Warning: package 'viridis' was built under R version 4.4.3
```

```
## Loading required package: viridisLite
##
## Attaching package: 'viridisLite'
##
## The following objects are masked from 'package:pals':
##
##     cividis, inferno, magma, plasma, turbo, viridis
##
##
## Attaching package: 'viridis'
##
## The following object is masked from 'package:scales':
##
##     viridis_pal
##
## The following objects are masked from 'package:pals':
##
##     cividis, inferno, magma, plasma, turbo, viridis
```

1. DATA IMPORT AND ORGANIZATION

```
#----------------------------------- Import Data -----------------------------------------------------

# Define path to folder and file
path <- "C:/Users/YA000015/Workspace_R/Data WP2"
file <- "Data_WP2.xlsx"

# Load full dataset
Data_WP2 <- read_excel(file.path(path, file))

# Load specific sheets

data_antro  <- read_excel(file.path(path, file), sheet = "antropometrics") # participant characteristic
data_perf   <- read_excel(file.path(path, file), sheet = "perf") #  performance, motivas, internal work
data_pvas_rpe <- read_excel(file.path(path, file), sheet = "pvas_rpe") # pvas, rpe
```

```
## New names:
## * `` -> `...6`
## * `` -> `...7`
## * `` -> `...8`
## * `` -> `...9`
```

```
## * `` -> `...10`
## * `` -> `...11`
## * `` -> `...12`
## * `` -> `...13`
## * `` -> `...14`
## * `` -> `...15`
```

```r
data_subj   <- read_excel(file.path(path, file), sheet = "subj") # sleepiness, # mood state
data_tmg <- read_excel(file.path(path, file), sheet = "tmg") # contraction time, dm
data_phys   <- read_excel(file.path(path, file), sheet = "phys") # delta HR, delta lactate

#----------------------------------- Organize Datasets for Analysis -----------------------------------


# Convert variables into factors and give meaningful labels

data_antro$doctor_gender <- as.numeric(data_antro$doctor_gender)

data_antro$doctor_gender <- factor(data_antro$doctor_gender, levels = c(1,2), labels = c("Male", "Femal

data_perf$drug <- factor(data_perf$drug, levels = c(1, 2, 3),
                         labels = c("PLA", "MPH", "REB"))
data_perf$di_mot <- factor(data_perf$di_mot, levels = c(0, 1),
                         labels = c("low", "high"))
data_perf$gender <- factor(data_perf$gender, levels = c(1, 2),
                         labels = c("male", "female"))
data_perf$visit <- factor(data_perf$visit, levels = c(1, 2, 3),
                         labels = c("visit 1", "visit 2", "visit 3"))
data_pvas_rpe$percentile <- factor(data_pvas_rpe$percentile, levels = c(0, 25, 50, 75, 100),
                         labels = c("0", "25", "50", "75", "100"))
data_pvas_rpe$drug <- factor(data_pvas_rpe$drug, levels = c(1, 2, 3),
                         labels = c("PLA", "MPH", "REB"))
data_subj$drug <- factor(data_subj$drug, levels = c(1, 2, 3),
                         labels = c("PLA", "MPH", "REB"))
data_tmg$drug <- factor(data_tmg$drug, levels = c(1, 2, 3),
                         labels = c("PLA", "MPH", "REB"))
data_phys$drug <- factor(data_phys$drug, levels = c(1, 2, 3),
                         labels = c("PLA", "MPH", "REB"))
data_subj$time <- factor(data_subj$time, levels = c(1, 2),
                         labels = c("pre-fatigue", "post-fatigue"))


# Filter to keep only 0 and 1 for time points --> we do not need the other timepoints for analysis
# TMG
data_tmg_sub <- subset(data_tmg, time %in% c(0, 1))
# Convert 'time' to a factor with descriptive labels
data_tmg_sub$time <- factor(data_tmg_sub$time,
                            levels = c(0, 1),
                            labels = c("baseline", "post-fatigue"))

# Lactate and HR
data_phys_sub <- subset(data_phys, type %in% c(1,2))
# Convert 'time' to a factor with descriptive labels
data_phys_sub$type <- factor(data_phys_sub$type,
```

```r
                            levels = c(1,2),
                            labels = c("pre", "post"))



# Check data structures
str(data_perf$drug)
```

```
##  Factor w/ 3 levels "PLA","MPH","REB": 3 1 2 2 1 3 3 2 1 2 ...
```

```r
str(data_pvas_rpe$drug)
```

```
##  Factor w/ 3 levels "PLA","MPH","REB": 3 3 3 3 3 1 1 1 1 1 ...
```

```r
str(data_subj$drug)
```

```
##  Factor w/ 3 levels "PLA","MPH","REB": 3 3 1 1 2 2 2 2 1 1 ...
```

```r
str(data_tmg$drug)
```

```
##  Factor w/ 3 levels "PLA","MPH","REB": 1 1 1 1 1 2 2 2 2 2 ...
```

```r
str(data_phys$drug)
```

```
##  Factor w/ 3 levels "PLA","MPH","REB": 3 3 3 1 1 1 2 2 2 2 ...
```

```r
str(data_subj$time)
```

```
##  Factor w/ 2 levels "pre-fatigue",..: 1 2 1 2 1 2 1 2 1 2 ...
```

```r
str(data_pvas_rpe$percentile)
```

```
##  Factor w/ 5 levels "0","25","50",..: 1 2 3 4 5 1 2 3 4 5 ...
```

```r
str(data_tmg_sub)
```

```
## tibble [108 x 5] (S3: tbl_df/tbl/data.frame)
##  $ record_id: num [1:108] 3 3 3 3 3 3 8 8 8 8 ...
##  $ drug     : Factor w/ 3 levels "PLA","MPH","REB": 1 1 2 2 3 3 1 1 2 2 ...
##  $ time     : Factor w/ 2 levels "baseline","post-fatigue": 1 2 1 2 1 2 1 2 1 2 ...
##  $ tc       : num [1:108] 19.8 20.8 20 20.7 19.9 ...
##  $ dm       : num [1:108] 5.89 5.61 7.24 4.69 7.47 ...
```

```r
str(data_phys_sub)
```

```
## tibble [108 x 5] (S3: tbl_df/tbl/data.frame)
##  $ record_id: num [1:108] 3 3 3 3 3 3 8 8 8 8 ...
##  $ drug     : Factor w/ 3 levels "PLA","MPH","REB": 3 3 1 1 2 2 2 2 1 1 ...
##  $ type     : Factor w/ 2 levels "pre","post": 1 2 1 2 1 2 1 2 1 2 ...
##  $ lactate  : num [1:108] 4.31 6.52 3.94 4.19 2.59 2.34 2.2 1.79 4.41 5.62 ...
##  $ hr       : num [1:108] 122 183 96 171 111 181 104 172 123 165 ...
```

2. PARTICIPANT CHARACTERISTICS

```r
#------------------------------------ AGE, GENDER, BMI, fat percentage------------------------------------

# Variables of interest
vars_antro <- c("doctor_gender", "doctor_age", "bmi", "vetpercentage")

# Subset the data
data_subset <- data_antro[ , vars_antro]

# Compute mean and SD
mean_sd <- sapply(data_subset, function(x) {
  if (is.numeric(x)) {
    c(Mean = mean(x, na.rm = TRUE), SD = sd(x, na.rm = TRUE))
  } else {
    c(Mean = NA, SD = NA)
  }
})

# Print Mean and SD
cat("Means and Standard Deviations:\n")
```

```
## Means and Standard Deviations:
```

```r
print(t(mean_sd))  # Transpose for readability
```

```
##                     Mean       SD
## doctor_gender         NA       NA
## doctor_age    23.38889 2.199970
## bmi           22.85556 2.603216
## vetpercentage 20.81111 8.526560
```

```r
# Print the usual summary
cat("\nSummary Statistics:\n")
```

```
##
## Summary Statistics:
```

```r
print(summary(data_subset))
```

```
##  doctor_gender   doctor_age         bmi         vetpercentage
##  Male  :9      Min.   :20.00   Min.   :18.40   Min.   :10.70
##  Female:9      1st Qu.:22.00   1st Qu.:21.80   1st Qu.:13.82
##                Median :23.00   Median :22.70   Median :18.85
##                Mean   :23.39   Mean   :22.86   Mean   :20.81
##                3rd Qu.:25.00   3rd Qu.:23.27   3rd Qu.:26.50
##                Max.   :28.00   Max.   :30.90   Max.   :41.40
```

```r
# Set layout to plot 3 boxplots side-by-side
par(mfrow = c(1, 3))
boxplot(data_antro$bmi,
        main = "BMI", ylab = "BMI", col = "skyblue") # Boxplot for BMI
boxplot(data_antro$vetpercentage,
        main = "Fat Percentage", ylab = "Vetpercentage", col = "lightgreen") # Boxplot for vetpercentag
boxplot(data_antro$doctor_age,
        main = "Doctor Age", ylab = "Age", col = "lightcoral") # Boxplot for doctor age
```

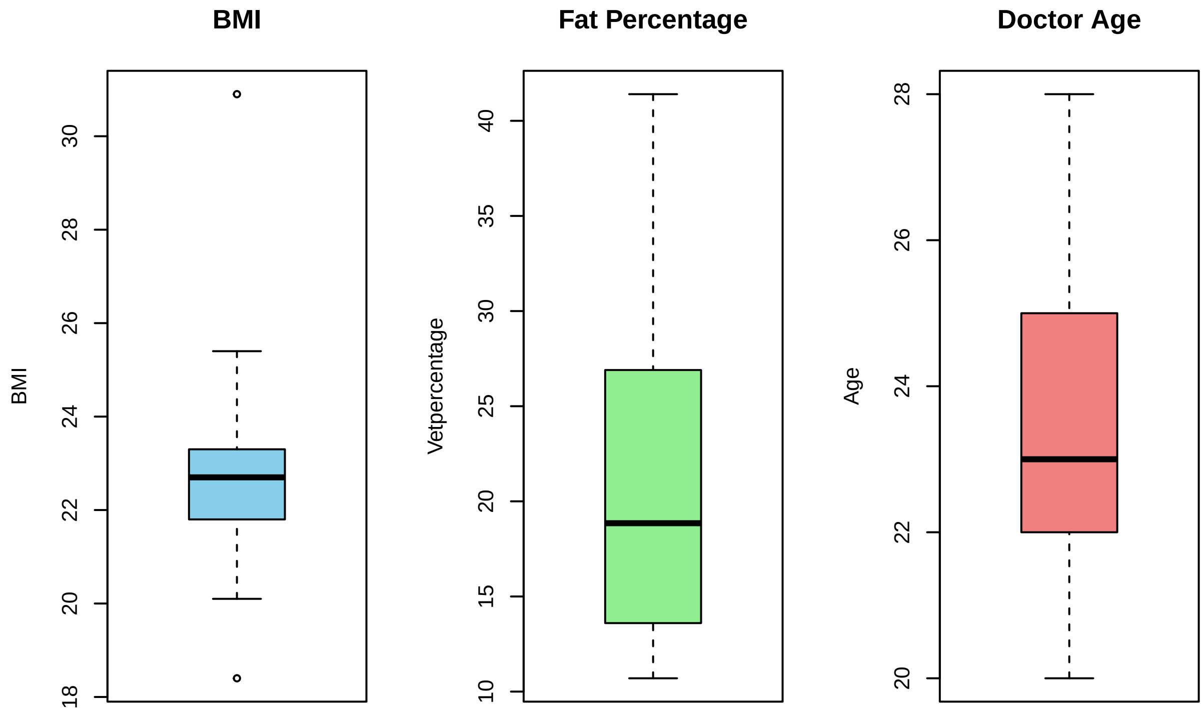

3. FUNCTIONS

```r
# the functions below are made to show the estimates of the original value of each of the models + cond


# -----------------------------------------------DATA EXTRACTION TABLE FUNCTIONs for LMER models
# this function is for all lmer models

# add below as functions!
#summary_log <- summarize_lmer_effects(model_log, inverse = exp)
#summary_sqrt <- summarize_lmer_effects(model_tmgtc_sqrt, inverse = function(x) x^2)
#summary_plain <- summarize_lmer_effects(model_plain)
```

```r
summarize_lmer_effects <- function(model, inverse = identity, digits = 2) {
  fe <- summary(model)$coefficients
  est <- fe[, "Estimate"]
  se <- fe[, "Std. Error"]

  # Detect correct p-value column
  pval_col <- grep("Pr\\(>", colnames(fe), value = TRUE)
  pvals <- if (length(pval_col) > 0) fe[, pval_col] else NA

  lower <- est - 1.96 * se
  upper <- est + 1.96 * se

  # Apply inverse transformation
  est_inv <- inverse(est)
  lower_inv <- inverse(lower)
  upper_inv <- inverse(upper)

  data.frame(
    Term = rownames(fe),
    Estimate = round(est, digits),
    SE = round(se, digits),
    Estimate_inv = round(est_inv, digits),
    CI_lower = round(lower_inv, digits),
    CI_upper = round(upper_inv, digits),
    p_value = round(pvals, 3),
    row.names = NULL
  )
}
```

```r
# -----------------------------------------------DATA EXTRACTION TABLE FUNCTION GLMM PERFORMANCE
# This function is FOR THE GLMER MODEL OF COUNT DATA POISSON

# add this as function!
# report_glmer_effects(model) --> add this as function!

report_glmer_effects <- function(model) {
  fe <- summary(model)$coefficients
  est <- fe[, "Estimate"]
  se <- fe[, "Std. Error"]
  pvals <- fe[, "Pr(>|z|)"]  # works for glmer/glm; if lmer, might be "Pr(>|t|)"

  lower <- est - 1.96 * se
  upper <- est + 1.96 * se

  est_exp <- exp(est)
  lower_exp <- exp(lower)
  upper_exp <- exp(upper)

  data.frame(
    Term = rownames(fe),
    Estimate_log = est,
    SE_log = se,
    Estimate_exp = est_exp,
```

```r
    CI_lower = lower_exp,
    CI_upper = upper_exp,
    p_value = pvals,
    row.names = NULL
  )
}
```

```r
# ---------------------------------------------------------------------------------function for GLMMTMB
# add this as function!
# report_glmTMB_effects(model)


report_glmmTMB_effects <- function(model) {
  fe <- summary(model)$coefficients$cond  # 'cond' = conditional model part
  est <- fe[, "Estimate"]
  se <- fe[, "Std. Error"]
  pvals <- fe[, "Pr(>|z|)"]

  # 95% Confidence Intervals
  lower <- est - 1.96 * se
  upper <- est + 1.96 * se

  # Exponentiate for IRRs
  est_exp <- exp(est)
  lower_exp <- exp(lower)
  upper_exp <- exp(upper)

  data.frame(
    Term = rownames(fe),
    Estimate_log = round(est, 3),
    SE_log = round(se, 3),
    IRR = round(est_exp, 2),
    CI_lower = round(lower_exp, 2),
    CI_upper = round(upper_exp, 2),
    p_value = signif(pvals, 3),
    row.names = NULL
  )
}
```

```r
# ---------------------------------------------------------function for ORDINAL MODEL
# add this as function!
# report_clmm_effects(model_sleep_ord)


report_clmm_effects <- function(model) {
  coefs <- coef(summary(model))  # safer way to extract all coefficients

  est <- coefs[, "Estimate"]
  se <- coefs[, "Std. Error"]
  zvals <- coefs[, "z value"]
  pvals <- 2 * (1 - pnorm(abs(zvals)))  # calculate p-values

  # Confidence intervals (log-odds scale)
```

```
  lower <- est - 1.96 * se
  upper <- est + 1.96 * se

  # Exponentiate for Odds Ratios (for predictors only)
  term_names <- rownames(coefs)
  is_threshold <- grepl("\\|", term_names)

  est_exp <- exp(est)
  lower_exp <- exp(lower)
  upper_exp <- exp(upper)

  df <- data.frame(
    Term = term_names,
    Estimate_log = round(est, 3),
    SE_log = round(se, 3),
    OR = ifelse(is_threshold, NA, round(est_exp, 2)),
    CI_lower = ifelse(is_threshold, NA, round(lower_exp, 2)),
    CI_upper = ifelse(is_threshold, NA, round(upper_exp, 2)),
    p_value = signif(pvals, 3),
    row.names = NULL
  )

  return(df)
}
```

```
# BELOW ARE THE DESCRIPTIVES, VISUALIZATION, MODELS AND MODEL ASSUMPTION CHECKS OF ALL OUTCOME MEASURES
# I TAKE THE SAME STEPS EVERYTIME!

# 1) COMPUTE DESCRIPTIVES OF OUTCOME MEASURES
# 2) DECIDE ON THE NATURE OF THE DATA + VISUALIZE DATA USING BOXPLOTS AND HISTOGRAMS TO CHECK THE DISTR
# 3) BASED ON THE NATURE AND DISTRIBUTION OF THE DATA THE PROPER MODEL WAS CHOSEN
# 4) MODEL ASSUMPTIONS WERE CHECKED USING DHARMA PACKAGE ( normality of residuals, homoscedacity etc)
# 5) IF ASSUMPTIONS WERE NOT MET WE TRANSFORMED THE DATA (LOG, sqrt..) AND REMADE THE MODEL PLUS CHECKE
# 6) IF ASSUMPTIONS WERE STILL NOT MET, WE USED ALTERNATIVE MODELS
# 7) PLOTS OF MODELS WERE ALSO MADE WHERE NECESSARY

#_________________________________________________________________________________
# MAIN OUTCOME MEAUSRES ARE PERFORMANCE AND PERCEIVED FATIGUE
# SECONDARY OUTCOME MEASURES INCLUDE MOTIVATION, MOOD, INTERNAL WORKLOAD, AND PHYSIOLOGICAL PARAMETERS
```

4. DATA

4.1 PERFORMANCE

4.11 DATA DESCRIPTION AND VISUALIZATION (PERFORMANCE)

```
# ----------------------------------------PERFORMANCE IN N REPS----------------------------------------

# DESCRIPTIVES --> one value post fatigue. this is the number of repetitions on the leg extensions that
```

```r
# Compute descriptives mean and standard deviation
mean_data_perf <- data_perf %>%
  group_by(drug) %>%
  summarise(
    mean = round(mean(rep_nr, na.rm = TRUE), 2),
    sd = round(sd(rep_nr, na.rm = TRUE), 2)
  )
view(mean_data_perf)
```

```r
# ---------------------------------------------------VISUALIZATION --> Boxplot with jittered raw data po
ggplot(data_perf, aes(x = drug, y = rep_nr)) +
  geom_boxplot(fill = "white", color = "darkblue", outlier.shape = NA) +
  geom_jitter(aes(color = drug),
              position = position_jitterdodge(jitter.width = 0.2, dodge.width = 0.8),
              size = 1.5, alpha = 0.6) +  # Raw data points jittered
  labs(title = "Repetitions per Drug with Group Means",
       x = "Drug Condition",
       y = "Number of Repetitions") +
  theme_minimal() +
  theme(legend.position = "none")  # Remove legend for color since drug is on x-axis
```

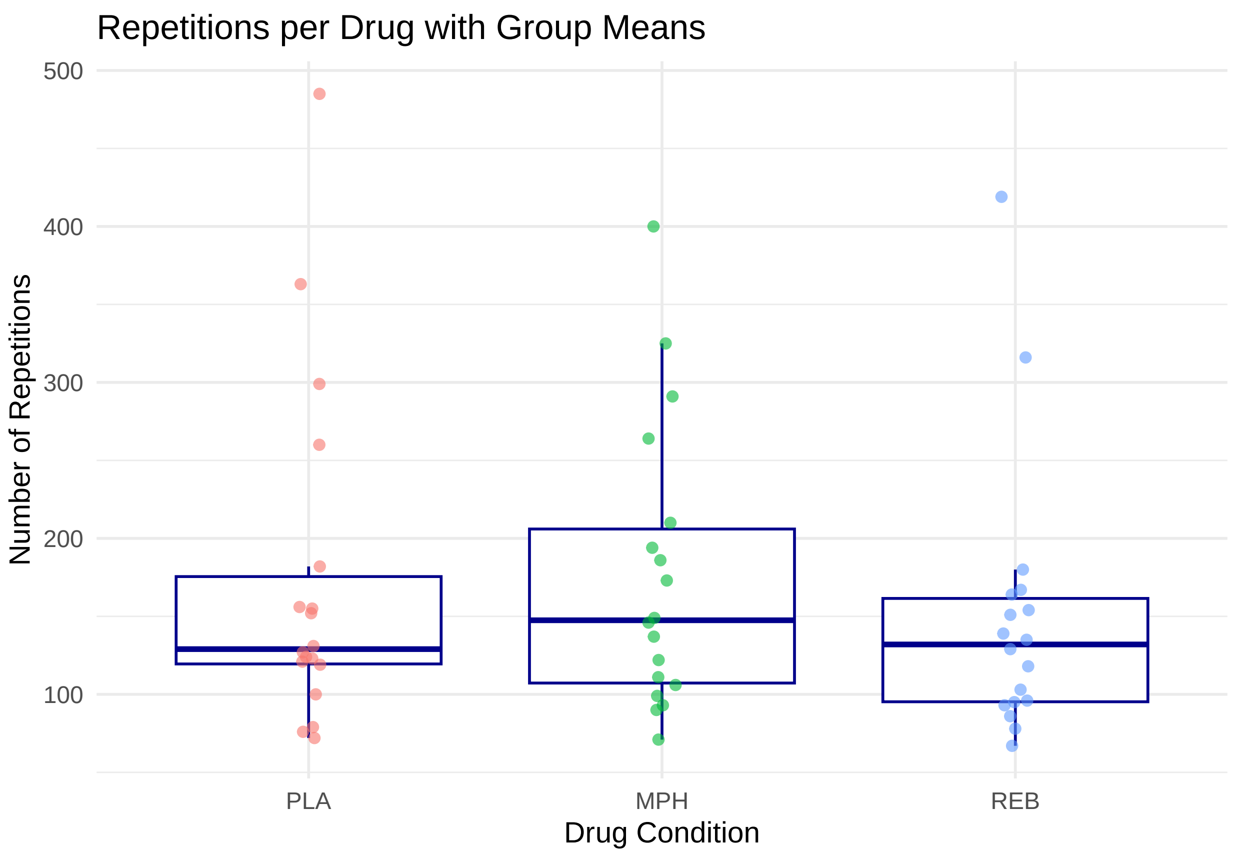


```r
# Histogram showing distribution of repetitions by drug condition
ggplot(data_perf, aes(x = rep_nr, fill = drug)) +
  geom_histogram(binwidth = 25, position = "dodge", color = "black", alpha = 0.7) +
  labs(title = "Distribution of Repetitions by Drug Condition",
```

```
      x = "Number of Repetitions",
      y = "Count") +
  theme_minimal() +
  scale_fill_brewer(palette = "Set2")
```

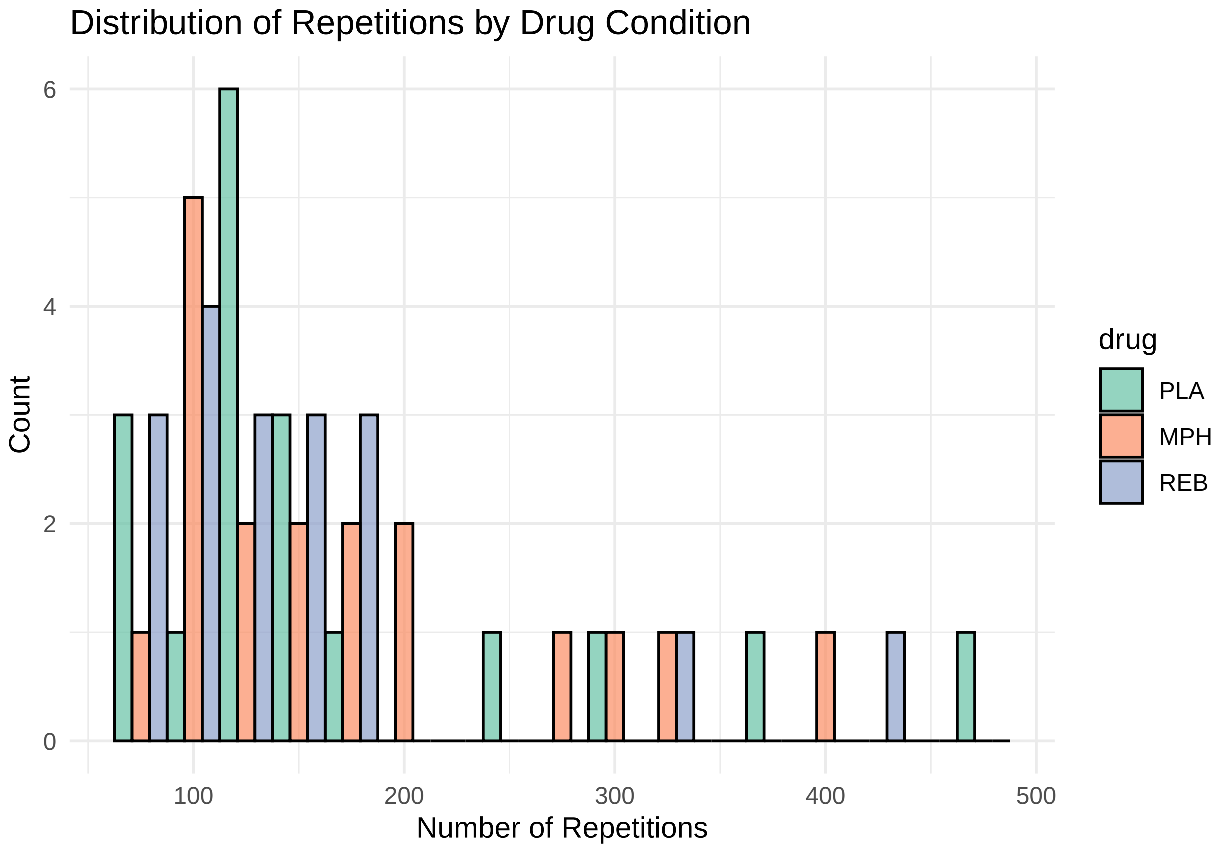


```
# INDIVIDUAL TRAJECTORIES --> i added this gg plot to check on outliers of data. There was one particip
ggplot(data_perf, aes(x = drug, y = rep_nr, group = record_id)) +
  geom_jitter(aes(color = drug),
              position = position_jitterdodge(jitter.width = 0.2, dodge.width = 0.8),
              size = 1.5, alpha = 0.6) +
  geom_line(data = subset(data_perf, drug %in% c("REB", "PLA", "MPH")),
            aes(group = record_id),
            position = position_dodge(width = 0.8),
            color = "gray50", alpha = 0.5) +
  scale_x_discrete(limits = c("REB", "PLA", "MPH")) +  # Reorder x-axis for plotting
  labs(title = "Repetitions per Drug with Group Means",
       x = "Drug Condition",
       y = "Number of Repetitions") +
  theme_minimal() +
  theme(legend.position = "none")
```

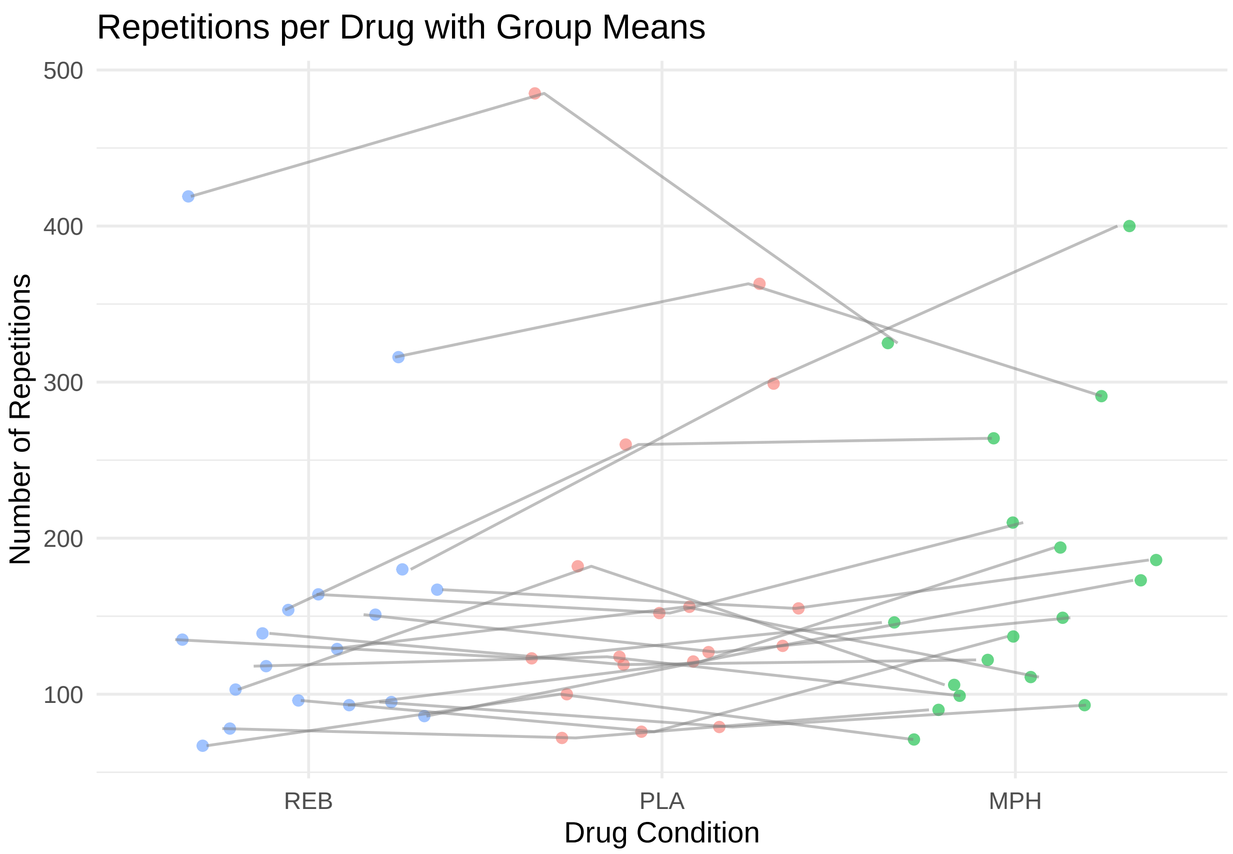

4.12 DATA ANALYSIS (PERFORMANCE)

```
# PERFORMANCE: How do different treatment conditions influence the number of leg extensions performed by

model_performance1 <- glmer(rep_nr ~ drug + (1 | record_id), data = data_perf, family = poisson())
summary(model_performance1) # this model takes the record ID in consideration
```

```
## Generalized linear mixed model fit by maximum likelihood (Laplace
##   Approximation) [glmerMod]
##  Family: poisson  ( log )
## Formula: rep_nr ~ drug + (1 | record_id)
##    Data: data_perf
##
##      AIC      BIC   logLik deviance df.resid
##    749.7    757.7   -370.9    741.7       50
##
## Scaled residuals:
##     Min      1Q  Median      3Q     Max
## -5.1322 -1.5937  0.2916  1.5829  5.1906
##
## Random effects:
##  Groups    Name        Variance Std.Dev.
##  record_id (Intercept) 0.2018   0.4492
## Number of obs: 54, groups:  record_id, 18
```

```
##
## Fixed effects:
##             Estimate Std. Error z value Pr(>|z|)
## (Intercept)  5.04574    0.10749  46.940  < 2e-16 ***
## drugMPH      0.01367    0.02520   0.542    0.588
## drugREB     -0.14957    0.02629  -5.689 1.28e-08 ***
## ---
## Signif. codes:  0 '***' 0.001 '**' 0.01 '*' 0.05 '.' 0.1 ' ' 1
##
## Correlation of Fixed Effects:
##         (Intr) drgMPH
## drugMPH -0.118
## drugREB -0.113  0.483
```

```r
# learning effects and gender were included to control for potential influence
model_performance2 <- glmer(rep_nr ~ drug + visit + gender +  (1 | record_id), data = data_perf, family
summary(model_performance2)
```

```
## Generalized linear mixed model fit by maximum likelihood (Laplace
##   Approximation) [glmerMod]
##  Family: poisson  ( log )
## Formula: rep_nr ~ drug + visit + gender + (1 | record_id)
##    Data: data_perf
##
##      AIC      BIC   logLik deviance df.resid
##    738.5    752.4   -362.2    724.5       47
##
## Scaled residuals:
##     Min      1Q  Median      3Q     Max
## -4.1733 -1.7501  0.0806  2.0268  4.5091
##
## Random effects:
##  Groups    Name        Variance Std.Dev.
##  record_id (Intercept) 0.1986   0.4457
## Number of obs: 54, groups:  record_id, 18
##
## Fixed effects:
##              Estimate Std. Error z value Pr(>|z|)
## (Intercept)   4.91188    0.15128  32.470  < 2e-16 ***
## drugMPH       0.03049    0.02559   1.191 0.233596
## drugREB      -0.14111    0.02638  -5.349 8.83e-08 ***
## visitvisit 2  0.09211    0.02622   3.514 0.000442 ***
## visitvisit 3  0.09638    0.02651   3.636 0.000277 ***
## genderfemale  0.12264    0.21137   0.580 0.561767
## ---
## Signif. codes:  0 '***' 0.001 '**' 0.01 '*' 0.05 '.' 0.1 ' ' 1
##
## Correlation of Fixed Effects:
##             (Intr) drgMPH drgREB vstvs2 vstvs3
## drugMPH     -0.102
## drugREB     -0.089  0.485
## visitvisit2 -0.101  0.103  0.072
## visitvisit3 -0.105  0.171  0.061  0.529
## genderfemal -0.699  0.000  0.000  0.000 -0.001
```

```r
report_glmer_effects(model_performance2)
```

```
##            Term Estimate_log     SE_log Estimate_exp    CI_lower    CI_upper
## 1  (Intercept)   4.91188124 0.15127589  135.8948252 101.0262606 182.7980507
## 2      drugMPH   0.03048602 0.02559385    1.0309555   0.9805144   1.0839914
## 3      drugREB  -0.14110878 0.02637848    0.8683948   0.8246381   0.9144733
## 4 visitvisit 2   0.09210877 0.02621565    1.0964841   1.0415668   1.1542969
## 5 visitvisit 3   0.09637847 0.02650822    1.1011757   1.0454238   1.1599009
## 6 genderfemale   0.12264075 0.21136987    1.1304782   0.7470343   1.7107394
##         p_value
## 1 2.856967e-231
## 2  2.335962e-01
## 3  8.825144e-08
## 4  4.422391e-04
## 5  2.771237e-04
## 6  5.617671e-01
```

```r
anova(model_performance1, model_performance2) # model 2 is better so we stick with this!
```

```
## Data: data_perf
## Models:
## model_performance1: rep_nr ~ drug + (1 | record_id)
## model_performance2: rep_nr ~ drug + visit + gender + (1 | record_id)
##                    npar    AIC    BIC  logLik deviance  Chisq Df Pr(>Chisq)
## model_performance1    4 749.71 757.66 -370.85   741.71
## model_performance2    7 738.47 752.39 -362.24   724.47 17.236  3   0.000632 ***
## ---
## Signif. codes:  0 '***' 0.001 '**' 0.01 '*' 0.05 '.' 0.1 ' ' 1
```

```r
# ------------------------------------------------------------ASSUMPTION CHECK

# WE USED THE DHARMA PACKAGE TO CHECK MODEL ASSUMTIONS

# Simulate residuals
sim_res <- simulateResiduals(model_performance2)
 # Plot diagnostics
plot(sim_res) # no significant problems detected
```

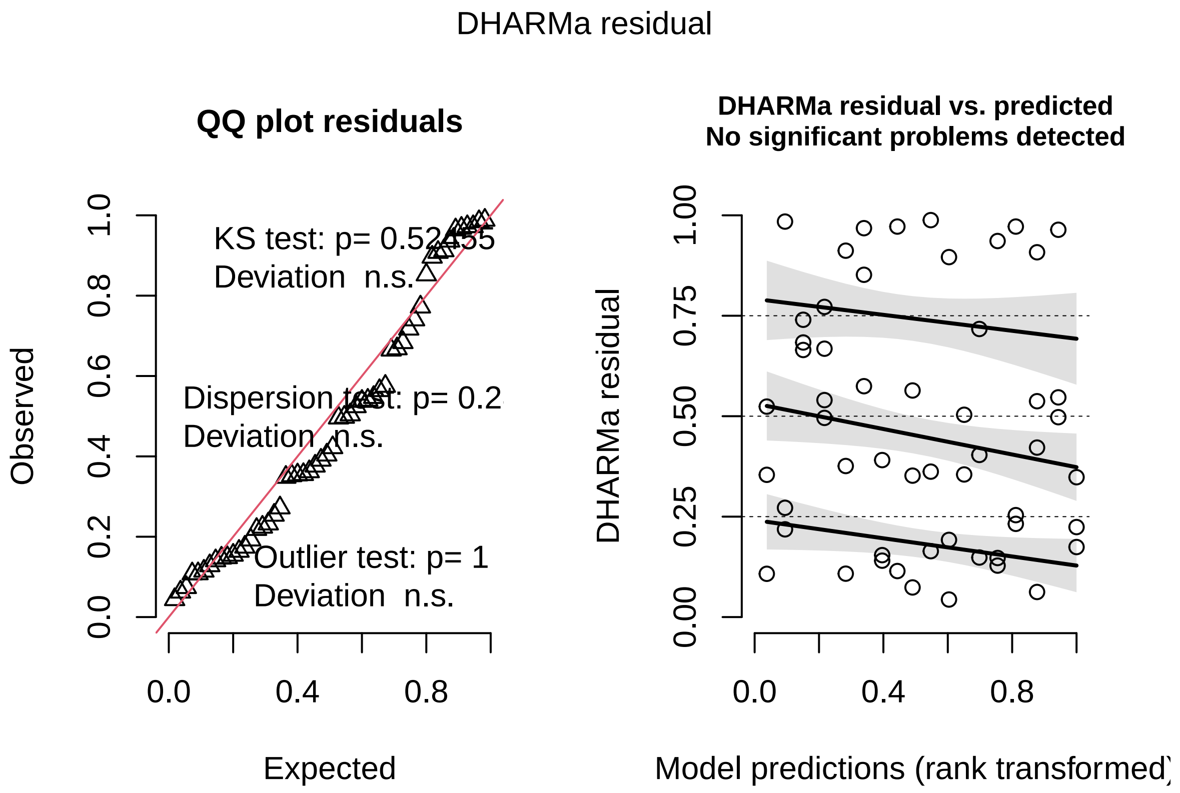


```
testDispersion(sim_res) # non-significant. no warnings
```

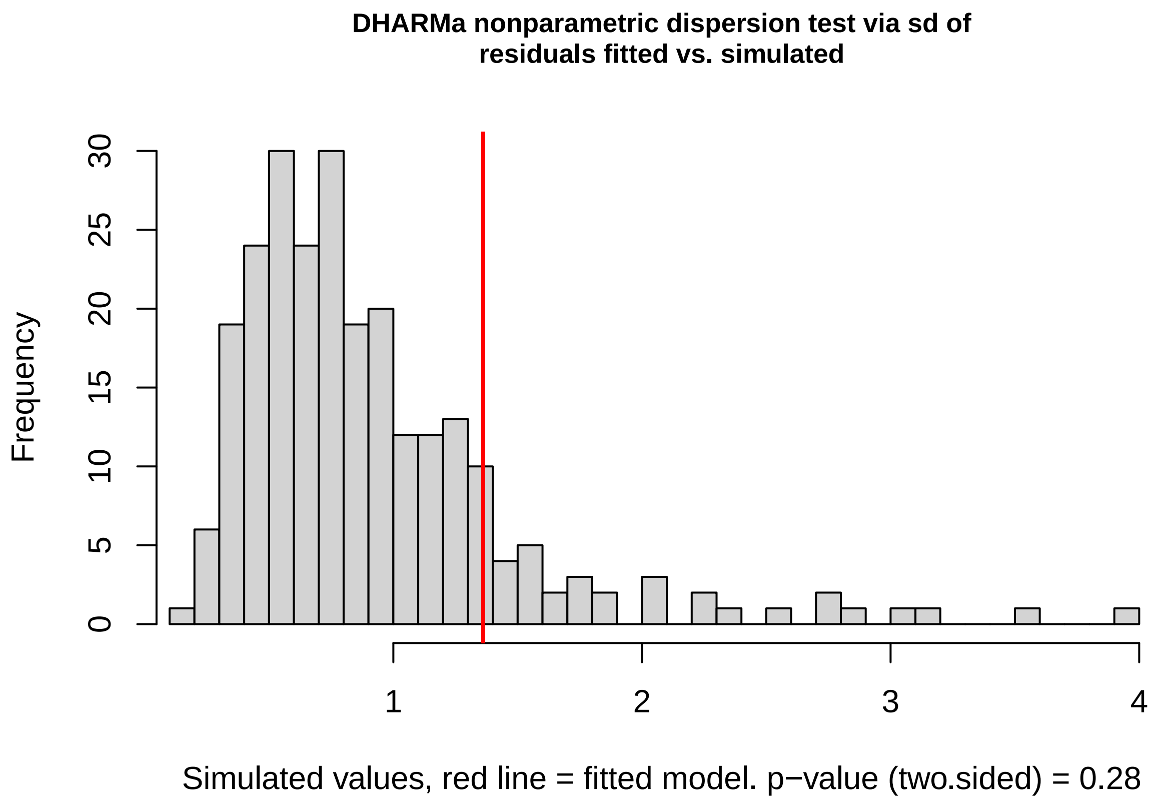


```
##
##  DHARMa nonparametric dispersion test via sd of residuals fitted vs.
##  simulated
##
## data:  simulationOutput
## dispersion = 1.4861, p-value = 0.28
## alternative hypothesis: two.sided
```

```
#---------------------------------------------------------------------------PLOTTING

# simple plot:
preds_performance2 <- ggpredict(model_performance2, terms = c("drug"))
```

```
## You are calculating adjusted predictions on the population-level (i.e.
##   `type = "fixed"`) for a *generalized* linear mixed model.
##   This may produce biased estimates due to Jensen's inequality. Consider
##   setting `bias_correction = TRUE` to correct for this bias.
##   See also the documentation of the `bias_correction` argument.
```

```
summary(preds_performance2)
```

```
##    x        predicted        std.error          conf.low          conf.high
##  PLA:1   Min.   :118.0   Min.   :0.1508   Min.   : 87.74   Min.   :158.7
##  MPH:1   1st Qu.:127.0   1st Qu.:0.1510   1st Qu.: 94.38   1st Qu.:170.8
```

```
##   REB:1    Median :135.9   Median :0.1512   Median :101.03   Median :182.8
##            Mean   :131.3   Mean   :0.1511   Mean   : 97.67   Mean   :176.6
##            3rd Qu.:138.0   3rd Qu.:0.1512   3rd Qu.:102.64   3rd Qu.:185.5
##            Max.   :140.1   Max.   :0.1513   Max.   :104.24   Max.   :188.3
##   group
##   1:3
##
##
##
##
##
```

```
plot(preds_performance2)
```

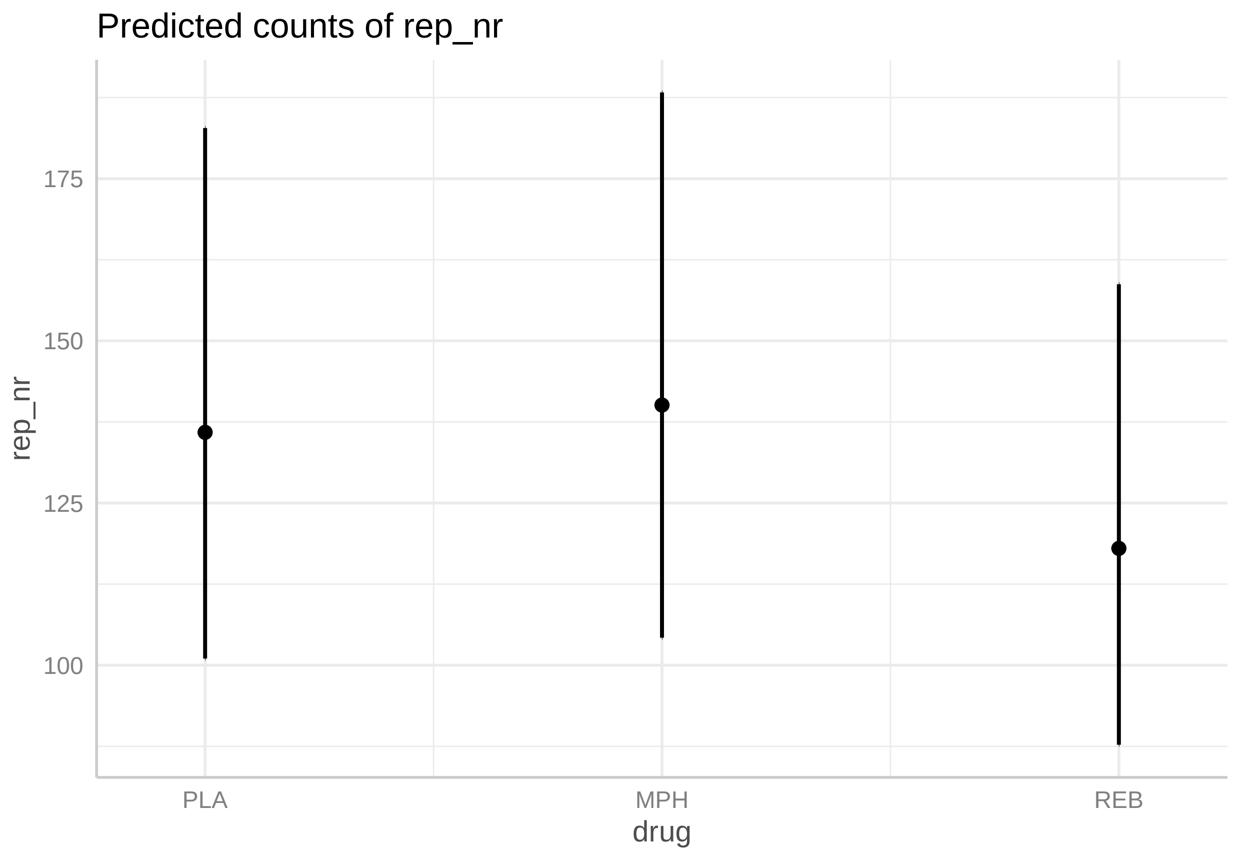


```
#----------------------------------------------------------------------PLOTS for publication

# Plot 2: Model predictions
# Define height for the significance bar
max_y <- max(preds_performance2$conf.high) + 10
bracket_height <- 5

p2 <- ggplot(preds_performance2, aes(x = x, y = predicted, color = x, shape = x)) +
  geom_point(size = 6) +
  geom_errorbar(aes(ymin = conf.low, ymax = conf.high), width = 0.1, size = 1) +
```

```r
  # Bracket horizontal line
  geom_segment(aes(x = 1, xend = 3, y = max_y, yend = max_y), color = "black", size = 0.8) +
  # Left vertical tick
  geom_segment(aes(x = 1, xend = 1, y = max_y, yend = max_y - bracket_height), color = "black", size = 
  # Right vertical tick
  geom_segment(aes(x = 3, xend = 3, y = max_y, yend = max_y - bracket_height), color = "black", size = 

  # Stars for significance
  annotate("text", x = 2, y = max_y + 5, label = "**", size = 6) +

  scale_color_brewer(palette = "Set2") +
  scale_shape_manual(values = c(17, 16, 15)) +
  scale_x_discrete(limits = c("PLA", "MPH", "REB")) +

  labs(
    x = "Drug conditions",
    y = "Predicted Performance (number of reps)",
    color = NULL,
    shape = NULL
  ) +
  theme_minimal(base_size = 16) +
  theme(
    axis.title.x = element_text(face = "bold", size = 22),
    axis.title.y = element_text(face = "bold", size = 20),
    axis.text.x = element_blank(),
    axis.ticks.x = element_blank(),
    axis.text.y = element_text(color = "black", size = 22),
    legend.position = "right",              # keep legend visible
    legend.text = element_text(size = 18),
    legend.key.size = unit(2, "lines"),
    panel.grid.major = element_line(size = 0.5, linetype = "solid", color = "gray90"),
    panel.grid.minor = element_line(size = 0.25, linetype = "dotted", color = "gray90"),
    axis.line = element_line(size = 0.8, color = "black"),
    plot.margin = margin(10, 10, 10, 10)
  )
```

```
## Warning: Using `size` aesthetic for lines was deprecated in ggplot2 3.4.0.
## i Please use `linewidth` instead.
## This warning is displayed once every 8 hours.
## Call `lifecycle::last_lifecycle_warnings()` to see where this warning was
## generated.
```

```
## Warning: The `size` argument of `element_line()` is deprecated as of ggplot2 3.4.0.
## i Please use the `linewidth` argument instead.
## This warning is displayed once every 8 hours.
## Call `lifecycle::last_lifecycle_warnings()` to see where this warning was
## generated.
```

```r
p2
```

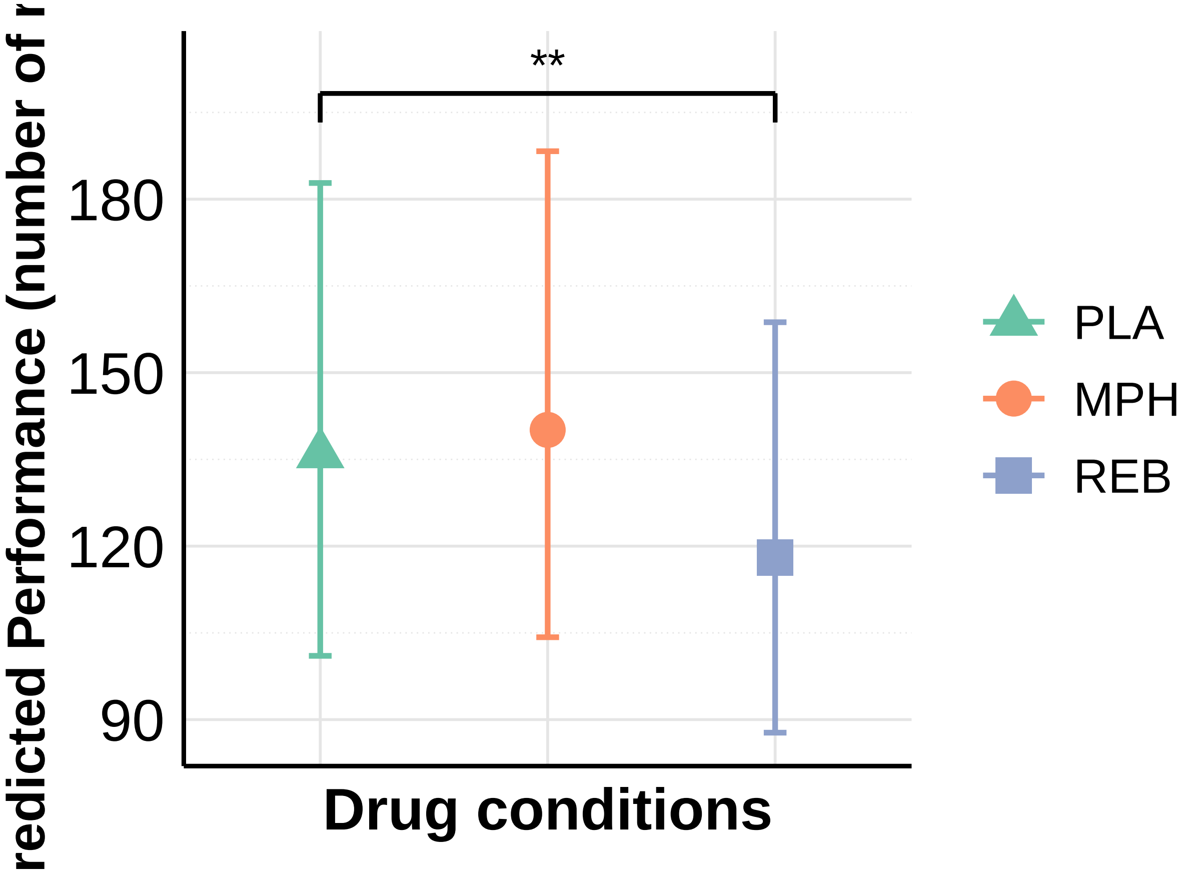


```r
# Plot 1: Raw data violin + boxplot
# Define a shared jitter position

p1 <- ggplot(data_perf, aes(x = drug, y = rep_nr, color = drug, fill = drug)) +
  geom_violin(trim = FALSE, alpha = 0.2, size = 0.6) +
  geom_boxplot(width = 0.25, outlier.shape = NA, fill = "white", size = 1) +
  geom_jitter(alpha = 0.4, color = "black", size = 1.2, width = 0.0001) +
  geom_line(aes(group = record_id), color = "gray70", alpha = 0.5) +
  scale_color_brewer(palette = "Set2") +
  scale_fill_brewer(palette = "Set2") +
  labs(
    x = "Drug conditions",
    y = "Performance (number of reps)",
    color = NULL,
    fill = NULL
  ) +
  theme_minimal(base_size = 16) +
  theme(
    axis.title = element_text(face = "bold", size = 22),
    axis.text.x = element_blank(),         # hide x-axis labels
    axis.ticks.x = element_blank(),        # hide x-axis ticks
    axis.text.y = element_text(color = "black", size = 20),
    plot.title = element_text(face = "bold", size = 20, hjust = 0.5),
    panel.grid.major = element_line(size = 0.5, linetype = "solid", color = "gray90"),
    panel.grid.minor = element_line(size = 0.25, linetype = "dotted", color = "gray90"),
    axis.ticks = element_line(size = 0.6),
```

```r
    axis.line = element_line(size = 0.8, color = "black"),
    plot.margin = margin(10, 10, 10, 10),
    legend.position = "right",              # keep legend visible
    legend.text = element_text(size = 18),
    legend.key.size = unit(2, "lines")
  )

p1
```

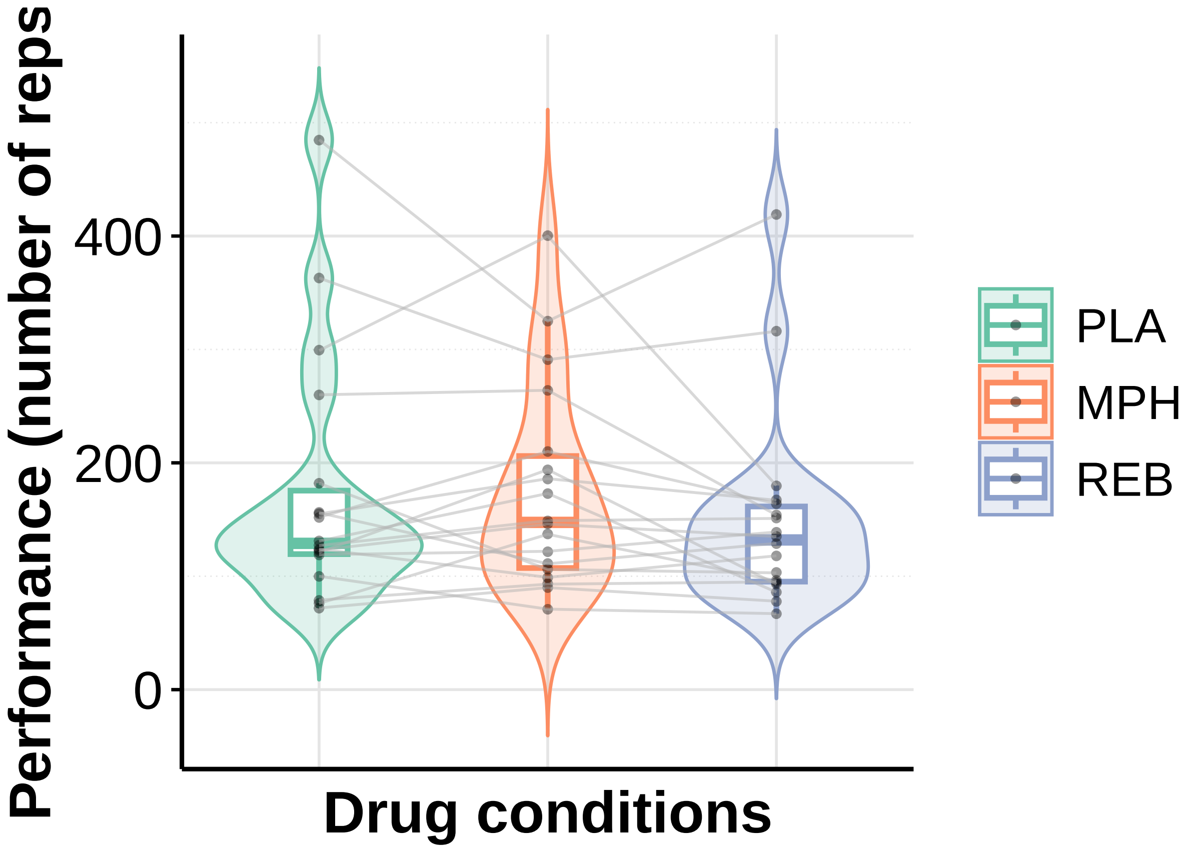


```r
# Combine side by side
p1 + plot_spacer() + p2 +
  plot_layout(ncol = 3, widths = c(1, 0.1, 1))
```

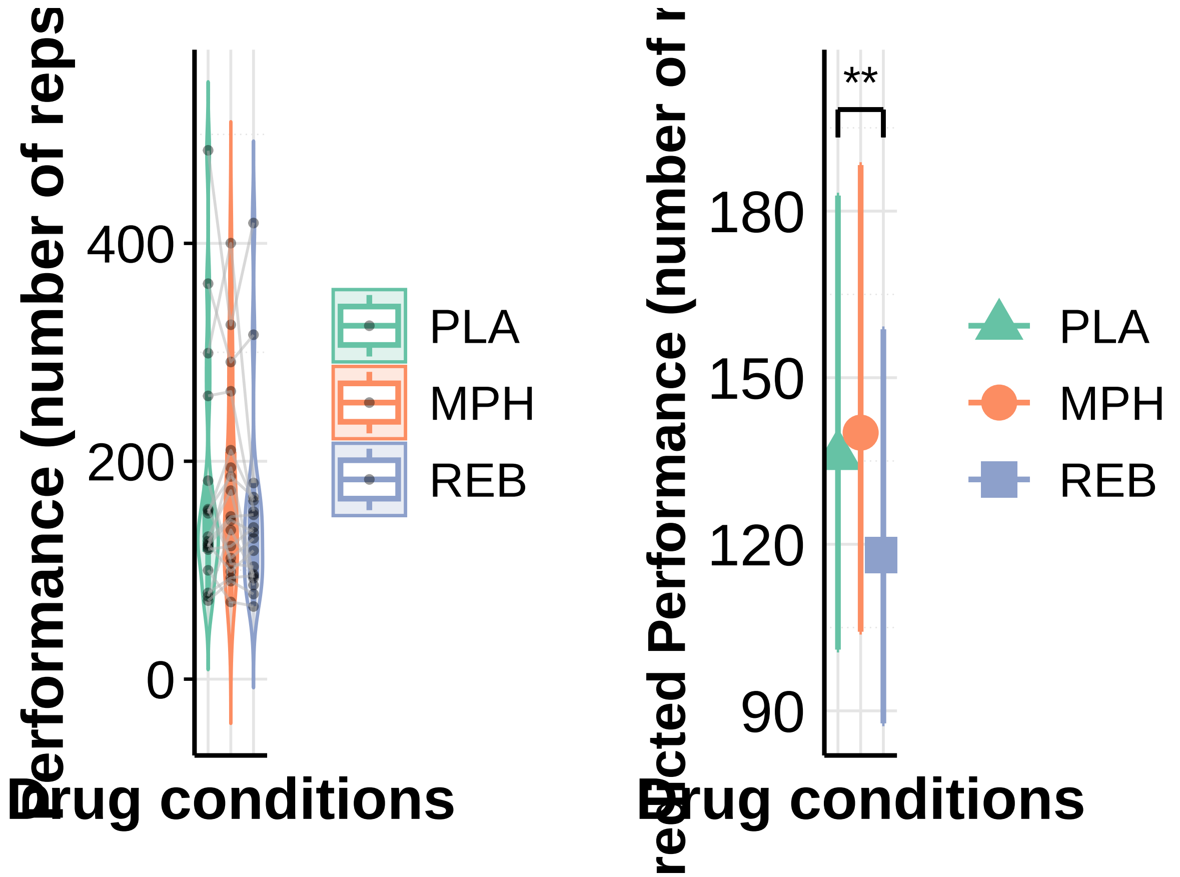


```r
ggsave("raw_predicted_performance.png", width = 19, height = 6, dpi = 600)
```

4.2 PERCEIVED FATIGUE AND EXERTION

4.21 DATA DESCRIPTIVES AND VISUALIZATION (PERCEIVED FATIGUE AND EXERTION)

```r
# -----------------------------------PVAS/RPE DESCRIPTIVES--------------------------------------------
# 5 values of RPE, PVAS percentage failure ( 0-0.25-0.5-75-100 %)

mean_data_pvas_rpe <- data_pvas_rpe %>%
  group_by(drug, percentile) %>%
  summarise(
    mean_pvas = round(mean(pvas, na.rm = TRUE), 2),
    sd_pvas = round(sd(pvas, na.rm = TRUE), 2),
    mean_oral_rpe = round(mean(oral_rpe, na.rm = TRUE), 2),
    sd_oral_rpe = round(sd(oral_rpe, na.rm = TRUE), 2)
  )
```

```
## `summarise()` has grouped output by 'drug'. You can override using the
## `.groups` argument.
```

```r
view(mean_data_pvas_rpe)
```

```r
#------------------------------------ Visualize by boxplot # PVAS

ggplot(data_pvas_rpe, aes(x = factor(percentile), y = pvas, fill = drug)) +
  geom_boxplot(position = position_dodge(width = 0.8), outlier.shape = NA) +  # Hide "outliers"
  geom_jitter(aes(color = drug),
              position = position_jitterdodge(jitter.width = 0.2, dodge.width = 0.8),
              size = 1.5, alpha = 0.6) +  # Show raw points
  stat_summary(fun = mean, geom = "point",
               position = position_dodge(width = 0.8),
               size = 3, shape = 18, aes(color = drug)) +
  labs(title = "pVAS by Time (Percentile) and Drug",
       x = "Time (Percentile)", y = "pVAS") +
  theme_minimal() +
  scale_fill_brewer(palette = "Set2") +
  scale_color_brewer(palette = "Set2")
```

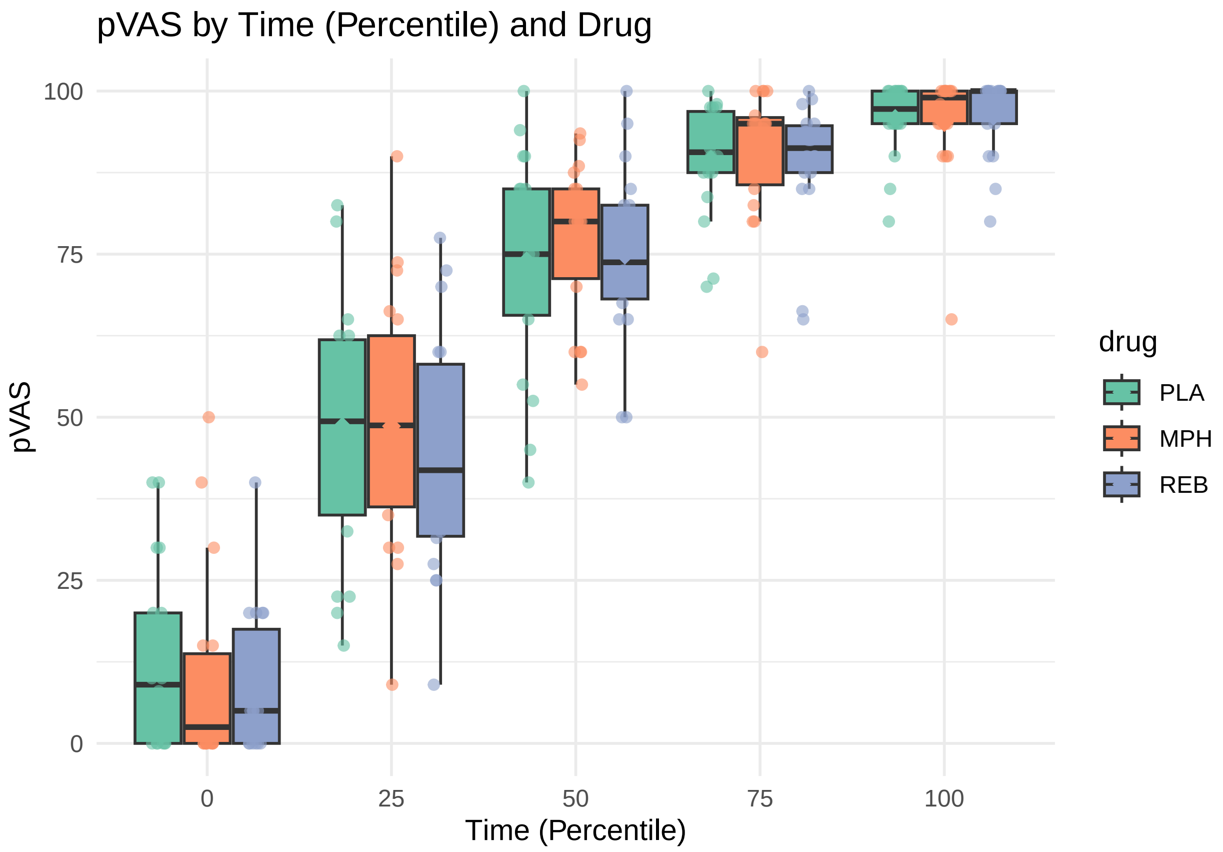


```r
# Visualize by boxplot # ORAL RPE

ggplot(data_pvas_rpe, aes(x = factor(percentile), y = oral_rpe, fill = drug)) +
  geom_boxplot(position = position_dodge(width = 0.8), outlier.shape = NA) +  # Hide outliers
  geom_jitter(aes(color = drug),
              position = position_jitterdodge(jitter.width = 0.2, dodge.width = 0.8),
              size = 1.5, alpha = 0.6) +  # Show raw points
```

```r
  stat_summary(fun = mean, geom = "point",
               position = position_dodge(width = 0.8),
               size = 3, shape = 18, aes(color = drug)) +
  labs(title = "Oral RPE by Time (Percentile) and Drug",
       x = "Time (Percentile)", y = "Oral RPE") +
  theme_minimal() +
  scale_fill_brewer(palette = "Set2") +
  scale_color_brewer(palette = "Set2")
```

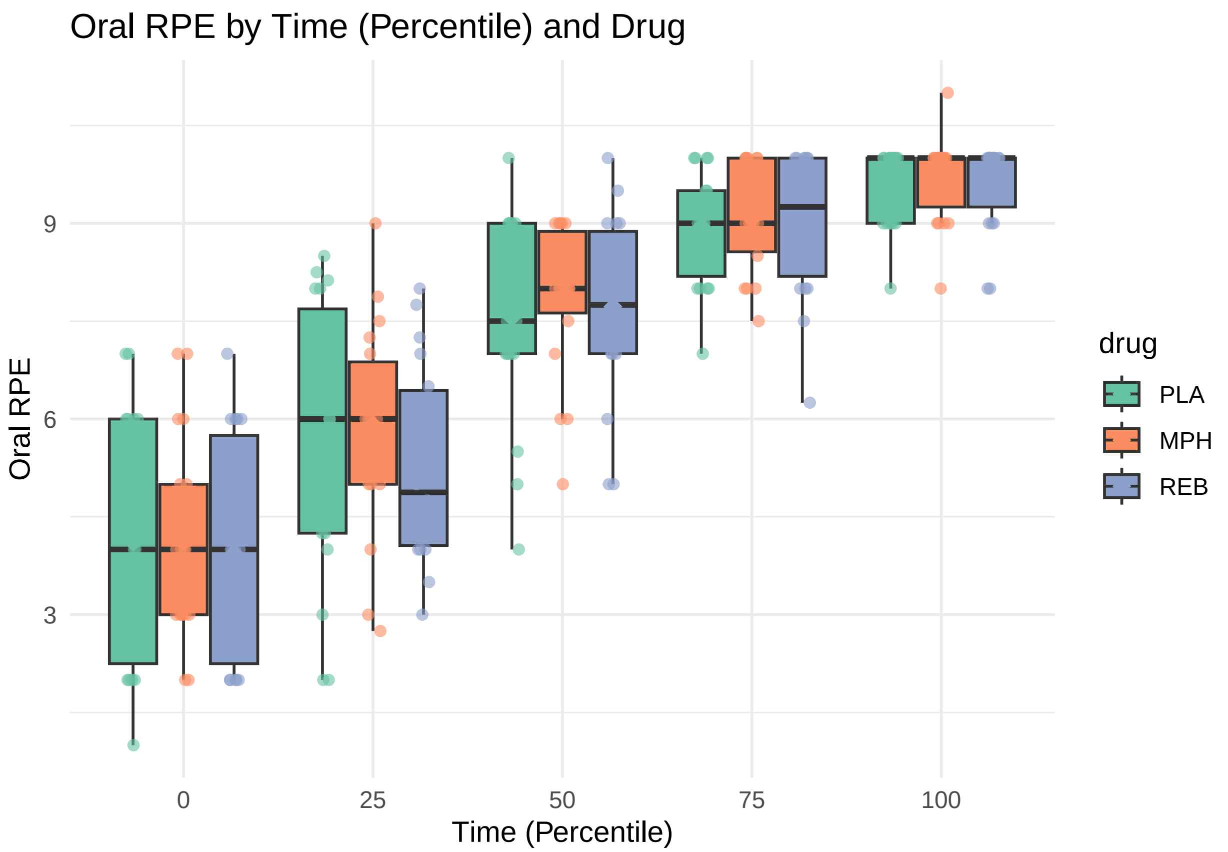


```r
# HISTOGRAM
ggplot(data_pvas_rpe, aes(x = pvas, fill = drug)) +
  geom_histogram(binwidth = 5, position = "dodge", color = "black", alpha = 0.7) +
  facet_wrap(~ percentile, ncol = 3) +
  labs(title = "Distribution of pVAS by Percentile and Drug",
       x = "pVAS Score", y = "Count") +
  theme_minimal() +
  scale_fill_brewer(palette = "Set2")
```

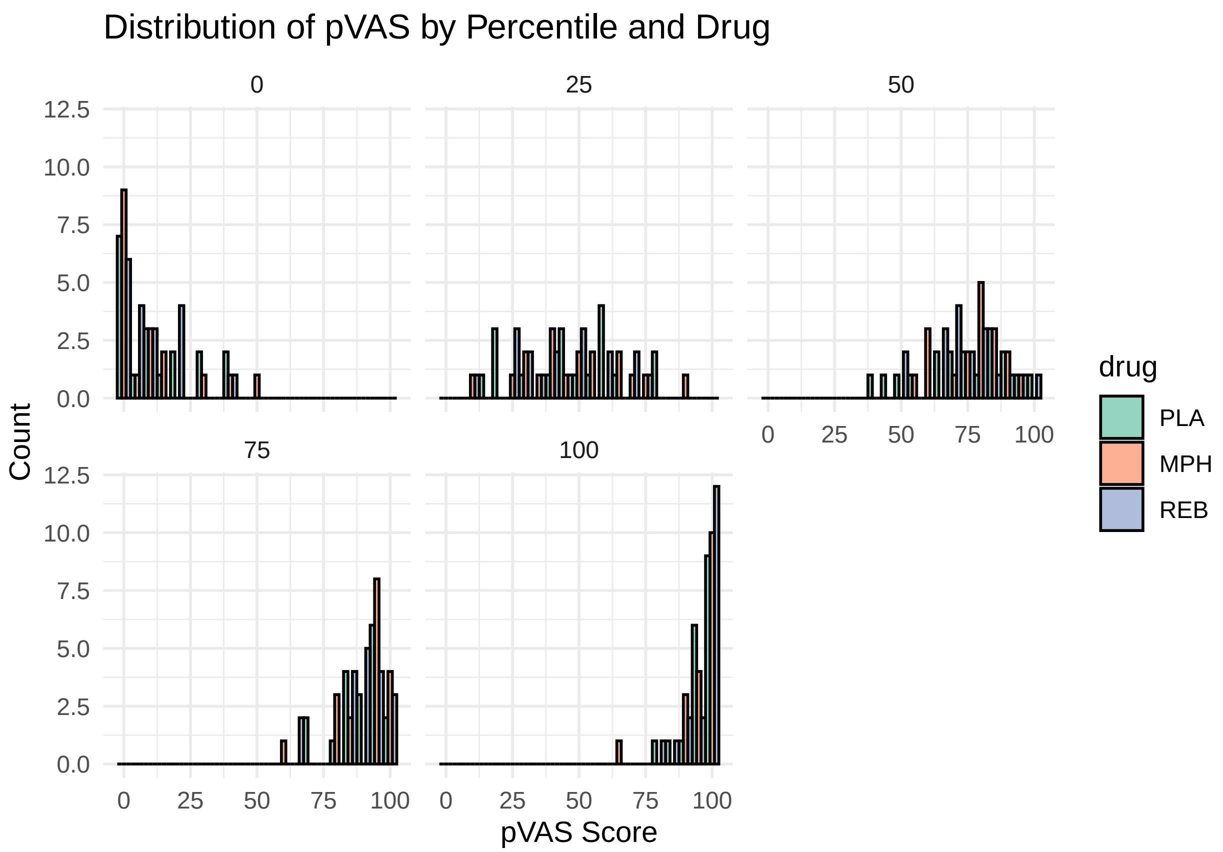


```r
ggplot(data_pvas_rpe, aes(x = oral_rpe, fill = drug)) +
  geom_histogram(binwidth = 1, position = "dodge", color = "black", alpha = 0.7) +
  facet_wrap(~ percentile, ncol = 3) +
  labs(title = "Distribution of Oral RPE by Percentile and Drug",
       x = "Oral RPE Score", y = "Count") +
  theme_minimal() +
  scale_fill_brewer(palette = "Set2")
```

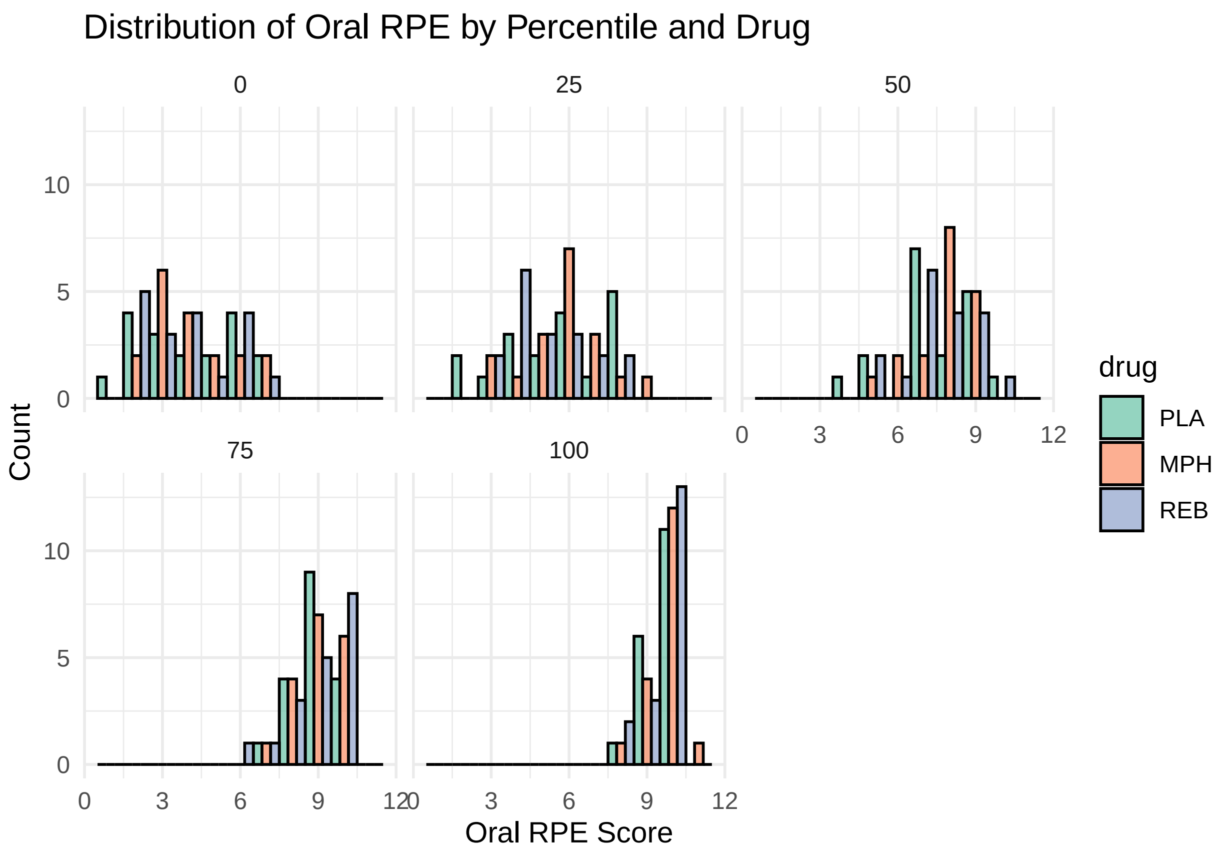

4.22 DATA ANALYSES (PERCEIVED FATIGUE AND EXERTION)

```
#-------------------------------------------------------------------------PVAS MODEL
# Fit ART model with interaction and random effect for repeated measures

art_model <- art(pvas ~ drug*percentile, data = data_pvas_rpe)

# Test main effects and interaction
anova(art_model)
```

```
## Analysis of Variance of Aligned Rank Transformed Data
##
## Table Type: Anova Table (Type III tests)
## Model: No Repeated Measures (lm)
## Response: art(pvas)
##
##                     Df Df.res   F value  Pr(>F)
## 1 drug               2    255   0.81189 0.44516
## 2 percentile         4    255 275.80363 < 2e-16 ***
## 3 drug:percentile    8    255   0.53707 0.82806
## ---
## Signif. codes:   0 '***' 0.001 '**' 0.01 '*' 0.05 '.' 0.1 ' ' 1
```

```r
emmeans_percentile <- emmeans(artlm(art_model, "percentile"), pairwise ~ percentile)
```

```
## NOTE: Results may be misleading due to involvement in interactions
```

```r
summary(emmeans_percentile, adjust = "Bonferroni")
```

```
## $emmeans
##  percentile emmean   SE  df lower.CL upper.CL
##  0            31.0 4.73 255     18.7     43.2
##  25           86.2 4.73 255     74.0     98.5
##  50          143.4 4.73 255    131.1    155.7
##  75          191.5 4.73 255    179.2    203.7
##  100         225.4 4.73 255    213.2    237.7
##
## Results are averaged over the levels of: drug
## Confidence level used: 0.95
## Conf-level adjustment: bonferroni method for 5 estimates
##
## $contrasts
##  contrast                     estimate   SE  df t.ratio p.value
##  percentile0 - percentile25      -55.3 6.68 255  -8.271  <.0001
##  percentile0 - percentile50     -112.4 6.68 255 -16.824  <.0001
##  percentile0 - percentile75     -160.5 6.68 255 -24.014  <.0001
##  percentile0 - percentile100    -194.5 6.68 255 -29.100  <.0001
##  percentile25 - percentile50     -57.2 6.68 255  -8.552  <.0001
##  percentile25 - percentile75    -105.2 6.68 255 -15.743  <.0001
##  percentile25 - percentile100   -139.2 6.68 255 -20.829  <.0001
##  percentile50 - percentile75     -48.1 6.68 255  -7.190  <.0001
##  percentile50 - percentile100    -82.0 6.68 255 -12.276  <.0001
##  percentile75 - percentile100    -34.0 6.68 255  -5.086  <.0001
##
## Results are averaged over the levels of: drug
## P value adjustment: bonferroni method for 10 tests
```

```r
#-------------------------------------------------------------------------RPE MODEL
# Fit ART model with interaction and random effect for repeated measures

art_model2 <- art(oral_rpe ~ drug*percentile, data = data_pvas_rpe)

# Test main effects and interaction
anova(art_model2)
```

```
## Analysis of Variance of Aligned Rank Transformed Data
##
## Table Type: Anova Table (Type III tests)
## Model: No Repeated Measures (lm)
## Response: art(oral_rpe)
##
##                   Df Df.res   F value  Pr(>F)
## 1 drug             2    255   0.65322 0.52124
## 2 percentile       4    255 179.12487 < 2e-16 ***
## 3 drug:percentile  8    255   0.39775 0.92123
```

```
## ---
## Signif. codes:   0 '***' 0.001 '**' 0.01 '*' 0.05 '.' 0.1 ' ' 1
```

```
emmeans_percentile <- emmeans(artlm(art_model2, "percentile"), pairwise ~ percentile)
```

```
## NOTE: Results may be misleading due to involvement in interactions
```

```
summary(emmeans_percentile, adjust = "Bonferroni")
```

```
## $emmeans
##  percentile emmean   SE  df lower.CL upper.CL
##  0            44.5 5.57 255     30.0     59.0
##  25           79.7 5.57 255     65.2     94.1
##  50          138.7 5.57 255    124.2    153.1
##  75          191.0 5.57 255    176.5    205.4
##  100         223.7 5.57 255    209.2    238.2
##
## Results are averaged over the levels of: drug
## Confidence level used: 0.95
## Conf-level adjustment: bonferroni method for 5 estimates
##
## $contrasts
##  contrast                     estimate   SE  df t.ratio p.value
##  percentile0 - percentile25      -35.2 7.88 255  -4.461  0.0001
##  percentile0 - percentile50      -94.2 7.88 255 -11.945  <.0001
##  percentile0 - percentile75     -146.5 7.88 255 -18.582  <.0001
##  percentile0 - percentile100    -179.2 7.88 255 -22.730  <.0001
##  percentile25 - percentile50     -59.0 7.88 255  -7.484  <.0001
##  percentile25 - percentile75    -111.3 7.88 255 -14.121  <.0001
##  percentile25 - percentile100   -144.0 7.88 255 -18.269  <.0001
##  percentile50 - percentile75     -52.3 7.88 255  -6.636  <.0001
##  percentile50 - percentile100    -85.0 7.88 255 -10.785  <.0001
##  percentile75 - percentile100    -32.7 7.88 255  -4.149  0.0005
##
## Results are averaged over the levels of: drug
## P value adjustment: bonferroni method for 10 tests
```

```
# -------------------------------------------------------------------------PLOT PVAS
# general plot
summary_df <- data_pvas_rpe %>%
  group_by(drug, percentile) %>%
  summarise(
    mean_pvas = mean(pvas, na.rm = TRUE),
    sd_pvas = sd(pvas, na.rm = TRUE),
    n = n(),
    se = sd_pvas / sqrt(n),
    lower = mean_pvas - 1.96 * se,
    upper = mean_pvas + 1.96 * se
  ) %>%
  ungroup()
```

```
## `summarise()` has grouped output by 'drug'. You can override using the
## `.groups` argument.
```

```r
summary_df2 <- data_pvas_rpe %>%
  group_by(drug, percentile) %>%
  summarise(
    mean_rpe = mean(oral_rpe, na.rm = TRUE),
    sd_rpe = sd(oral_rpe, na.rm = TRUE),
    n = n(),
    se = sd_rpe / sqrt(n),
    lower = mean_rpe - 1.96 * se,
    upper = mean_rpe + 1.96 * se
  ) %>%
  ungroup()
```

```
## `summarise()` has grouped output by 'drug'. You can override using the
## `.groups` argument.
```

```r
# PLOT for submission!


# Define bracket for time difference from 0 to 100
bracket_x_start <- 1
bracket_x_end <- 5
bracket_y <- max(summary_df$mean_pvas + summary_df$se, na.rm = TRUE) + 5

ggplot(summary_df, aes(x = percentile, y = mean_pvas, color = drug, group = drug,
                       linetype = drug, shape = drug)) +
  geom_point(size = 3.5) +
  geom_line(size = 0.71) +
  geom_errorbar(aes(ymin = lower, ymax = upper), width = 0.15, size = 1) +
  scale_color_brewer(palette = "Dark2") +
  scale_linetype_manual(values = c("solid", "dashed", "dotdash")) +
  scale_shape_manual(values = c(16, 17, 15)) +  # circle, triangle, square
  labs(
    x = "Percentile",
    y = "Perceived fatigue (0-100)",
    color = "Drug",
    linetype = "Drug",
    shape = "Drug"
  ) +
  theme_minimal(base_size = 14) +
  theme(
    legend.position = "right",
    axis.title = element_text(face = "bold", size = 16),
    axis.text = element_text(color = "black", size = 14)
  ) +
  # Add bracket line and asterisk
  geom_segment(aes(x = bracket_x_start, xend = bracket_x_end,
                   y = bracket_y, yend = bracket_y), color = "black", size = 0.8) +
  annotate("text", x = (bracket_x_start + bracket_x_end) / 2,
           y = bracket_y + 2, label = "**", size = 6)
```

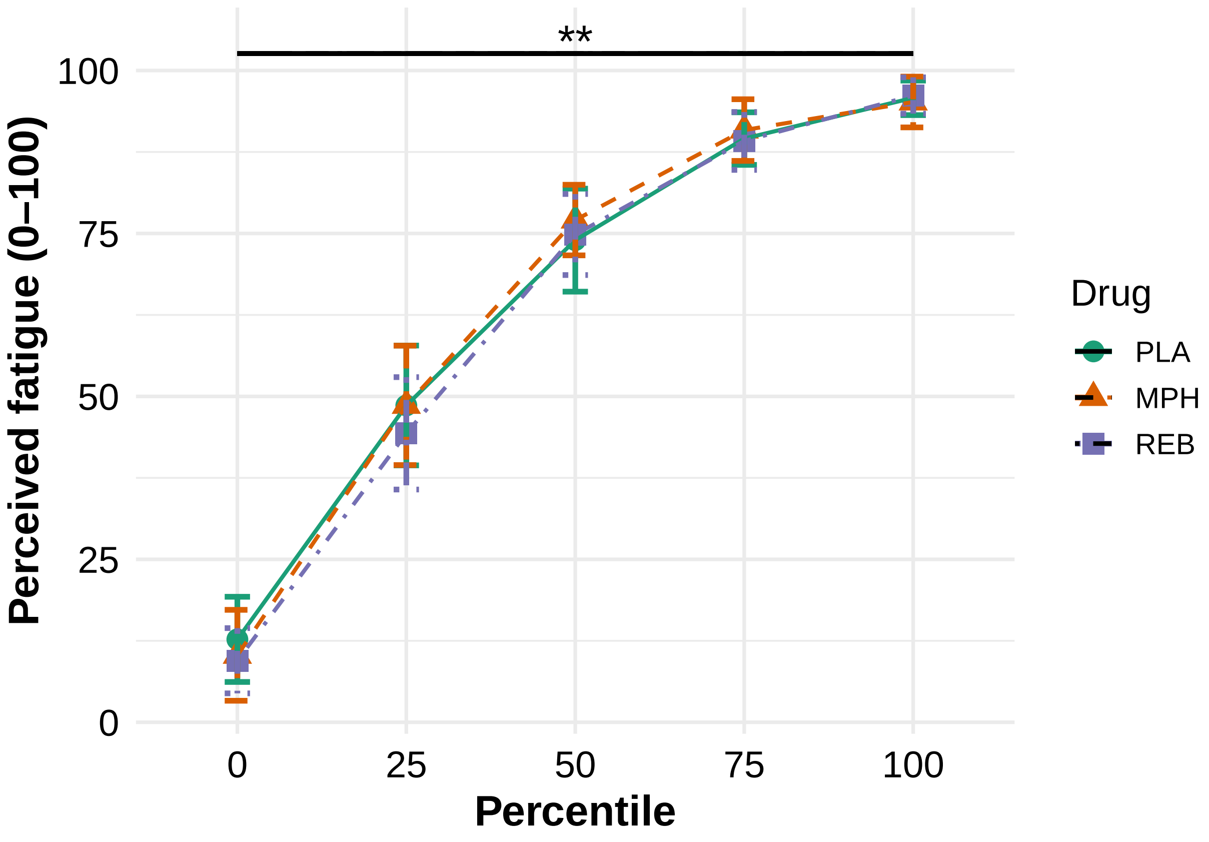


```
# -------------------------------------------------------------------------PLOT RPE
summary_df2 <- data_pvas_rpe %>%
  group_by(drug, percentile) %>%
  summarise(
    mean_rpe = mean(oral_rpe, na.rm = TRUE),
    sd_rpe = sd(oral_rpe, na.rm = TRUE),
    n = n(),
    se = sd_rpe / sqrt(n),
    lower = mean_rpe - 1.96 * se,
    upper = mean_rpe + 1.96 * se
  ) %>%
  ungroup()
```

```
## 'summarise()' has grouped output by 'drug'. You can override using the
## '.groups' argument.
```

```
# PLOT for submussion!
# Define bracket position for RPE plot
bracket_x_start <- 1
bracket_x_end <- 5
bracket_y <- max(summary_df2$mean_rpe + summary_df2$se, na.rm = TRUE) + 0.5  # adjust height if needed

ggplot(summary_df2, aes(x = percentile, y = mean_rpe, color = drug, group = drug,
                        linetype = drug, shape = drug)) +
  geom_point(size = 3.5) +
```

```
geom_line(size = 0.71) +
geom_errorbar(aes(ymin = lower, ymax = upper), width = 0.15, size = 1) +
scale_color_brewer(palette = "Dark2") +
scale_linetype_manual(values = c("solid", "dashed", "dotdash")) +
scale_shape_manual(values = c(16, 17, 15)) +  # circle, triangle, square
labs(
  x = "Percentile",
  y = "RPE (0-10)",
  color = "Drug",
  linetype = "Drug",
  shape = "Drug"
) +
theme_minimal(base_size = 14) +
theme(
  legend.position = "right",
  axis.title = element_text(face = "bold", size = 16),
  axis.text = element_text(color = "black", size = 14)
) +
# Add bracket line and asterisk
geom_segment(aes(x = bracket_x_start, xend = bracket_x_end,
                 y = bracket_y, yend = bracket_y),
             color = "black", size = 0.8) +
annotate("text", x = (bracket_x_start + bracket_x_end) / 2,
         y = bracket_y + 0.2, label = "**", size = 6)
```

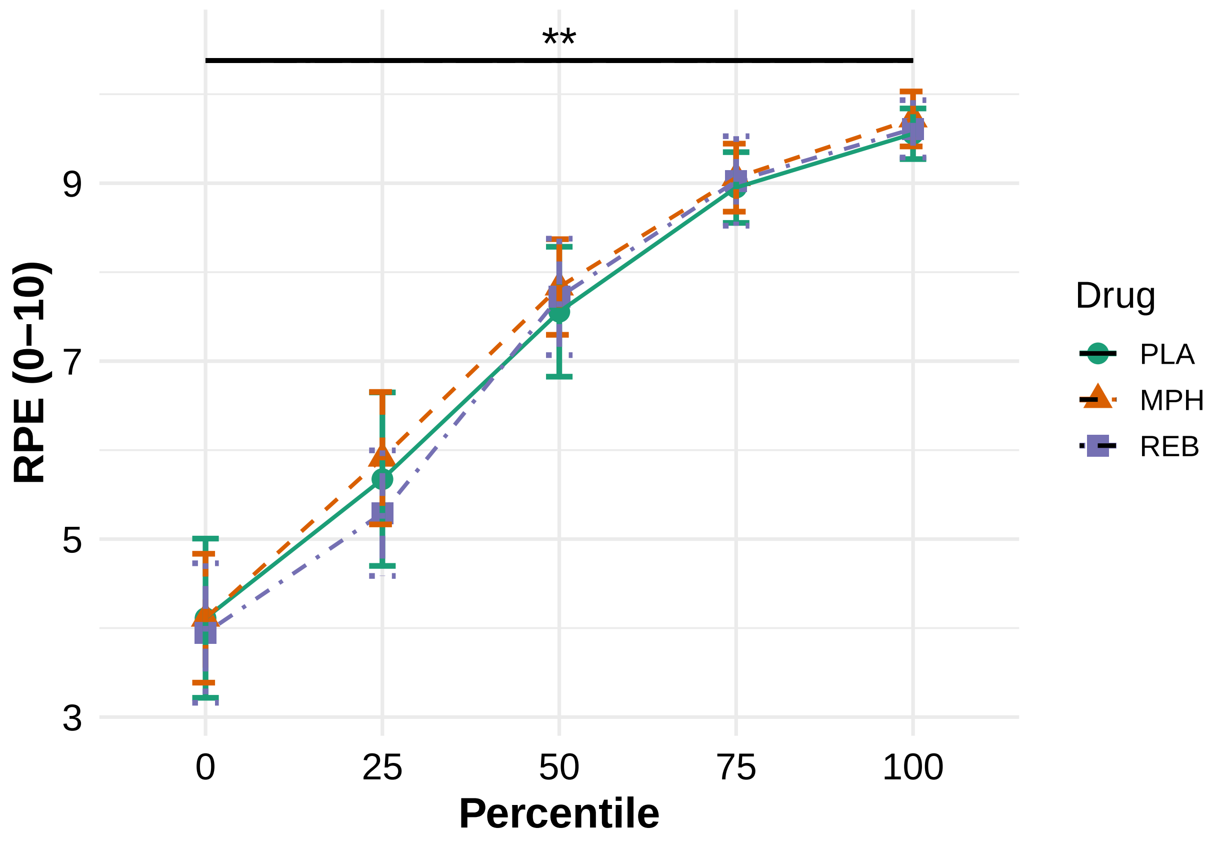

```r
# save plot!!!
ggsave("RPE.png", width = 8, height = 6, dpi = 600)
```

```r
#---------------------------------------------------PLOT TOGETHER PVAS and RPE



# Define bracket for PVAS plot
bracket_x_start <- 1
bracket_x_end <- 5
bracket_y1 <- max(summary_df$mean_pvas + summary_df$se, na.rm = TRUE) + 5

# PLOT 1: PVAS
p1 <- ggplot(summary_df, aes(x = percentile, y = mean_pvas, color = drug, group = drug,
                             linetype = drug, shape = drug)) +
  geom_point(size = 3.5) +
  geom_line(size = 0.71) +
  geom_errorbar(aes(ymin = lower, ymax = upper), width = 0.15, size = 1) +
  scale_color_brewer(palette = "Dark2") +
  scale_linetype_manual(values = c("solid", "dashed", "dotdash")) +
  scale_shape_manual(values = c(16, 17, 15)) +
  labs(
    x = "Percentage failure",
    y = "Perceived fatigue (0-100)",
    color = "Drug",
    linetype = "Drug",
    shape = "Drug"
  ) +
  theme_minimal(base_size = 14) +
  theme(
    axis.title = element_text(face = "bold", size = 20),
    axis.text = element_text(color = "black", size = 22)
  ) +
  geom_segment(aes(x = bracket_x_start, xend = bracket_x_end,
                   y = bracket_y1, yend = bracket_y1), color = "black", size = 0.8) +
  annotate("text", x = (bracket_x_start + bracket_x_end) / 2,
           y = bracket_y1 + 2, label = "**", size = 6)

# Define bracket for RPE plot
bracket_y2 <- max(summary_df2$mean_rpe + summary_df2$se, na.rm = TRUE) + 0.5

# PLOT 2: RPE
p2 <- ggplot(summary_df2, aes(x = percentile, y = mean_rpe, color = drug, group = drug,
                              linetype = drug, shape = drug)) +
  geom_point(size = 3.5) +
  geom_line(size = 0.71) +
  geom_errorbar(aes(ymin = lower, ymax = upper), width = 0.15, size = 1) +
  scale_color_brewer(palette = "Dark2") +
  scale_linetype_manual(values = c("solid", "dashed", "dotdash")) +
  scale_shape_manual(values = c(16, 17, 15)) +
  labs(
    x = "Percentage failure",
    y = "RPE (0-10)",
```

```r
    color = "Drug",
    linetype = "Drug",
    shape = "Drug"
  ) +
  theme_minimal(base_size = 14) +
  theme(
    axis.title = element_text(face = "bold", size = 20),
    axis.text = element_text(color = "black", size = 22)
  ) +
  geom_segment(aes(x = bracket_x_start, xend = bracket_x_end,
                   y = bracket_y2, yend = bracket_y2), color = "black", size = 0.8) +
  annotate("text", x = (bracket_x_start + bracket_x_end) / 2,
           y = bracket_y2 + 0.2, label = "**", size = 6)

# Combine plots with shared legend
combined_plot <- p1 + p2 +
  plot_layout(ncol = 2, guides = "collect") &
  theme(
    legend.position = "bottom",
    legend.text = element_text(size = 22),
    legend.title = element_text(size = 24, face = "bold")
  )


# Show the plot
print(combined_plot)
```

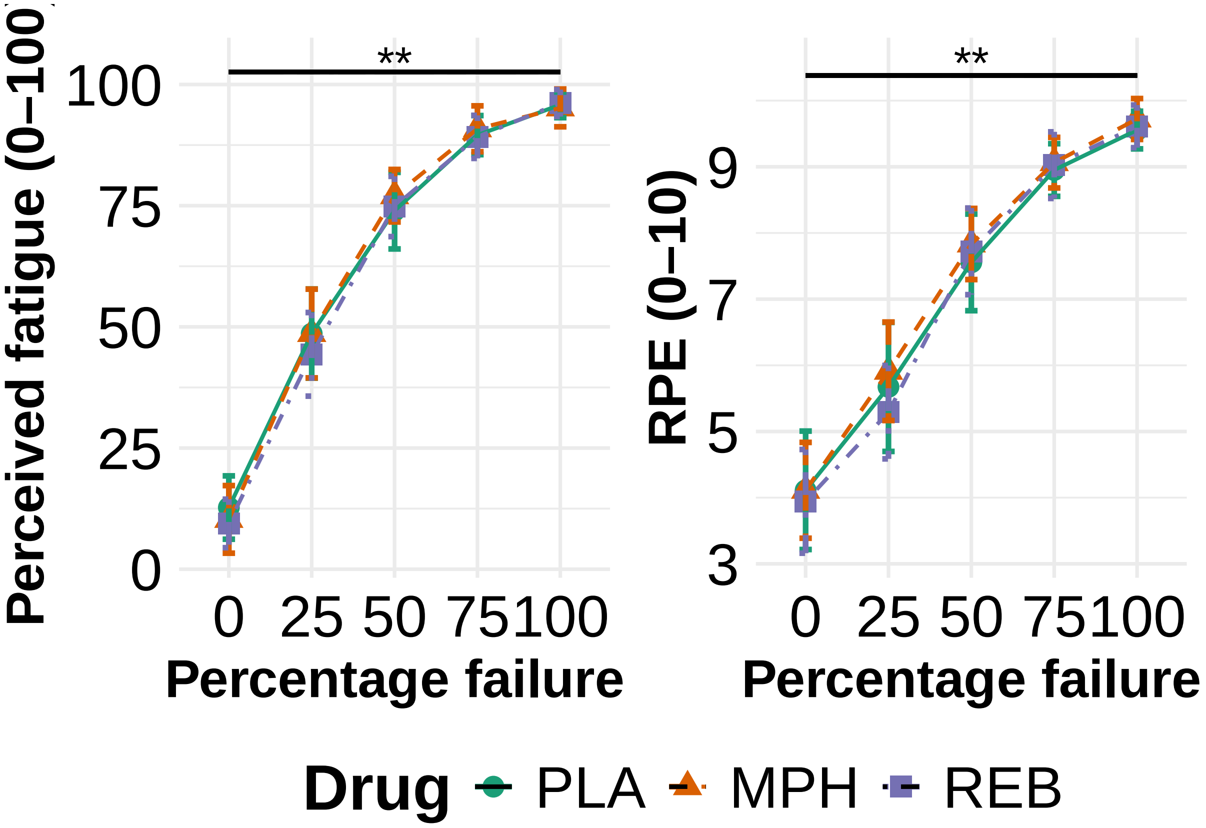


```
# Save the combined plot
ggsave("combined_plot_large.png", combined_plot, width = 18, height = 8, dpi = 600)
```

4.3 MOTIVATION

4.31 DATA DESCRIPTIVES AND VISUALIZATION (MOTIVATION)

```
# ----------------------------------------MOTIVATION --------------------------------------------------
# one value pre fatigue

# Compute descriptives
mean_data_motivation <- data_perf %>%
  group_by(drug) %>%
  summarise(
    mean = round(mean(motivation, na.rm = TRUE), 2),
    sd = round(sd(motivation, na.rm = TRUE), 2)
  )
view(mean_data_motivation)

# Visualize by boxplot
ggplot(data_perf, aes(x = drug, y = motivation)) +
    geom_boxplot(position = position_dodge(width = 0.8), outlier.shape = NA) +  # Hide "outliers"
  geom_jitter(aes(color = drug),
              position = position_jitterdodge(jitter.width = 0.2, dodge.width = 0.8),
```

```r
             size = 1.5, alpha = 0.6) +  # Show raw points
  geom_boxplot(fill = "white", color = "darkgreen") +
  geom_point(data = mean_data_motivation, aes(x = drug, y = mean),
             color = "red", size = 3, shape = 18) +  # Red diamonds for means
  labs(title = "motivation per Drug with Group Means",
       x = "Drug", y = "motivation") +
  theme_minimal()
```

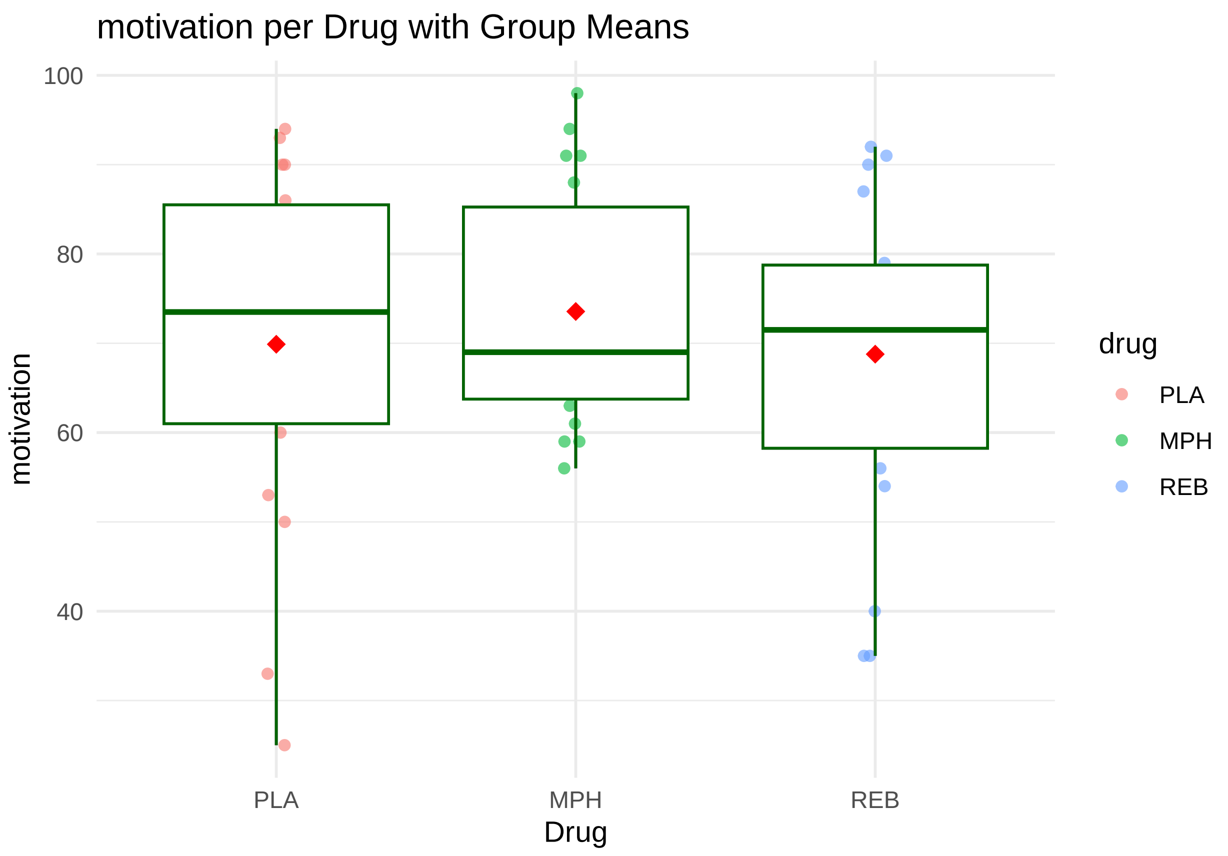


```r
# Histogram with adjusted binwidth for rep_nr range
ggplot(data_perf, aes(x = motivation, fill = drug)) +
  geom_histogram(binwidth = 5, position = "dodge", color = "black", alpha = 0.7) +
  labs(title = "Distribution of Motivation by Drug",
       x = "Motivation Score", y = "Count") +
  theme_minimal() +
  scale_fill_brewer(palette = "Set2")
```

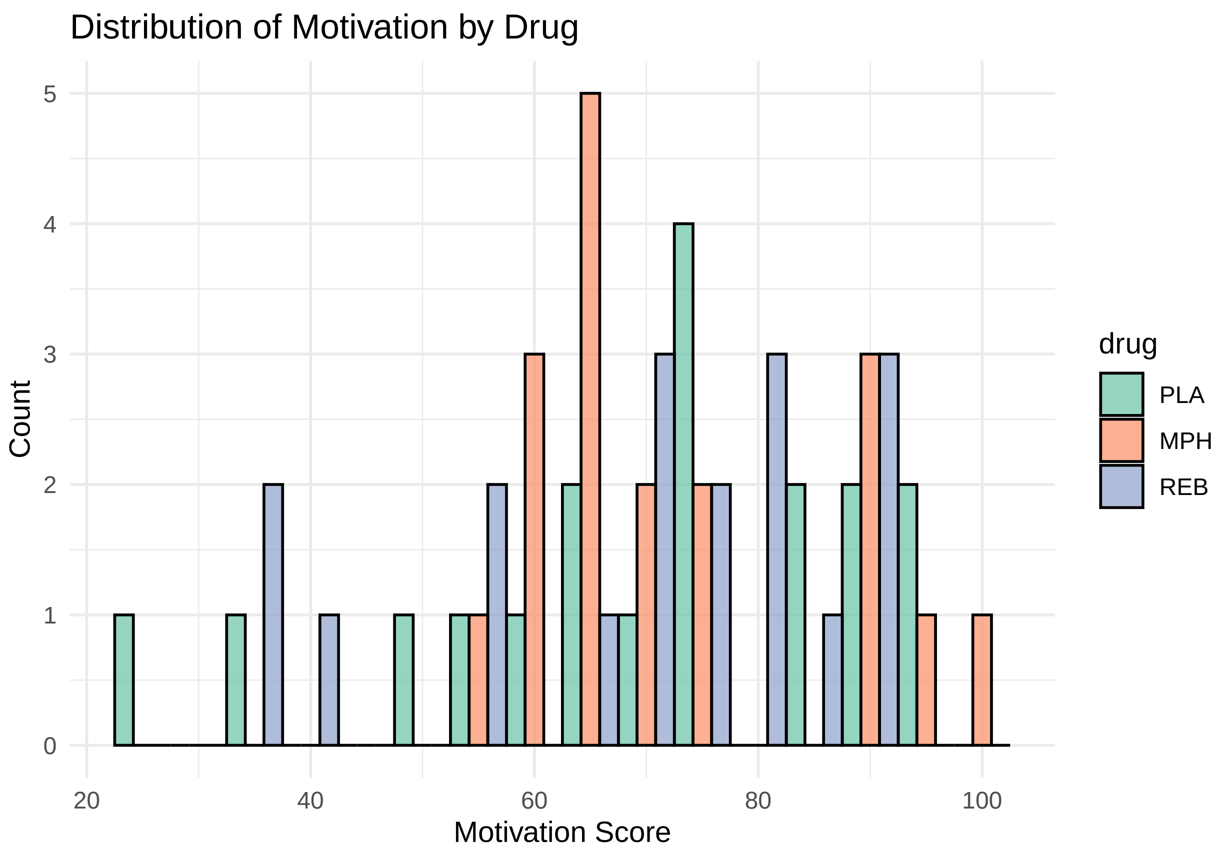

4.32 DATA ANALYSIS (MOTIVATION)

```
#-------------------------------------------------MODELS

# Model with lmer model
model_motivation <- lmer(scale(motivation) ~ drug + (1 | record_id), data = data_perf)
summary(model_motivation) # this model takes the record ID in consideration
```

```
## Linear mixed model fit by REML. t-tests use Satterthwaite's method [
## lmerModLmerTest]
## Formula: scale(motivation) ~ drug + (1 | record_id)
##    Data: data_perf
##
## REML criterion at convergence: 128
##
## Scaled residuals:
##     Min      1Q  Median      3Q     Max
## -1.8649 -0.5575  0.1042  0.4798  2.0737
##
## Random effects:
##  Groups    Name        Variance Std.Dev.
##  record_id (Intercept) 0.7228   0.8502
##  Residual              0.3015   0.5491
## Number of obs: 54, groups:  record_id, 18
```

```
##
## Fixed effects:
##             Estimate Std. Error       df t value Pr(>|t|)
## (Intercept) -0.04954    0.23855 25.55285  -0.208    0.837
## drugMPH      0.21324    0.18303 34.00000   1.165    0.252
## drugREB     -0.06462    0.18303 34.00000  -0.353    0.726
##
## Correlation of Fixed Effects:
##         (Intr) drgMPH
## drugMPH -0.384
## drugREB -0.384  0.500
```

```
# transform the data with log
data_perf$motivation_logit <- log((data_perf$motivation + 0.01) / (100 - data_perf$motivation + 0.01))
model_motivation_trans <- lmer(log(motivation) ~ drug + (1 | record_id), data = data_perf) # THIS ONE L
summary(model_motivation_trans)
```

```
## Linear mixed model fit by REML. t-tests use Satterthwaite's method [
## lmerModLmerTest]
## Formula: log(motivation) ~ drug + (1 | record_id)
##    Data: data_perf
##
## REML criterion at convergence: 7.9
##
## Scaled residuals:
##     Min      1Q  Median      3Q     Max
## -2.6470 -0.4757  0.1683  0.4562  2.0774
##
## Random effects:
##  Groups    Name        Variance Std.Dev.
##  record_id (Intercept) 0.05422  0.2328
##  Residual              0.03140  0.1772
## Number of obs: 54, groups:  record_id, 18
##
## Fixed effects:
##              Estimate Std. Error        df t value Pr(>|t|)
## (Intercept)  4.195490   0.068968 28.301336  60.833   <2e-16 ***
## drugMPH      0.087424   0.059067 34.000000   1.480    0.148
## drugREB     -0.005505   0.059067 34.000000  -0.093    0.926
## ---
## Signif. codes:  0 '***' 0.001 '**' 0.01 '*' 0.05 '.' 0.1 ' ' 1
##
## Correlation of Fixed Effects:
##         (Intr) drgMPH
## drugMPH -0.428
## drugREB -0.428  0.500
```

```
summarize_lmer_effects(model_motivation_trans, inverse = exp)
```

```
##          Term Estimate   SE Estimate_inv CI_lower CI_upper p_value
## 1 (Intercept)     4.20 0.07        66.39    57.99    75.99   0.000
## 2     drugMPH     0.09 0.06         1.09     0.97     1.23   0.148
## 3     drugREB    -0.01 0.06         0.99     0.89     1.12   0.926
```

```r
# quickly check the plot to understand what happends
preds_motivation <- ggpredict(model_motivation_trans, terms = c("drug"))
```

```
## Model has log-transformed response. Back-transforming predictions to
##   original response scale. Standard errors are still on the transformed
##   scale.
```

```r
plot(preds_motivation)
```

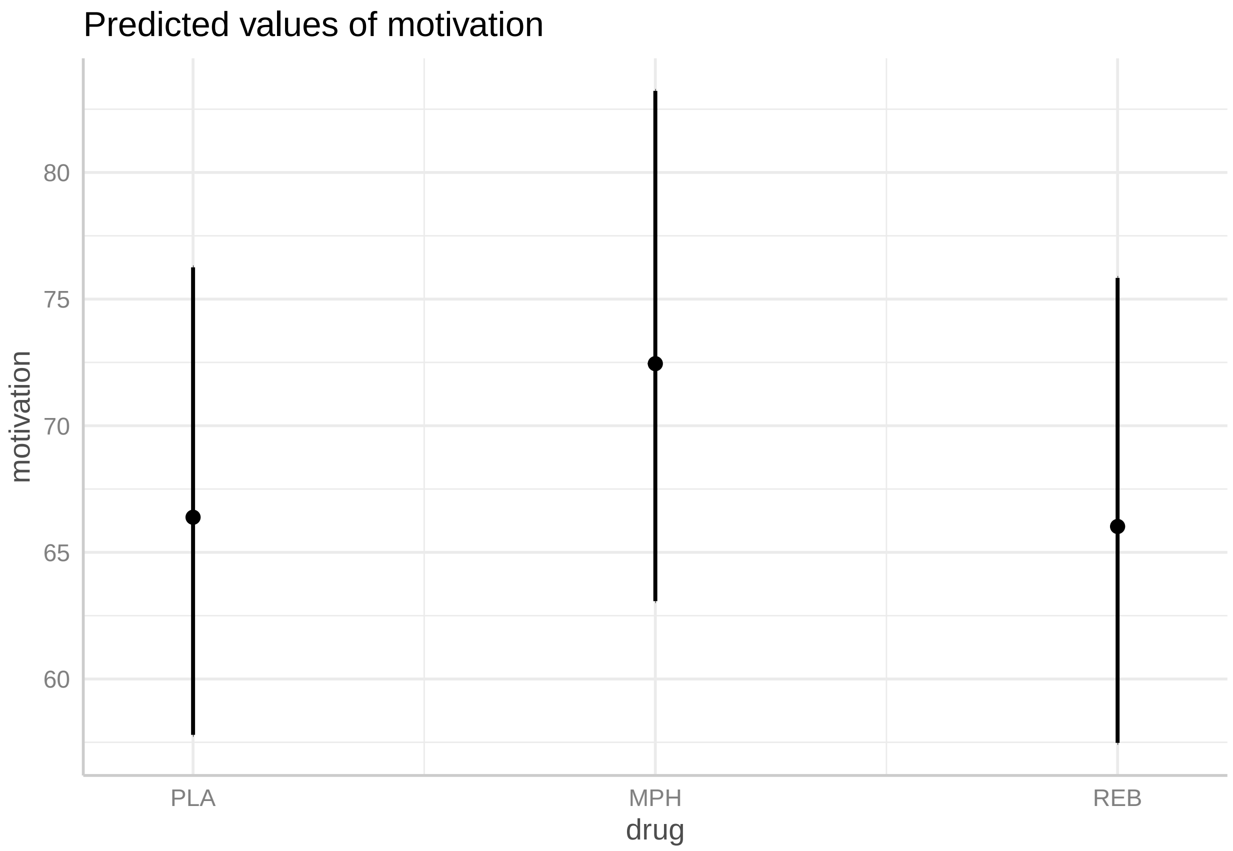


```r
#-----------------------------------------------------ASSUMPTION CHECK MODELS MOTIVATION------------
# Simulate residuals
sim_res2 <- simulateResiduals(model_motivation)
# Plot diagnostics
plot(sim_res2) # assumptions not met. we try with log
```

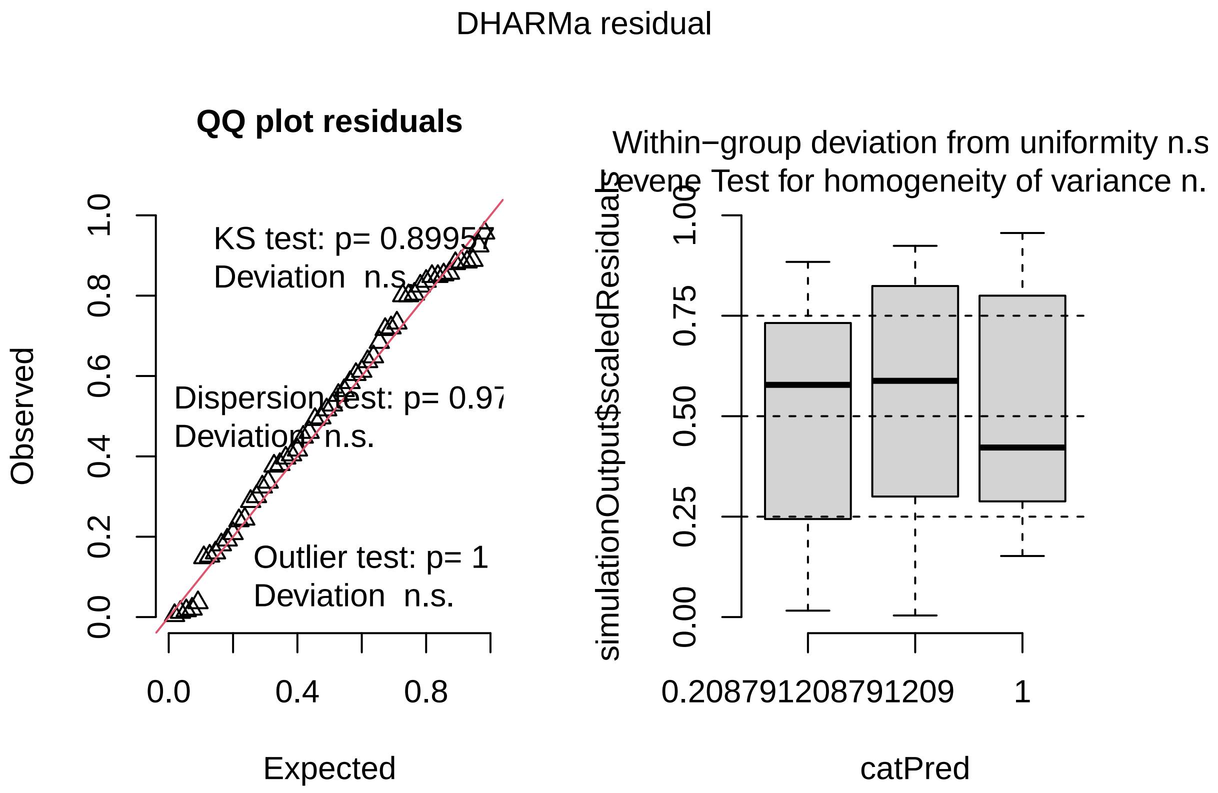


```r
# Simulate residuals
sim_res2 <- simulateResiduals(model_motivation_trans) # check!
# Plot diagnostics
plot(sim_res2) # assumptions met
```

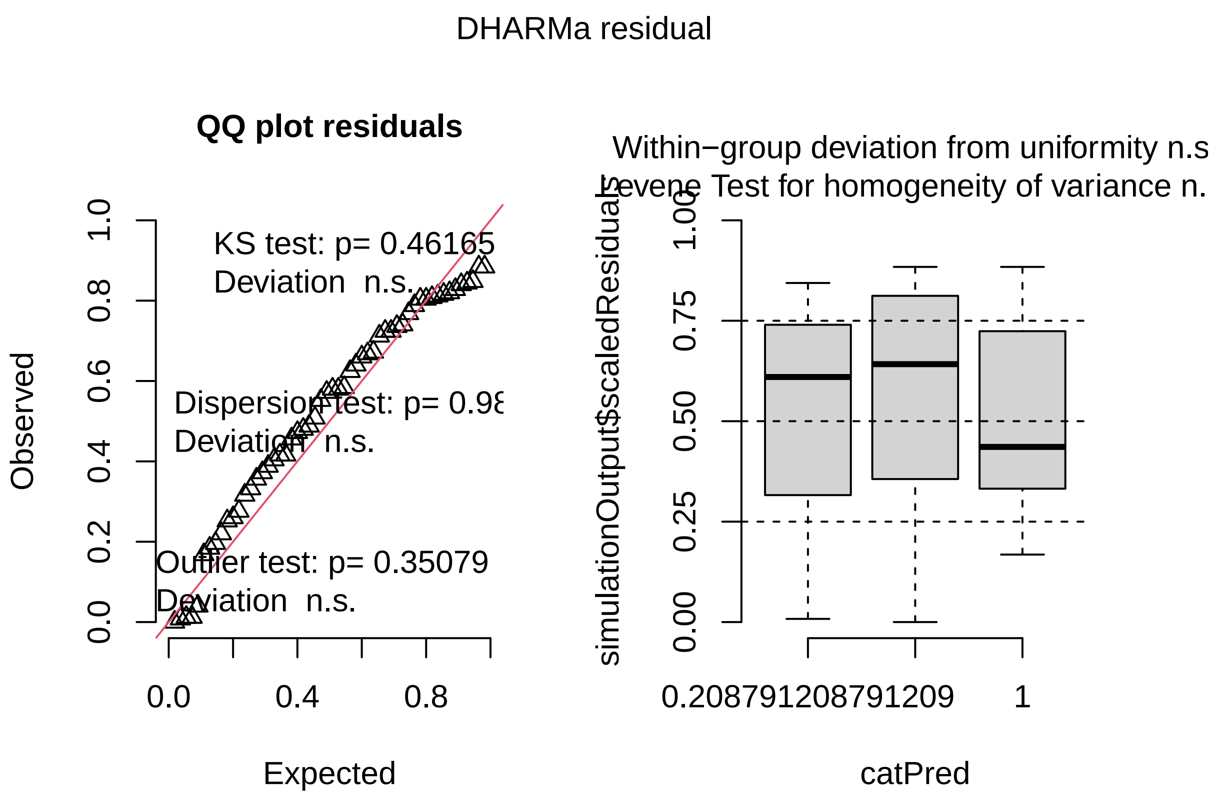

4.4 MOOD

4.41 DATA DESCRIPTIVES _ ALL SUBSCALES

```
#------------------------------------------------------------DESCRIPTIVES MOOD

# two datapoints pre-and-post fatigue with BRUMS having five subscales

# Compute descriptives of all subscales
mean_data_BRUMS <- data_subj %>%
  group_by(drug, time) %>%
  summarise(
    tension_mean = round(mean(BRUMS_tension, na.rm = TRUE), 2),
    tension_sd = round(sd(BRUMS_tension, na.rm = TRUE), 2),
    depression_mean = round(mean(BRUMS_depression, na.rm = TRUE), 2),
    depression_sd = round(sd(BRUMS_depression, na.rm = TRUE), 2),
    anger_mean = round(mean(BRUMS_anger, na.rm = TRUE), 2),
    anger_sd = round(sd(BRUMS_anger, na.rm = TRUE), 2),
    fatigue_mean = round(mean(BRUMS_fatigue, na.rm = TRUE), 2),
    fatigue_sd = round(sd(BRUMS_fatigue, na.rm = TRUE), 2),
    confusion_mean = round(mean(BRUMS_confusion, na.rm = TRUE), 2),
    confusion_sd = round(sd(BRUMS_confusion, na.rm = TRUE), 2),
    vigour_mean = round(mean(BRUMS_vigour, na.rm = TRUE), 2),
```

```r
    vigour_sd = round(sd(BRUMS_vigour, na.rm = TRUE), 2),
  )
```

```
## `summarise()` has grouped output by 'drug'. You can override using the
## `.groups` argument.
```

```r
view(mean_data_BRUMS)

# the data is not representative with mean and sd so interquartile ranges may be better here. Many subs

median_data_BRUMS <- data_subj %>%
  group_by(drug, time) %>%
  summarise(
    tension_median = round(median(BRUMS_tension, na.rm = TRUE), 2),
    tension_Q1 = round(quantile(BRUMS_tension, 0.25, na.rm = TRUE), 2),
    tension_Q3 = round(quantile(BRUMS_tension, 0.75, na.rm = TRUE), 2),

    depression_median = round(median(BRUMS_depression, na.rm = TRUE), 2),
    depression_Q1 = round(quantile(BRUMS_depression, 0.25, na.rm = TRUE), 2),
    depression_Q3 = round(quantile(BRUMS_depression, 0.75, na.rm = TRUE), 2),

    anger_median = round(median(BRUMS_anger, na.rm = TRUE), 2),
    anger_Q1 = round(quantile(BRUMS_anger, 0.25, na.rm = TRUE), 2),
    anger_Q3 = round(quantile(BRUMS_anger, 0.75, na.rm = TRUE), 2),

    fatigue_median = round(median(BRUMS_fatigue, na.rm = TRUE), 2),
    fatigue_Q1 = round(quantile(BRUMS_fatigue, 0.25, na.rm = TRUE), 2),
    fatigue_Q3 = round(quantile(BRUMS_fatigue, 0.75, na.rm = TRUE), 2),

    confusion_median = round(median(BRUMS_confusion, na.rm = TRUE), 2),
    confusion_Q1 = round(quantile(BRUMS_confusion, 0.25, na.rm = TRUE), 2),
    confusion_Q3 = round(quantile(BRUMS_confusion, 0.75, na.rm = TRUE), 2),

    vigour_median = round(median(BRUMS_vigour, na.rm = TRUE), 2),
    vigour_Q1 = round(quantile(BRUMS_vigour, 0.25, na.rm = TRUE), 2),
    vigour_Q3 = round(quantile(BRUMS_vigour, 0.75, na.rm = TRUE), 2)
  )
```

```
## `summarise()` has grouped output by 'drug'. You can override using the
## `.groups` argument.
```

```r
median_data_BRUMS <- median_data_BRUMS %>%
  mutate(
    tension_summary = paste0(tension_median, " [", tension_Q1, "-", tension_Q3, "]"),
    depression_summary = paste0(depression_median, " [", depression_Q1, "-", depression_Q3, "]"),
    anger_summary = paste0(anger_median, " [", anger_Q1, "-", anger_Q3, "]"),
    fatigue_summary = paste0(fatigue_median, " [", fatigue_Q1, "-", fatigue_Q3, "]"),
    confusion_summary = paste0(confusion_median, " [", confusion_Q1, "-", confusion_Q3, "]"),
    vigour_summary = paste0(vigour_median, " [", vigour_Q1, "-", vigour_Q3, "]")
  )

view(median_data_BRUMS)
```

4.42 DATA VISUALIZATION __ TENSION

```
#--------------------------------------------------------- TENSION Visualize by boxplot AND HISTOGRAM


# Histogram + Boxplot
brums_tension_plot <-
  ggplot(data_subj, aes(x = factor(time), y = BRUMS_tension, fill = drug)) +
  geom_boxplot(alpha = 0.3, outlier.size = 2) +
  geom_jitter(aes(color = drug),
              position = position_jitterdodge(jitter.width = 0.2, dodge.width = 0.8),
              size = 1.5, alpha = 0.6) +
  stat_summary(fun = mean, geom = "point",
               position = position_dodge(width = 0.8),
               size = 3, shape = 18, aes(color = drug)) +
  labs(title = "tension by Time and Drug",
       x = "Time", y = "tension") +
  theme_minimal() +
  scale_fill_brewer(palette = "Set2") +
  scale_color_brewer(palette = "Set2")

# Histogram below
brums_tension_hist <-
  ggplot(data_subj, aes(x = BRUMS_tension, fill = drug)) +
  geom_histogram(binwidth = 1, position = "dodge", color = "black", alpha = 0.7) +
  facet_wrap(~ time) +
  labs(title = "Depression Distribution by Time and Drug",
       x = "tension Score", y = "Count") +
  theme_minimal() +
  scale_fill_brewer(palette = "Set2")

# Combine them
brums_tension_plot / brums_tension_hist + plot_layout(heights = c(2, 1))
```

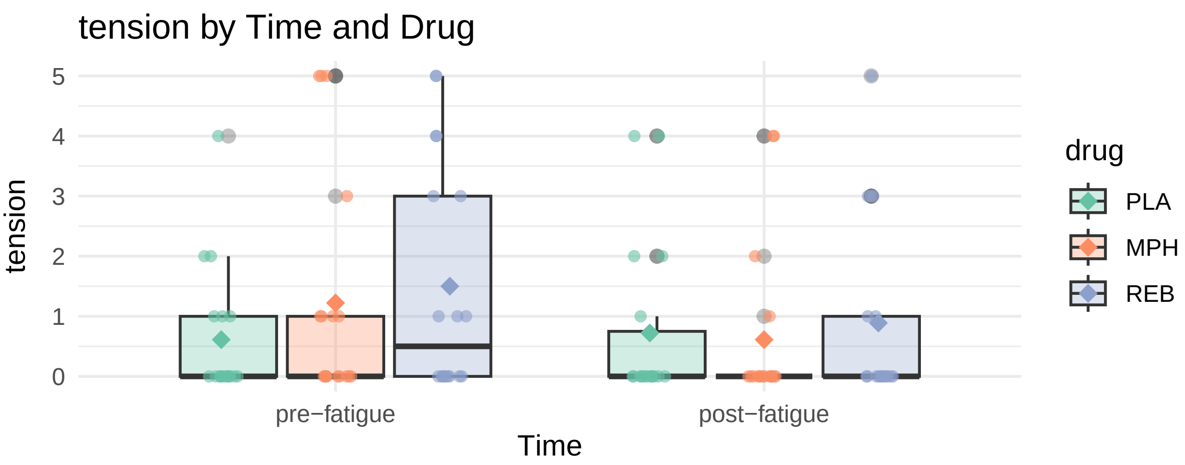

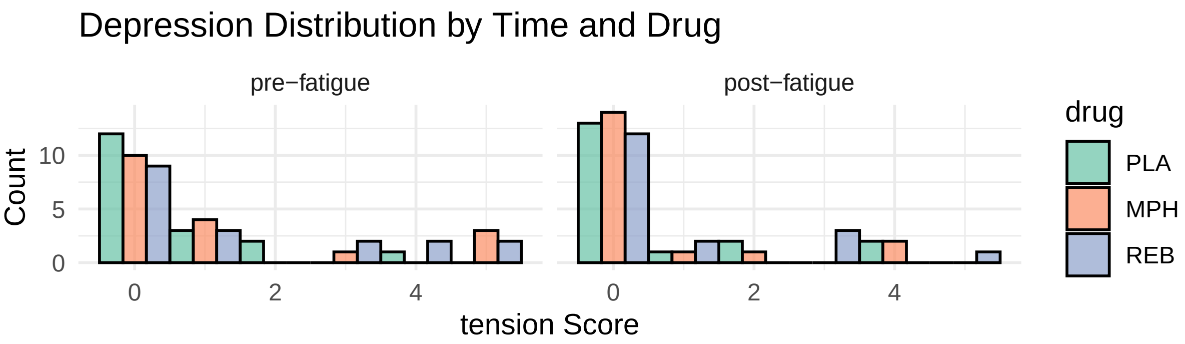

4.43 DATA ANALYSES _ TENSION

```
#--------------------------------------MODELS

# Best model for this type of data after visualization. Data is definitely not normal so we cannot use
model_tension <- glmmTMB(BRUMS_tension ~ drug * time + (1 | record_id),
                 family = nbinom1, data = data_subj,
                 control = glmmTMBControl(optimizer = optim, optArgs = list(method = "BFGS"))) # got an

summary(model_tension)
```

```
##  Family: nbinom1  ( log )
## Formula:          BRUMS_tension ~ drug * time + (1 | record_id)
## Data: data_subj
##
##      AIC       BIC    logLik -2*log(L)  df.resid
##    220.2     241.7    -102.1     204.2       100
##
## Random effects:
##
## Conditional model:
##  Groups    Name        Variance Std.Dev.
##  record_id (Intercept) 3.075    1.754
## Number of obs: 108, groups:  record_id, 18
```

```
##
## Dispersion parameter for nbinom1 family (): 0.000289
##
## Conditional model:
##                            Estimate Std. Error z value Pr(>|z|)
## (Intercept)                 -1.7400     0.5906  -2.946  0.00322 **
## drugMPH                      0.6925     0.3693   1.875  0.06075 .
## drugREB                      0.8975     0.3577   2.509  0.01210 *
## timepost-fatigue             0.1673     0.4096   0.408  0.68304
## drugMPH:timepost-fatigue    -0.8604     0.5516  -1.560  0.11879
## drugREB:timepost-fatigue    -0.6908     0.5171  -1.336  0.18159
## ---
## Signif. codes:  0 '***' 0.001 '**' 0.01 '*' 0.05 '.' 0.1 ' ' 1
```

```r
report_glmmTMB_effects(model_tension)
```

```
##                        Term Estimate_log SE_log  IRR CI_lower CI_upper p_value
## 1               (Intercept)       -1.740  0.591 0.18     0.06     0.56 0.00322
## 2                   drugMPH        0.693  0.369 2.00     0.97     4.12 0.06070
## 3                   drugREB        0.898  0.358 2.45     1.22     4.95 0.01210
## 4          timepost-fatigue        0.167  0.410 1.18     0.53     2.64 0.68300
## 5 drugMPH:timepost-fatigue       -0.860  0.552 0.42     0.14     1.25 0.11900
## 6 drugREB:timepost-fatigue       -0.691  0.517 0.50     0.18     1.38 0.18200
```

```r
#----------------------------------------ASSUMPTION check model 3
# Simulate residuals
sim_res2 <- simulateResiduals(model_tension) # check!
# Plot diagnostics
plot(sim_res2) # assumptions met
```

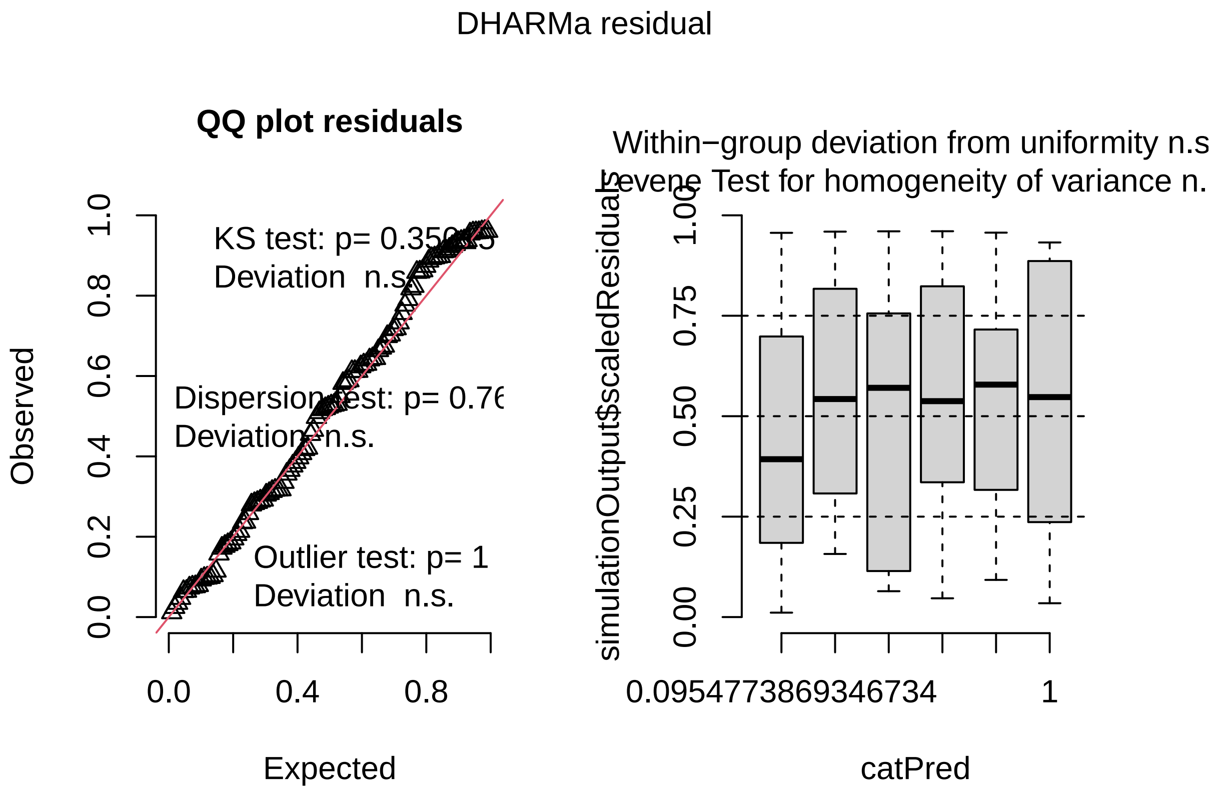


```r
# Test for overdispersion
testDispersion(sim_res2) # 1.00, p = 0.792  check!
```

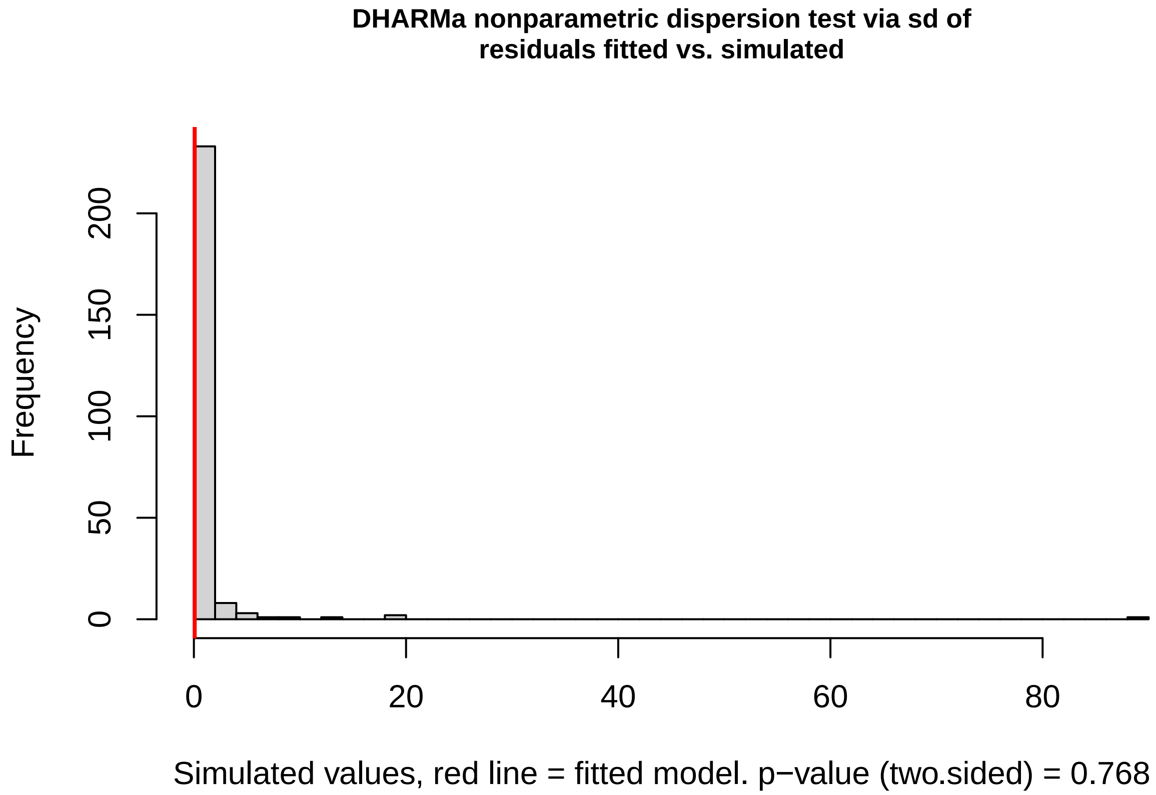


```
##
##  DHARMa nonparametric dispersion test via sd of residuals fitted vs.
##  simulated
##
## data:  simulationOutput
## dispersion = 0.078927, p-value = 0.768
## alternative hypothesis: two.sided
```

```
testZeroInflation(sim_res2)
```

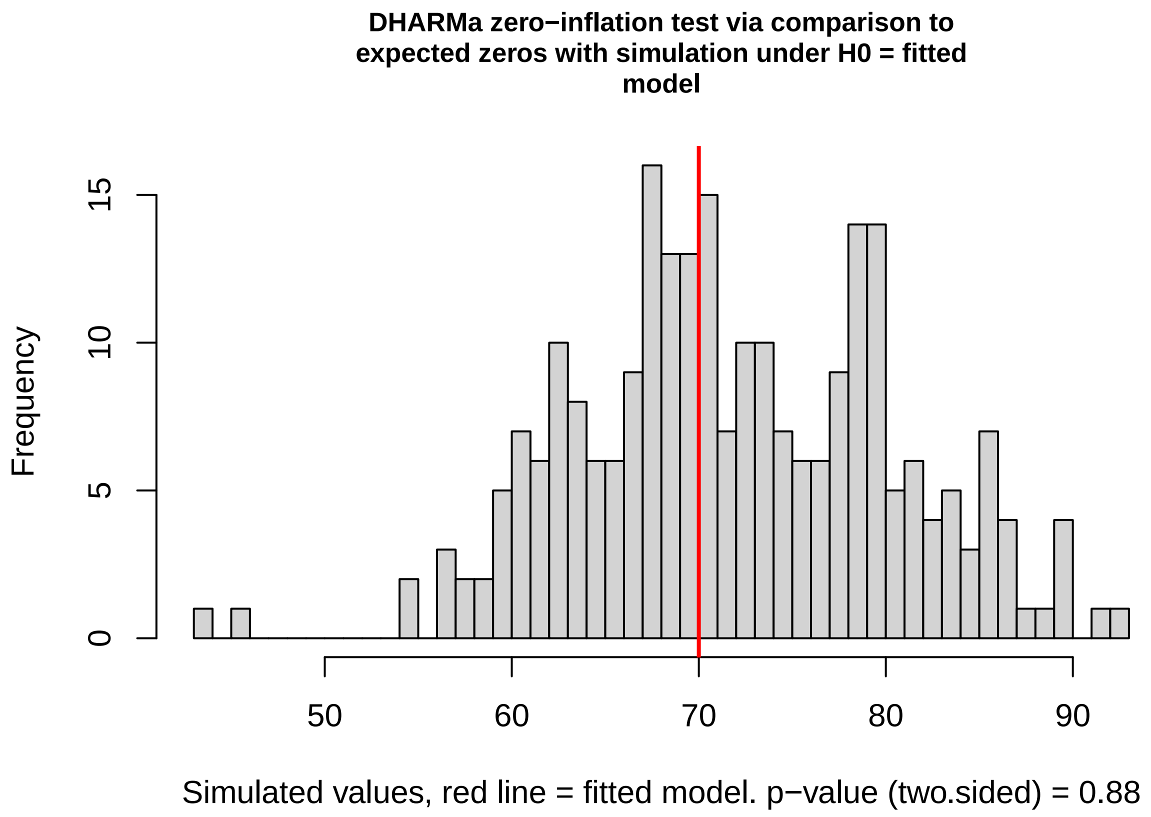


```
##
##  DHARMa zero-inflation test via comparison to expected zeros with
##  simulation under H0 = fitted model
##
## data:  simulationOutput
## ratioObsSim = 0.96605, p-value = 0.88
## alternative hypothesis: two.sided
```

4.44 DATA VISUALIZATION- DEPRESSION

```
##--------------------------- DEPRESSION

# Boxplot
brums_depression_plot <-
  ggplot(data_subj, aes(x = factor(time), y = BRUMS_depression, fill = drug)) +
  geom_boxplot(alpha = 0.3, outlier.size = 2) +
  geom_jitter(aes(color = drug),
              position = position_jitterdodge(jitter.width = 0.2, dodge.width = 0.8),
              size = 1.5, alpha = 0.6) +
  stat_summary(fun = mean, geom = "point",
               position = position_dodge(width = 0.8),
               size = 3, shape = 18, aes(color = drug)) +
  labs(title = "Depression by Time and Drug",
       x = "Time", y = "Depression") +
```

```r
  theme_minimal() +
  scale_fill_brewer(palette = "Set2") +
  scale_color_brewer(palette = "Set2")

# Histogram below
brums_depression_hist <-
  ggplot(data_subj, aes(x = BRUMS_depression, fill = drug)) +
  geom_histogram(binwidth = 1, position = "dodge", color = "black", alpha = 0.7) +
  facet_wrap(~ time) +
  labs(title = "Depression Distribution by Time and Drug",
       x = "Depression Score", y = "Count") +
  theme_minimal() +
  scale_fill_brewer(palette = "Set2")

# Combine them
brums_depression_plot / brums_depression_hist + plot_layout(heights = c(2, 1))
```

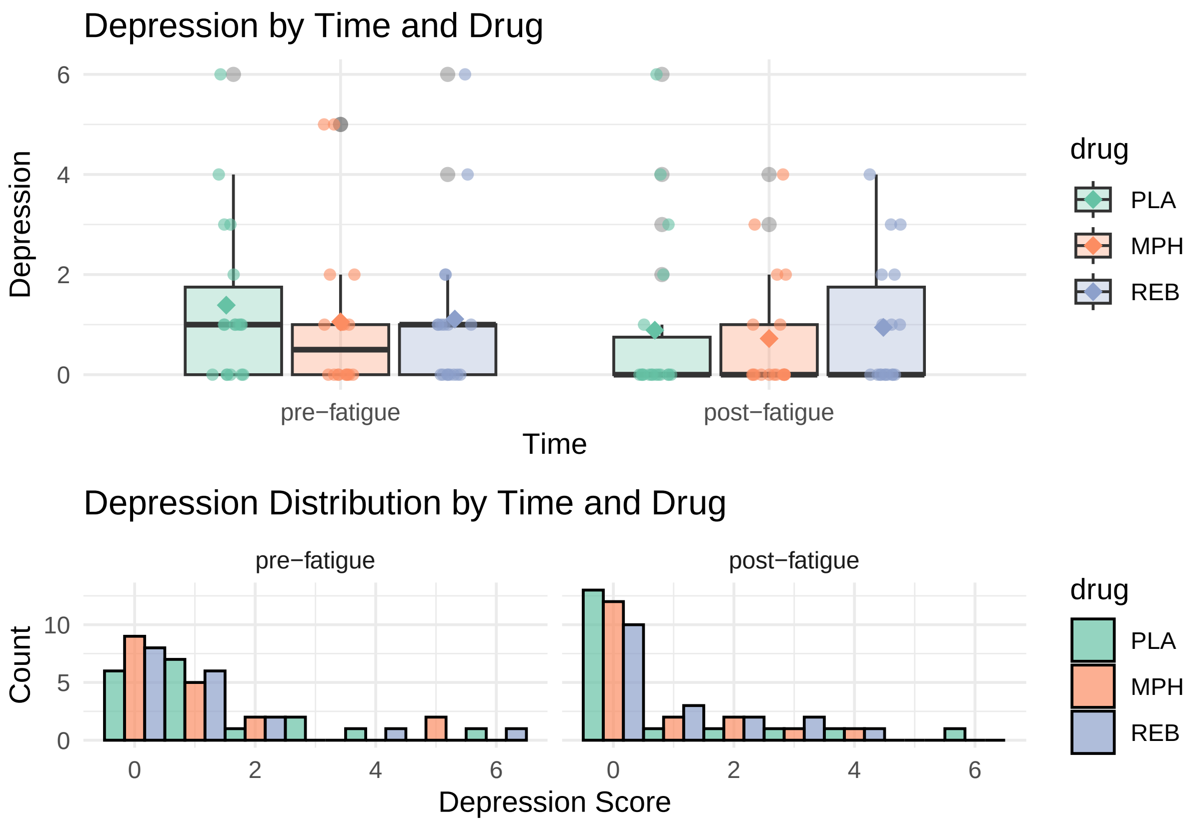

4.45 DATA ANALYSES __ DEPRESSION

```r
# -----------------------------------------------------Models

# Best model for this type of data, handles zero inflation too.
model_depression <- glmmTMB(
```

```r
  BRUMS_depression ~ drug * time + (1 | record_id),
  family = nbinom1,
  data = data_subj
)
summary(model_depression)
```

```
##  Family: nbinom1  ( log )
## Formula:          BRUMS_depression ~ drug * time + (1 | record_id)
## Data: data_subj
##
##      AIC       BIC    logLik -2*log(L)  df.resid
##    269.5     291.0    -126.8     253.5       100
##
## Random effects:
##
## Conditional model:
##  Groups    Name        Variance Std.Dev.
##  record_id (Intercept) 1.076    1.037
## Number of obs: 108, groups:  record_id, 18
##
## Dispersion parameter for nbinom1 family (): 0.129
##
## Conditional model:
##                          Estimate Std. Error z value Pr(>|z|)
## (Intercept)              -0.22679    0.35282  -0.643    0.520
## drugMPH                  -0.25697    0.32189  -0.798    0.425
## drugREB                  -0.19592    0.31658  -0.619    0.536
## timepost-fatigue         -0.44729    0.34193  -1.308    0.191
## drugMPH:timepost-fatigue  0.01554    0.51795   0.030    0.976
## drugREB:timepost-fatigue  0.27092    0.48592   0.557    0.577
```

```r
report_glmmTMB_effects(model_depression)
```

```
##                        Term Estimate_log SE_log  IRR CI_lower CI_upper p_value
## 1               (Intercept)       -0.227  0.353 0.80     0.40     1.59   0.520
## 2                   drugMPH       -0.257  0.322 0.77     0.41     1.45   0.425
## 3                   drugREB       -0.196  0.317 0.82     0.44     1.53   0.536
## 4          timepost-fatigue       -0.447  0.342 0.64     0.33     1.25   0.191
## 5 drugMPH:timepost-fatigue        0.016  0.518 1.02     0.37     2.80   0.976
## 6 drugREB:timepost-fatigue        0.271  0.486 1.31     0.51     3.40   0.577
```

```r
#---------------------------------------ASSUMPTION check model depression
# Simulate residuals
sim_res2 <- simulateResiduals(model_depression) # check!
# Plot diagnostics
plot(sim_res2) # assumptions met
```

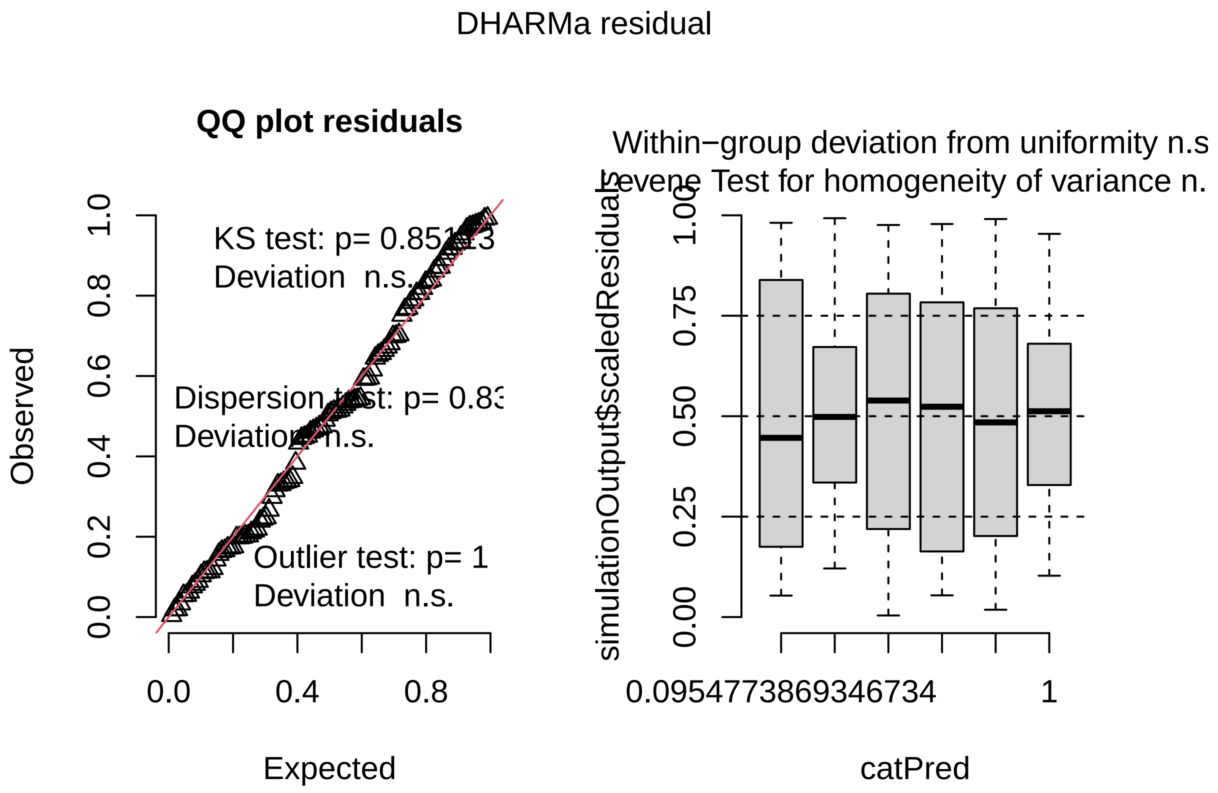


```
# Test for overdispersion
testDispersion(sim_res2) # 1.0152, p = 0.96 (whenever >1 there is overdispersion); check!
```

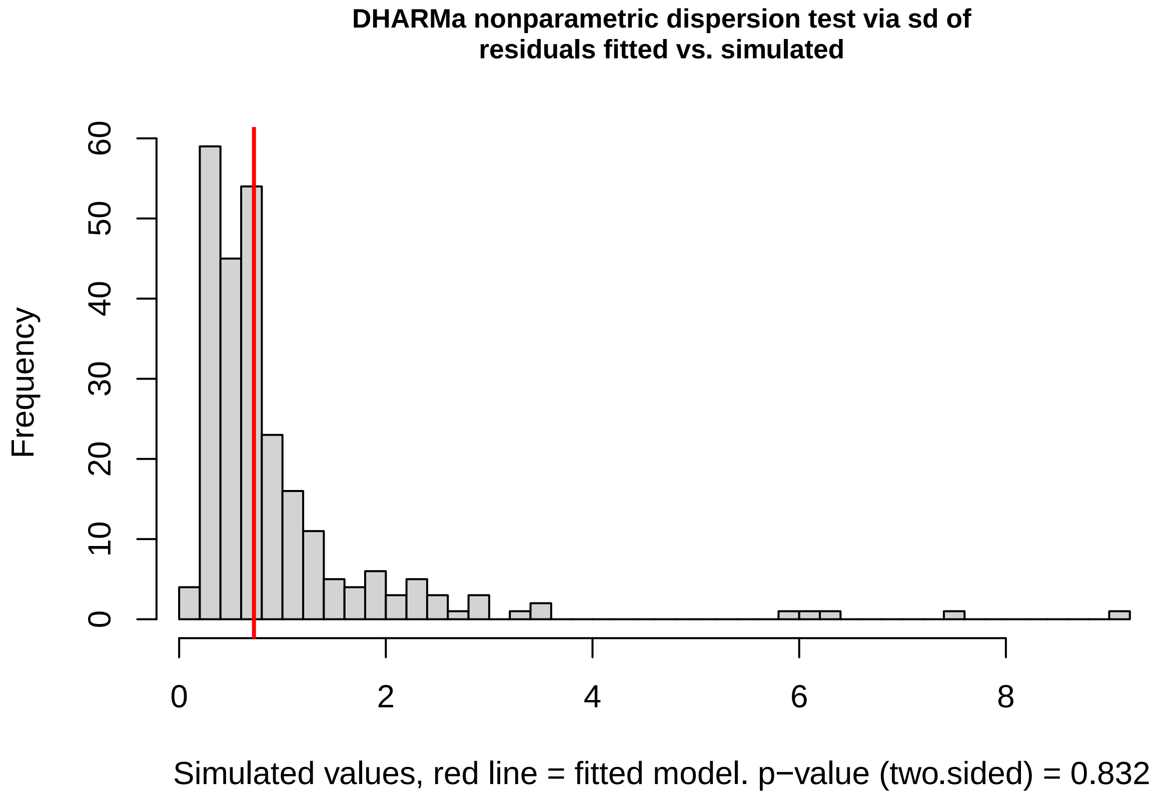


```
##
##  DHARMa nonparametric dispersion test via sd of residuals fitted vs.
##  simulated
##
## data:  simulationOutput
## dispersion = 0.75806, p-value = 0.832
## alternative hypothesis: two.sided
```

```
testZeroInflation(sim_res2)
```

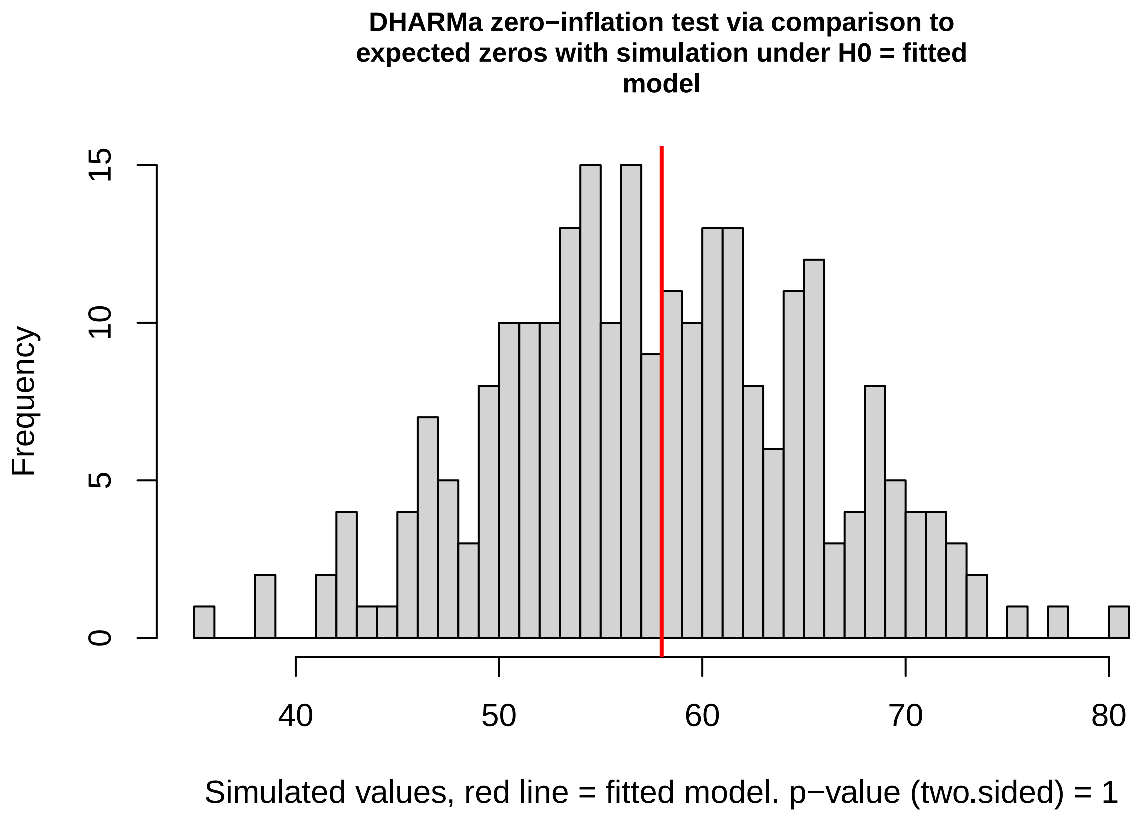


```
##
##  DHARMa zero-inflation test via comparison to expected zeros with
##  simulation under H0 = fitted model
##
## data:  simulationOutput
## ratioObsSim = 0.99492, p-value = 1
## alternative hypothesis: two.sided
```

4.46 DATA VISUALIZATION- ANGER

```
## ---------------------------ANGER
brums_anger_plot <-
  ggplot(data_subj, aes(x = factor(time), y = BRUMS_anger, fill = drug)) +
  geom_boxplot(alpha = 0.3, outlier.size = 2) +
  geom_jitter(aes(color = drug),
              position = position_jitterdodge(jitter.width = 0.2, dodge.width = 0.8),
              size = 1.5, alpha = 0.6) +
  stat_summary(fun = mean, geom = "point",
               position = position_dodge(width = 0.8),
               size = 3, shape = 18, aes(color = drug)) +
  labs(title = "Anger by Time and Drug",
       x = "Time", y = "Anger") +
  theme_minimal() +
  scale_fill_brewer(palette = "Set2") +
```

```r
  scale_color_brewer(palette = "Set2")

# Histogram
brums_anger_hist <-
  ggplot(data_subj, aes(x = BRUMS_anger, fill = drug)) +
  geom_histogram(binwidth = 1, position = "dodge", color = "black", alpha = 0.7) +
  facet_wrap(~ time) +
  labs(title = "Anger Distribution by Time and Drug",
       x = "Anger Score", y = "Count") +
  theme_minimal() +
  scale_fill_brewer(palette = "Set2")

# Combine plots
brums_anger_plot / brums_anger_hist + plot_layout(heights = c(2, 1))
```

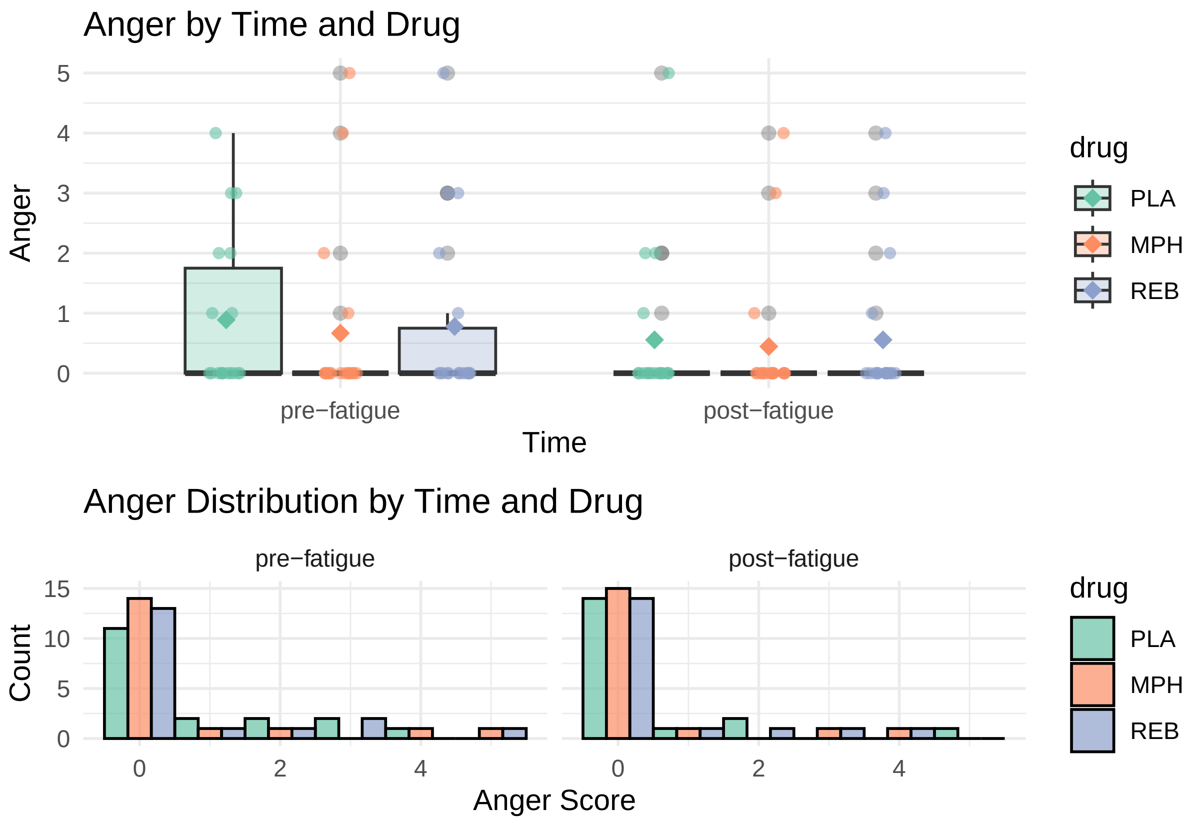

4.47 DATA ANALYSIS _ ANGER

```r
# ---------------------------------------------MODEL ANGER

# Best model for this type of data, handles zero inflation too.
model_anger <- glmmTMB(
  BRUMS_anger ~ drug * time + (1 | record_id),
  family = nbinom1,
```

```r
  data = data_subj
)
summary(model_anger)
```

```
##  Family: nbinom1  ( log )
## Formula:          BRUMS_anger ~ drug * time + (1 | record_id)
## Data: data_subj
##
##       AIC       BIC    logLik -2*log(L)  df.resid
##     178.8     200.3     -81.4     162.8       100
##
## Random effects:
##
## Conditional model:
##  Groups    Name        Variance Std.Dev.
##  record_id (Intercept) 5.983    2.446
## Number of obs: 108, groups:  record_id, 18
##
## Dispersion parameter for nbinom1 family (): 0.0707
##
## Conditional model:
##                          Estimate Std. Error z value Pr(>|z|)
## (Intercept)               -2.2080     0.9748  -2.265   0.0235 *
## drugMPH                   -0.3284     0.4209  -0.780   0.4353
## drugREB                   -0.1391     0.3755  -0.371   0.7109
## timepost-fatigue          -0.4815     0.4165  -1.156   0.2476
## drugMPH:timepost-fatigue   0.1026     0.6447   0.159   0.8735
## drugREB:timepost-fatigue   0.1396     0.5953   0.234   0.8146
## ---
## Signif. codes:  0 '***' 0.001 '**' 0.01 '*' 0.05 '.' 0.1 ' ' 1
```

```r
report_glmmTMB_effects(model_anger)
```

```
##                       Term Estimate_log SE_log  IRR CI_lower CI_upper p_value
## 1              (Intercept)       -2.208  0.975 0.11     0.02     0.74  0.0235
## 2                  drugMPH       -0.328  0.421 0.72     0.32     1.64  0.4350
## 3                  drugREB       -0.139  0.375 0.87     0.42     1.82  0.7110
## 4         timepost-fatigue       -0.482  0.417 0.62     0.27     1.40  0.2480
## 5 drugMPH:timepost-fatigue        0.103  0.645 1.11     0.31     3.92  0.8730
## 6 drugREB:timepost-fatigue        0.140  0.595 1.15     0.36     3.69  0.8150
```

```r
#------------------------------------------CHECK ASSUMPTIONS
# Simulate residuals
sim_res2 <- simulateResiduals(model_anger) # check!
# Plot diagnostics
plot(sim_res2) # assumptions met
```

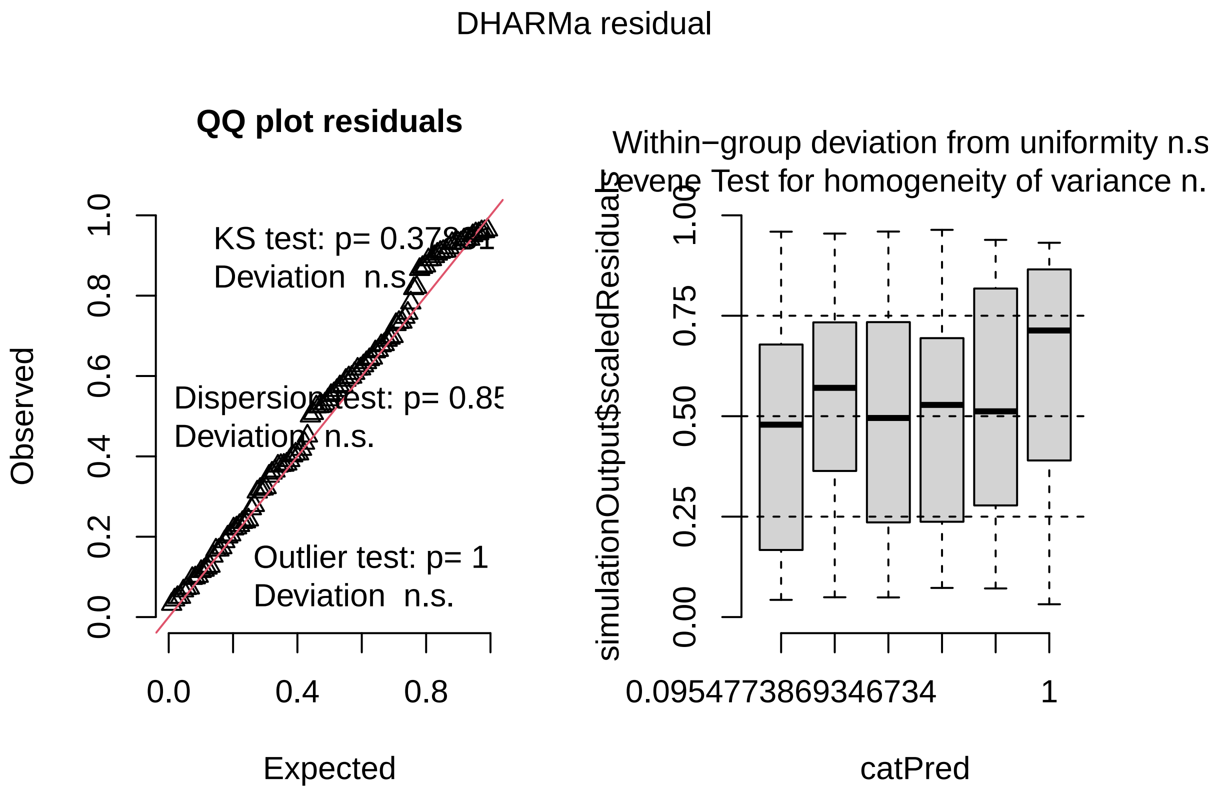


```
# Test for overdispersion
testDispersion(sim_res2) # 0.02, p = 0.848 (whenever >1 there is overdispersion); check! underdispersio
```

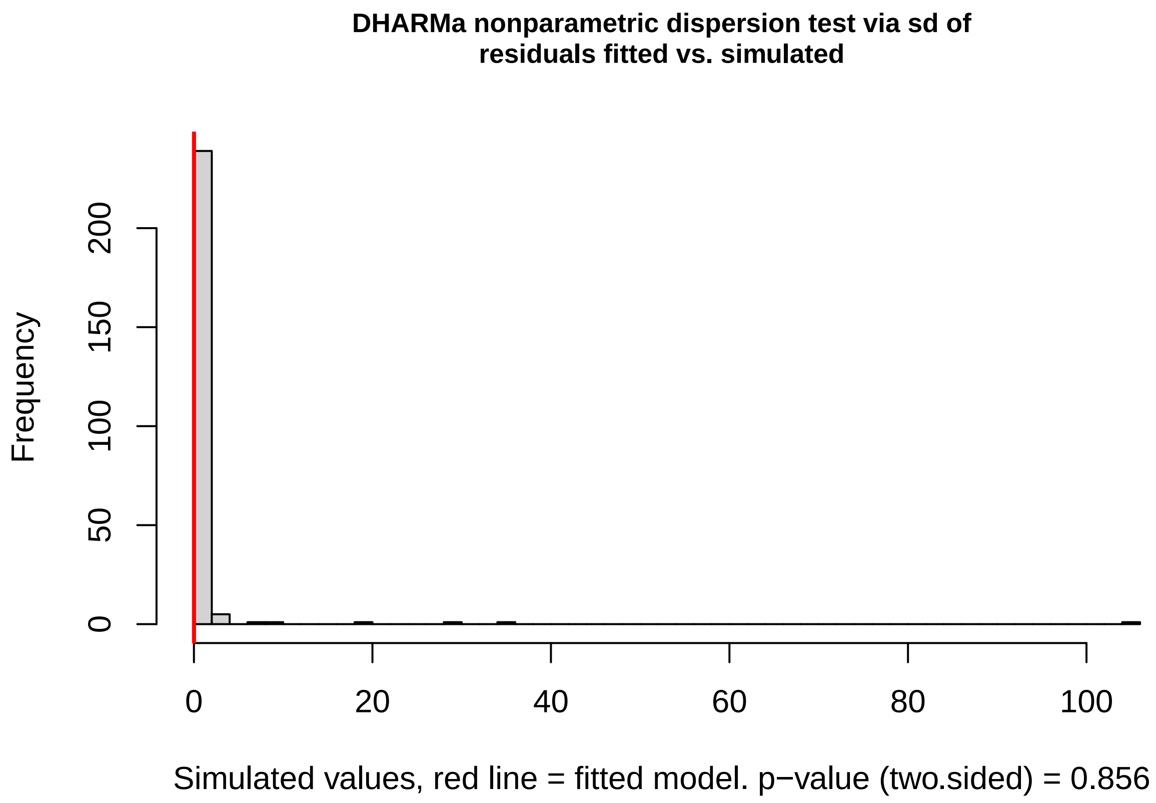


```
##
##  DHARMa nonparametric dispersion test via sd of residuals fitted vs.
##  simulated
##
## data:  simulationOutput
## dispersion = 0.0097417, p-value = 0.856
## alternative hypothesis: two.sided
```

```
testZeroInflation(sim_res2)
```

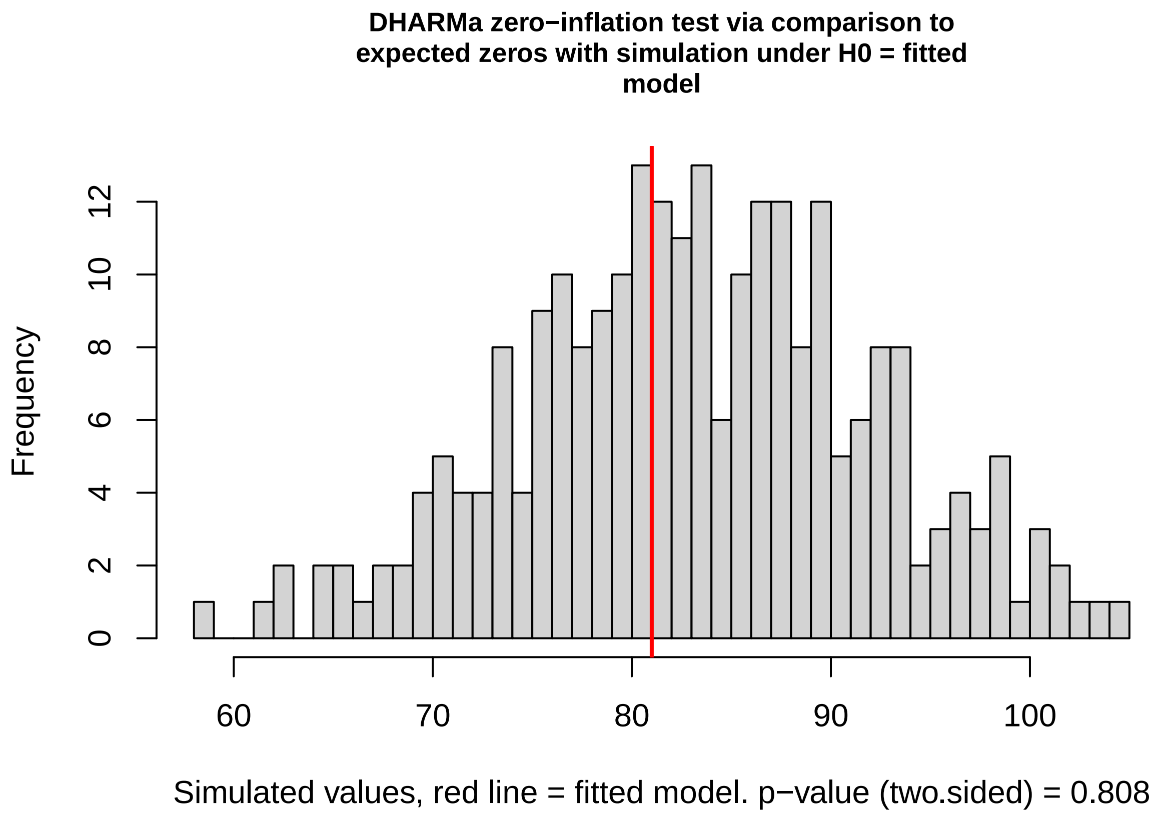


```
##
##  DHARMa zero-inflation test via comparison to expected zeros with
##  simulation under H0 = fitted model
##
## data:  simulationOutput
## ratioObsSim = 0.9677, p-value = 0.808
## alternative hypothesis: two.sided
```

4.48 DATA VISUALIZATION __ Fatigue

```
##---------------------------- FATIGUE
brums_fatigue_plot <-
  ggplot(data_subj, aes(x = factor(time), y = BRUMS_fatigue, fill = drug)) +
  geom_boxplot(alpha = 0.3, outlier.size = 2) +
  geom_jitter(aes(color = drug),
              position = position_jitterdodge(jitter.width = 0.2, dodge.width = 0.8),
              size = 1.5, alpha = 0.6) +
  stat_summary(fun = mean, geom = "point",
               position = position_dodge(width = 0.8),
               size = 3, shape = 18, aes(color = drug)) +
  labs(title = "Fatigue by Time and Drug",
       x = "Time", y = "Fatigue") +
  theme_minimal() +
  scale_fill_brewer(palette = "Set2") +
```

```
  scale_color_brewer(palette = "Set2")

# Histogram
brums_fatigue_hist <-
  ggplot(data_subj, aes(x = BRUMS_fatigue, fill = drug)) +
  geom_histogram(binwidth = 1, position = "dodge", color = "black", alpha = 0.7) +
  facet_wrap(~ time) +
  labs(title = "Fatigue Distribution by Time and Drug",
       x = "Fatigue Score", y = "Count") +
  theme_minimal() +
  scale_fill_brewer(palette = "Set2")

# Combine plots
brums_fatigue_plot / brums_fatigue_hist + plot_layout(heights = c(2, 1))
```

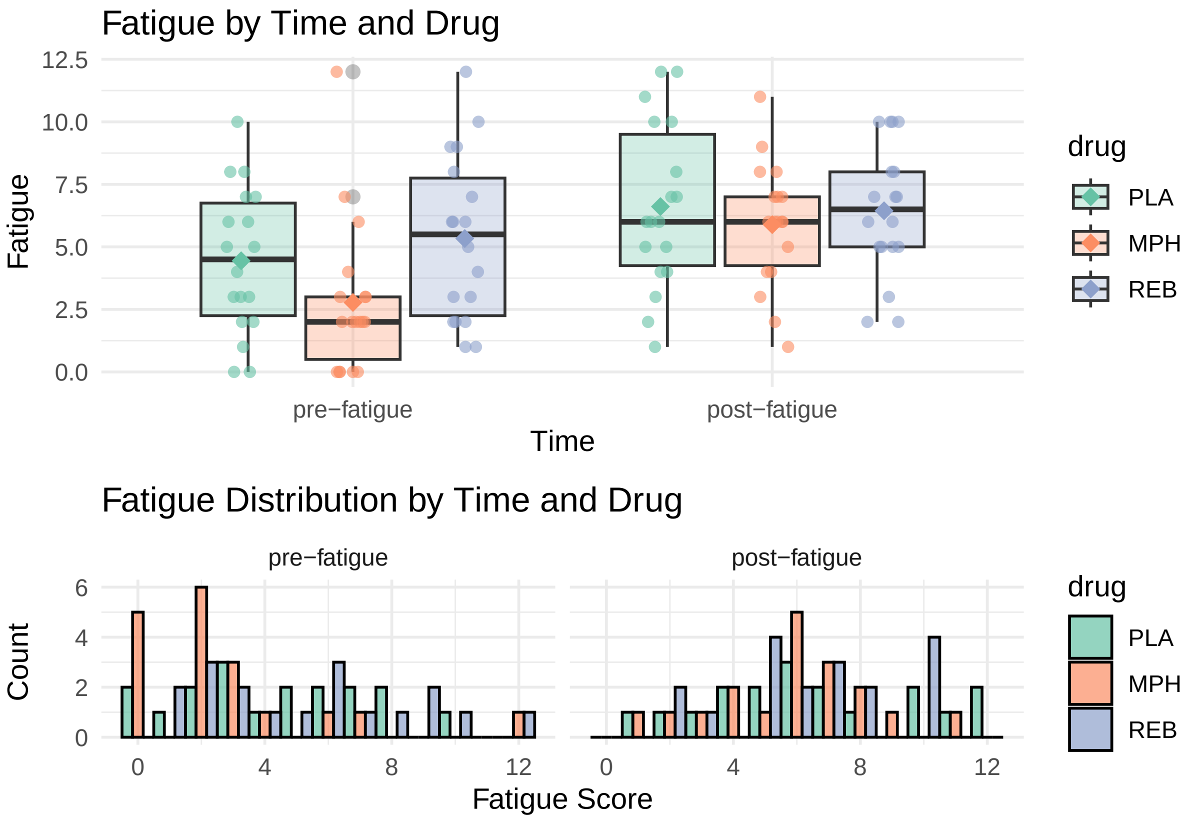

4.49 DATA ANALYSIS - fatigue

```
# Data looks parametric so test with lmer model
model_fatigue <- lmer(BRUMS_fatigue ~ drug * time + (1 | record_id), data = data_subj) # the data behav
summary(model_fatigue)
```

```
## Linear mixed model fit by REML. t-tests use Satterthwaite's method [
## lmerModLmerTest]
```

```
## Formula: BRUMS_fatigue ~ drug * time + (1 | record_id)
##    Data: data_subj
##
## REML criterion at convergence: 503.4
##
## Scaled residuals:
##      Min       1Q   Median       3Q      Max
## -2.14562 -0.55312 -0.07612  0.57927  2.36186
##
## Random effects:
##  Groups    Name        Variance Std.Dev.
##  record_id (Intercept) 3.562    1.887
##  Residual              5.243    2.290
## Number of obs: 108, groups:  record_id, 18
##
## Fixed effects:
##                            Estimate Std. Error      df t value Pr(>|t|)
## (Intercept)                  4.4444     0.6994 56.0990   6.355 3.97e-08 ***
## drugMPH                     -1.6667     0.7632 85.0000  -2.184  0.03174 *
## drugREB                      0.8889     0.7632 85.0000   1.165  0.24743
## timepost-fatigue             2.1667     0.7632 85.0000   2.839  0.00566 **
## drugMPH:timepost-fatigue     0.9444     1.0794 85.0000   0.875  0.38405
## drugREB:timepost-fatigue    -1.0556     1.0794 85.0000  -0.978  0.33089
## ---
## Signif. codes:  0 '***' 0.001 '**' 0.01 '*' 0.05 '.' 0.1 ' ' 1
##
## Correlation of Fixed Effects:
##             (Intr) drgMPH drgREB tmpst- dMPH:-
## drugMPH     -0.546
## drugREB     -0.546  0.500
## timepst-ftg -0.546  0.500  0.500
## drgMPH:tmp-  0.386 -0.707 -0.354 -0.707
## drgREB:tmp-  0.386 -0.354 -0.707 -0.707  0.500
```

```r
summarize_lmer_effects(model_fatigue)
```

```
##                        Term Estimate   SE Estimate_inv CI_lower CI_upper p_value
## 1               (Intercept)     4.44 0.70         4.44     3.07     5.82   0.000
## 2                   drugMPH    -1.67 0.76        -1.67    -3.16    -0.17   0.032
## 3                   drugREB     0.89 0.76         0.89    -0.61     2.38   0.247
## 4          timepost-fatigue     2.17 0.76         2.17     0.67     3.66   0.006
## 5 drugMPH:timepost-fatigue     0.94 1.08         0.94    -1.17     3.06   0.384
## 6 drugREB:timepost-fatigue    -1.06 1.08        -1.06    -3.17     1.06   0.331
```

```r
# check assumptions

# Simulate residuals
sim_res2 <- simulateResiduals(model_fatigue) # check!
# Plot diagnostics
plot(sim_res2) # assumptions met
```

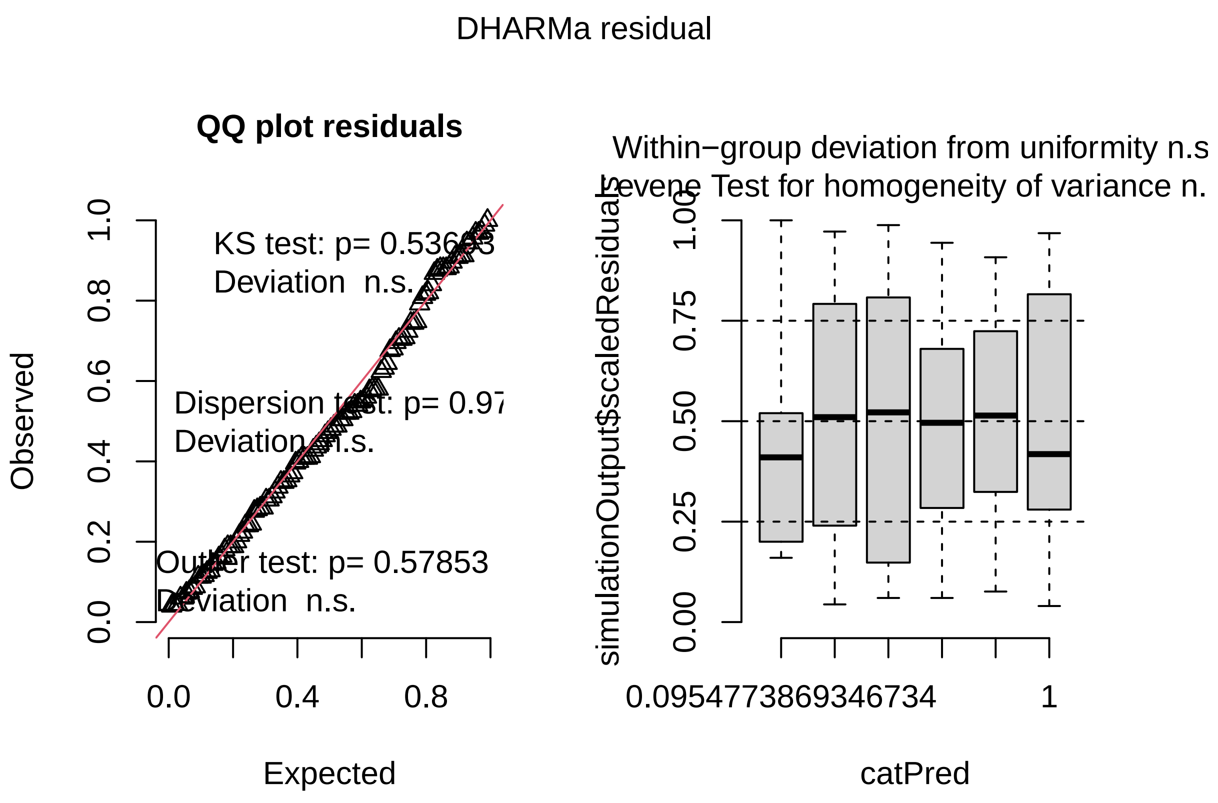

4.49b DATA VISUALIZATION CONFUSION

```
##---------------------------- CONFUSION
brums_confusion_plot <-
  ggplot(data_subj, aes(x = factor(time), y = BRUMS_confusion, fill = drug)) +
  geom_boxplot(alpha = 0.3, outlier.size = 2) +
  geom_jitter(aes(color = drug),
              position = position_jitterdodge(jitter.width = 0.2, dodge.width = 0.8),
              size = 1.5, alpha = 0.6) +
  stat_summary(fun = mean, geom = "point",
               position = position_dodge(width = 0.8),
               size = 3, shape = 18, aes(color = drug)) +
  labs(title = "Confusion by Time and Drug",
       x = "Time", y = "Confusion") +
  theme_minimal() +
  scale_fill_brewer(palette = "Set2") +
  scale_color_brewer(palette = "Set2")

# Histogram
brums_confusion_hist <-
  ggplot(data_subj, aes(x = BRUMS_confusion, fill = drug)) +
  geom_histogram(binwidth = 1, position = "dodge", color = "black", alpha = 0.7) +
  facet_wrap(~ time) +
  labs(title = "Confusion Distribution by Time and Drug",
```

```
      x = "Confusion Score", y = "Count") +
  theme_minimal() +
  scale_fill_brewer(palette = "Set2")

# Combine plots
brums_confusion_plot / brums_confusion_hist + plot_layout(heights = c(2, 1))
```

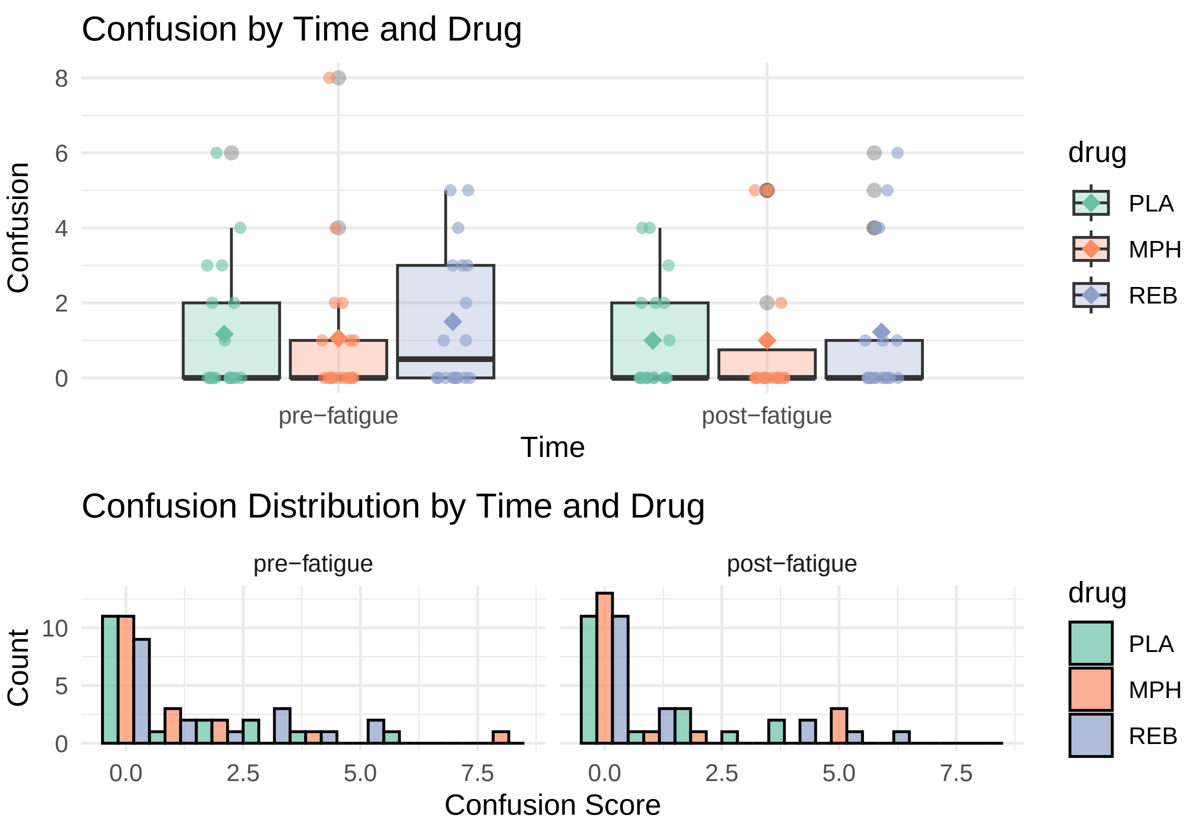

4.49c DATA ANALYSES CONFUSION

```
#-------------------------------------------------------------MODELS
# Best model for this type of data, handles zero inflation too.
model_confusion <- glmmTMB(
  BRUMS_confusion ~ drug * time + (1 | record_id),
  family = nbinom1,
  data = data_subj
)
summary(model_confusion)
```

```
##  Family: nbinom1  ( log )
## Formula:          BRUMS_confusion ~ drug * time + (1 | record_id)
## Data: data_subj
##
##      AIC      BIC   logLik -2*log(L)  df.resid
```

```
##     245.6     267.1    -114.8     229.6       100
##
## Random effects:
##
## Conditional model:
##  Groups    Name        Variance Std.Dev.
##  record_id (Intercept) 4.746    2.178
## Number of obs: 108, groups:  record_id, 18
##
## Dispersion parameter for nbinom1 family (): 0.0116
##
## Conditional model:
##                          Estimate Std. Error z value Pr(>|z|)
## (Intercept)              -1.40533    0.70945  -1.981   0.0476 *
## drugMPH                  -0.10055    0.31829  -0.316   0.7521
## drugREB                   0.24884    0.29564   0.842   0.4000
## timepost-fatigue         -0.15516    0.32332  -0.480   0.6313
## drugMPH:timepost-fatigue  0.10037    0.46225   0.217   0.8281
## drugREB:timepost-fatigue -0.04784    0.43587  -0.110   0.9126
## ---
## Signif. codes:  0 '***' 0.001 '**' 0.01 '*' 0.05 '.' 0.1 ' ' 1
```

```r
report_glmmTMB_effects(model_confusion)
```

```
##                       Term Estimate_log SE_log  IRR CI_lower CI_upper p_value
## 1              (Intercept)       -1.405  0.709 0.25     0.06     0.99  0.0476
## 2                  drugMPH       -0.101  0.318 0.90     0.48     1.69  0.7520
## 3                  drugREB        0.249  0.296 1.28     0.72     2.29  0.4000
## 4         timepost-fatigue       -0.155  0.323 0.86     0.45     1.61  0.6310
## 5 drugMPH:timepost-fatigue        0.100  0.462 1.11     0.45     2.74  0.8280
## 6 drugREB:timepost-fatigue       -0.048  0.436 0.95     0.41     2.24  0.9130
```

```r
#---------------------------------------------------------CHECK ASSUMPTIONS

# Simulate residuals
sim_res2 <- simulateResiduals(model_confusion) # YES
# Plot diagnostics
plot(sim_res2) # YES
```

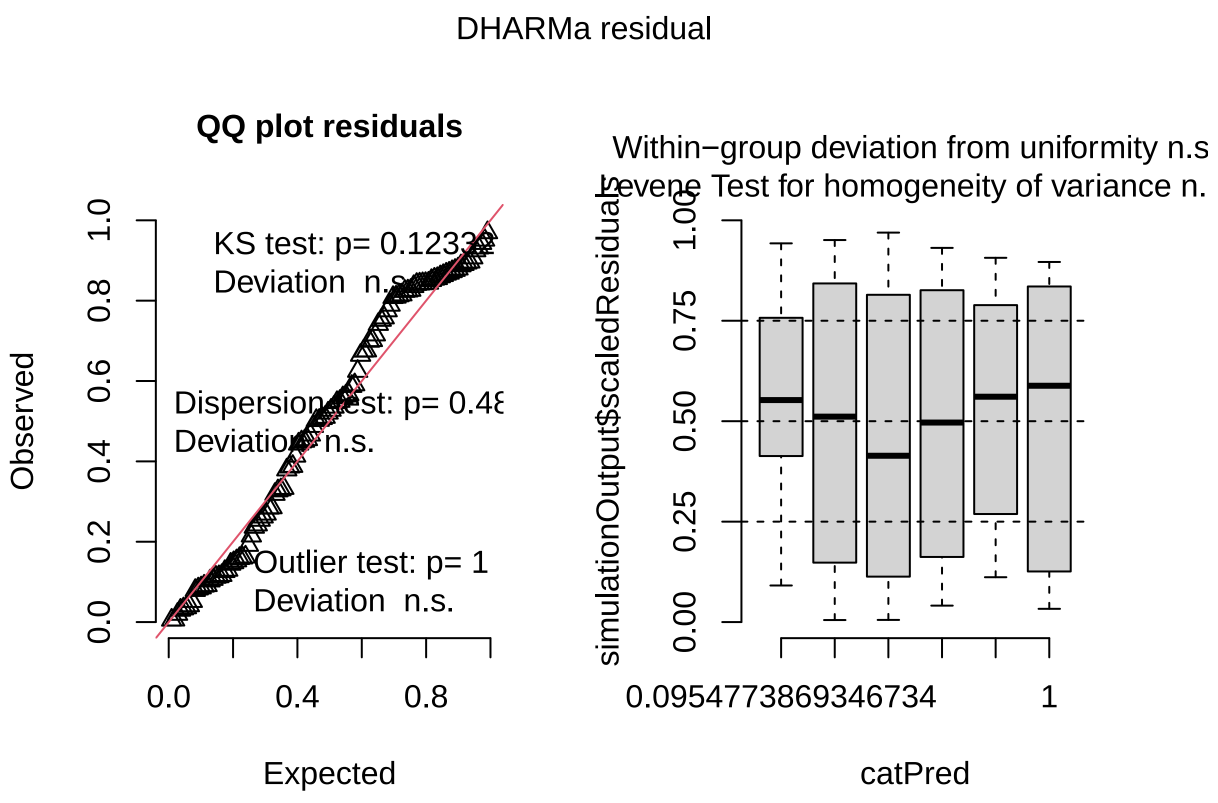


```r
# Test for overdispersion
testDispersion(sim_res2) # 0.97, p = 0.92
```

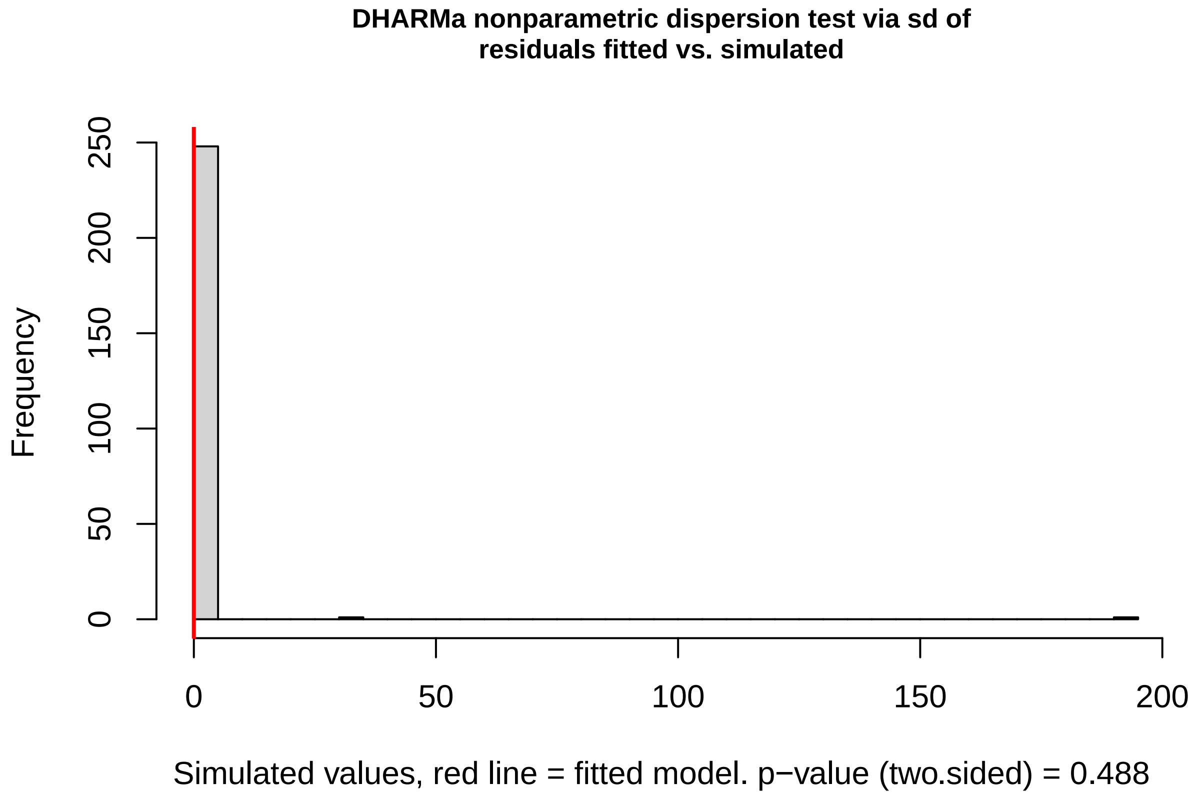


```
##
##  DHARMa nonparametric dispersion test via sd of residuals fitted vs.
##  simulated
##
## data:  simulationOutput
## dispersion = 0.0013483, p-value = 0.488
## alternative hypothesis: two.sided
```

```
testZeroInflation(sim_res2)
```

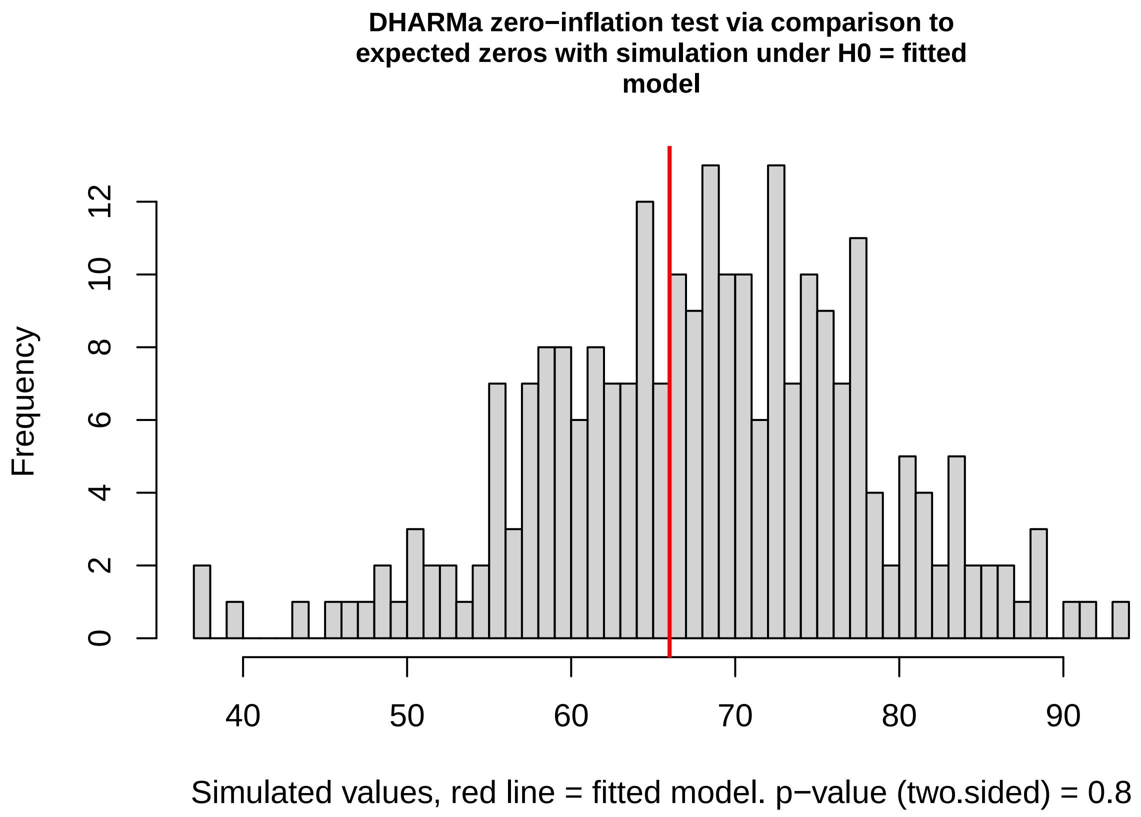


```
##
##  DHARMa zero-inflation test via comparison to expected zeros with
##  simulation under H0 = fitted model
##
## data:  simulationOutput
## ratioObsSim = 0.96204, p-value = 0.8
## alternative hypothesis: two.sided
```

4.49d DATA VISUALIZATION VIGOUR

```
##----------------------------VIGOUR
brums_vigour_plot <-
  ggplot(data_subj, aes(x = factor(time), y = BRUMS_vigour, fill = drug)) +
  geom_boxplot(alpha = 0.3, outlier.size = 2) +
  geom_jitter(aes(color = drug),
              position = position_jitterdodge(jitter.width = 0.2, dodge.width = 0.8),
              size = 1.5, alpha = 0.6) +
  stat_summary(fun = mean, geom = "point",
               position = position_dodge(width = 0.8),
               size = 3, shape = 18, aes(color = drug)) +
  labs(title = "Vigour by Time and Drug",
       x = "Time", y = "Vigour") +
  theme_minimal() +
  scale_fill_brewer(palette = "Set2") +
```

```r
  scale_color_brewer(palette = "Set2")

# Histogram
brums_vigour_hist <-
  ggplot(data_subj, aes(x = BRUMS_vigour, fill = drug)) +
  geom_histogram(binwidth = 1, position = "dodge", color = "black", alpha = 0.7) +
  facet_wrap(~ time) +
  labs(title = "Vigour Distribution by Time and Drug",
       x = "Vigour Score", y = "Count") +
  theme_minimal() +
  scale_fill_brewer(palette = "Set2")

# Combine plots
brums_vigour_plot / brums_vigour_hist + plot_layout(heights = c(2, 1))
```

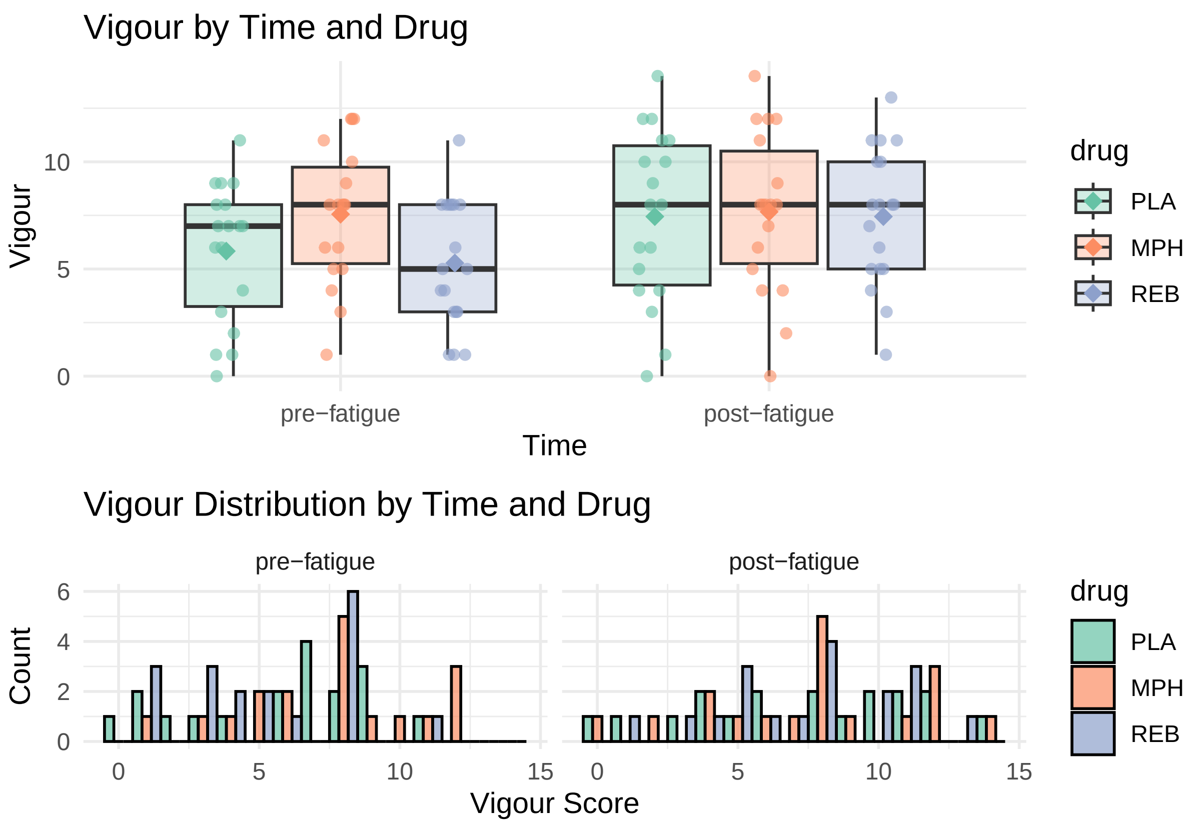

4.49e DATA ANALYSES VIGOUR

```r
model_vigour <- lmer(BRUMS_vigour ~ drug * time + (1 | record_id), data = data_subj) # the data behaves
summary(model_vigour)
```

```
## Linear mixed model fit by REML. t-tests use Satterthwaite's method [
## lmerModLmerTest]
## Formula: BRUMS_vigour ~ drug * time + (1 | record_id)
```

```
##    Data: data_subj
##
## REML criterion at convergence: 516
##
## Scaled residuals:
##      Min       1Q   Median       3Q      Max
## -2.11021 -0.75268 -0.03746  0.62372  2.30818
##
## Random effects:
##  Groups    Name        Variance Std.Dev.
##  record_id (Intercept) 6.192    2.488
##  Residual              5.529    2.351
## Number of obs: 108, groups:  record_id, 18
##
## Fixed effects:
##                            Estimate Std. Error      df t value Pr(>|t|)
## (Intercept)                  5.8333     0.8069 42.5813   7.229 6.34e-09 ***
## drugMPH                      1.7222     0.7838 85.0000   2.197   0.0307 *
## drugREB                     -0.5556     0.7838 85.0000  -0.709   0.4804
## timepost-fatigue             1.6111     0.7838 85.0000   2.056   0.0429 *
## drugMPH:timepost-fatigue  -1.5000     1.1084 85.0000  -1.353   0.1795
## drugREB:timepost-fatigue   0.5556     1.1084 85.0000   0.501   0.6175
## ---
## Signif. codes:  0 '***' 0.001 '**' 0.01 '*' 0.05 '.' 0.1 ' ' 1
##
## Correlation of Fixed Effects:
##             (Intr) drgMPH drgREB tmpst- dMPH:-
## drugMPH     -0.486
## drugREB     -0.486  0.500
## timepst-ftg -0.486  0.500  0.500
## drgMPH:tmp-  0.343 -0.707 -0.354 -0.707
## drgREB:tmp-  0.343 -0.354 -0.707 -0.707  0.500
```

```
summarize_lmer_effects(model_vigour)
```

```
##                       Term Estimate   SE Estimate_inv CI_lower CI_upper p_value
## 1              (Intercept)     5.83 0.81         5.83     4.25     7.41   0.000
## 2                  drugMPH     1.72 0.78         1.72     0.19     3.26   0.031
## 3                  drugREB    -0.56 0.78        -0.56    -2.09     0.98   0.480
## 4         timepost-fatigue     1.61 0.78         1.61     0.07     3.15   0.043
## 5 drugMPH:timepost-fatigue    -1.50 1.11        -1.50    -3.67     0.67   0.180
## 6 drugREB:timepost-fatigue     0.56 1.11         0.56    -1.62     2.73   0.618
```

```
#---------------------------------------------------------------ASSUMPTION CHECK

sim_res2 <- simulateResiduals(model_vigour) #
# Plot diagnostics
plot(sim_res2) # CHECK
```

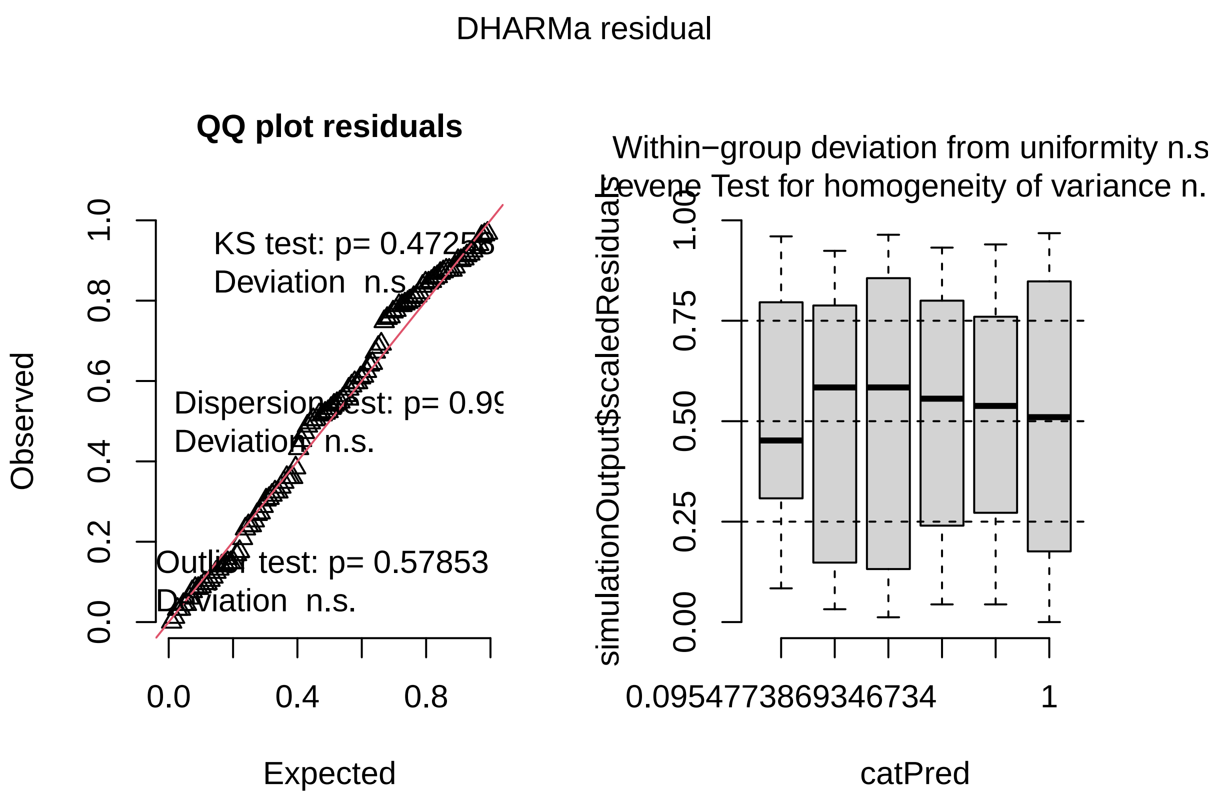


```r
##--------------------------- Gather all together in a plot for overall visualization

# Reshape the data to long format for easier plotting with ggplot
data_long_brums <- data_subj %>%
  gather(key = "subscale", value = "score",
         BRUMS_tension, BRUMS_depression, BRUMS_anger,
         BRUMS_fatigue, BRUMS_confusion, BRUMS_vigour)

# Visualize all subscales in one plot
combined_brums_plot <- ggplot(data_long_brums, aes(x = drug, y = score, fill = drug)) +
  geom_boxplot(alpha = 0.3, color = "black") +  # Boxplot with transparency
  geom_jitter(aes(color = drug),
              position = position_jitterdodge(jitter.width = 0.2, dodge.width = 0.8),
              size = 1.5, alpha = 0.6) +  # Show raw points
  labs(title = "BRUMS Subscales by Drug and Time",
       x = "Drug", y = "BRUMS Score") +
  theme_minimal() +
  scale_fill_brewer(palette = "Set2") +
  scale_color_brewer(palette = "Set2") +
  facet_wrap(~ subscale, scales = "free_y", ncol = 3) +  # Facet by subscale, free y-axis scales
  theme(legend.position = "right")

# Display the combined plot
```

```
combined_brums_plot
```

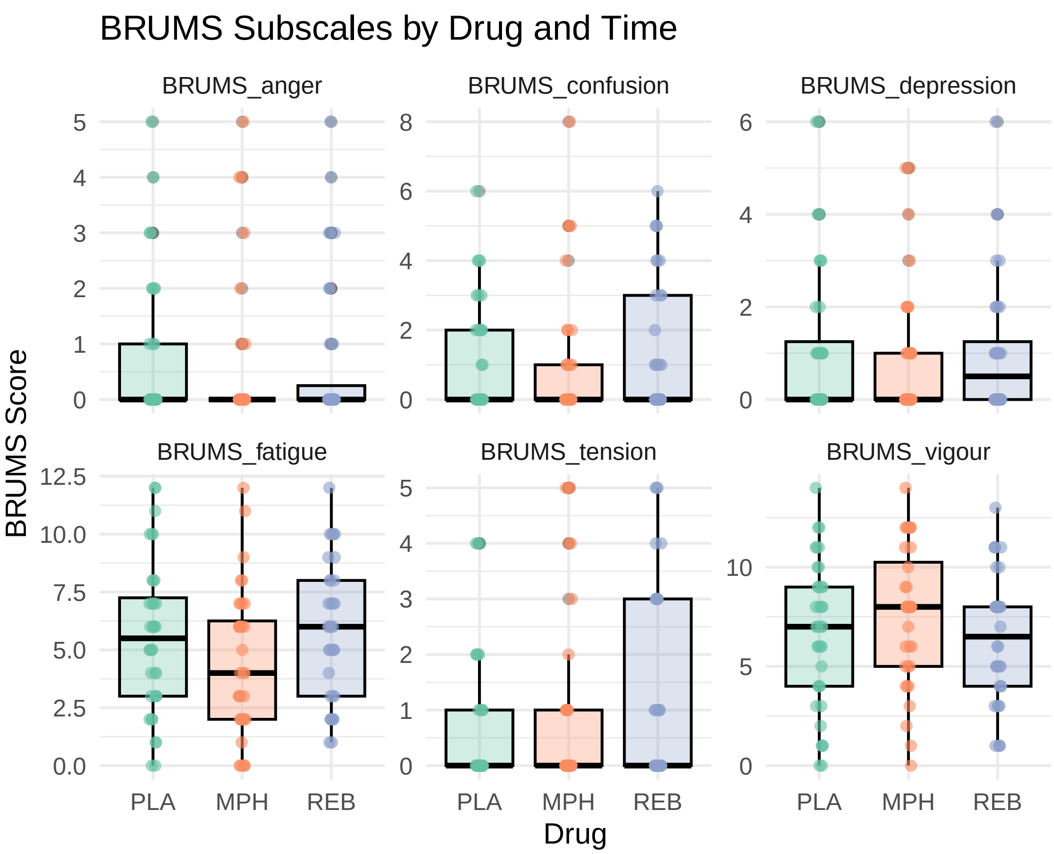

4.49f PLOT TOGETHER

4.5 SLEEPINESS

4.51 DESCRIPTIVES AND VISUALIZATION (SLEEPINESS)

```
# -------------------------------------SLEEP---------------------------------------------- ( ordinal r
# Create numeric version of KSS
data_subj$KSS_num <- as.numeric(as.character(data_subj$KSS))

# Compute descriptives
mean_data_sleep <- data_subj %>%
  group_by(drug, time) %>%
  summarise(
    mean = round(mean(KSS_num, na.rm = TRUE), 2),
    sd   = round(sd(KSS_num, na.rm = TRUE), 2),
    .groups = "drop"
  )

view(mean_data_sleep)

# Boxplot for KSS (sleepiness)
kss_plot <-
```

```r
  ggplot(data_subj, aes(x = factor(time), y = KSS_num, fill = drug)) +
  geom_boxplot(alpha = 0.3, outlier.size = 2) +  # Boxplot with transparency
  geom_jitter(aes(color = drug),
              position = position_jitterdodge(jitter.width = 0.2, dodge.width = 0.8),
              size = 1.5, alpha = 0.6) +  # Show raw points
  stat_summary(fun = mean, geom = "point",
               position = position_dodge(width = 0.8),
               size = 3, shape = 18, aes(color = drug)) +
  labs(title = "Sleepiness by Time and Drug",
       x = "Time", y = "Sleepiness (KSS)") +
  theme_minimal() +
  scale_fill_brewer(palette = "Set2") +  # Color palette for fill
  scale_color_brewer(palette = "Set2")   # Color palette for points

# Histogram for KSS (sleepiness)
kss_hist <-
  ggplot(data_subj, aes(x = KSS_num, fill = drug)) +
  geom_histogram(binwidth = 1, position = "dodge", color = "black", alpha = 0.7) +
  facet_wrap(~ time) +
  labs(title = "Sleepiness Distribution by Time and Drug",
       x = "Sleepiness Score (KSS)", y = "Count") +
  theme_minimal() +
  scale_fill_brewer(palette = "Set2")

# Combine plots
kss_plot / kss_hist + plot_layout(heights = c(2, 1))
```

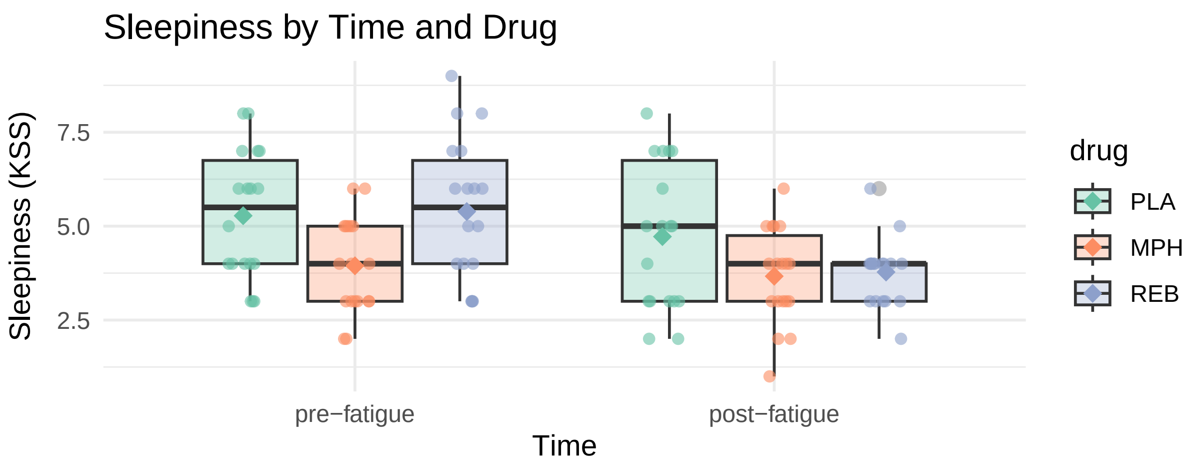

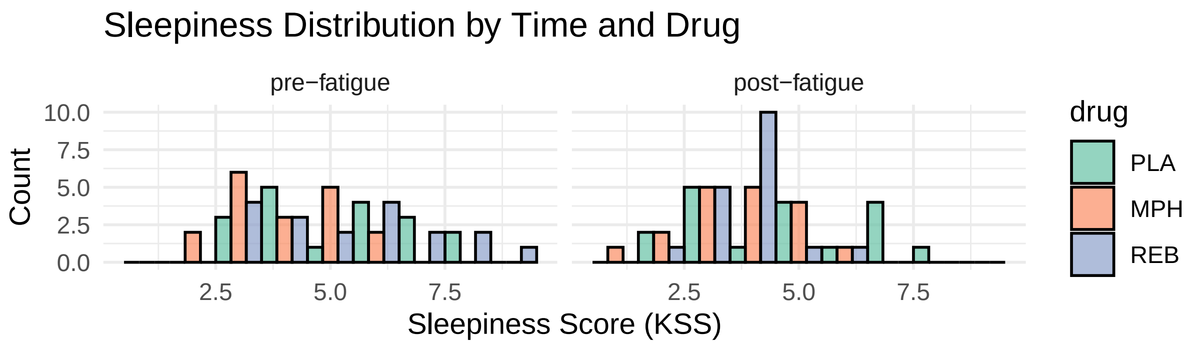

4.52 DATA ANALYSES (SLEEPINESS)

```
# Make sure KSS is a factor and ordered
data_subj$KSS <- factor(data_subj$KSS, levels = 1:9, ordered = TRUE)

# Now fit the ordinal mixed model
model_sleep_ord <- clmm(KSS ~ drug * time + (1 | record_id), data = data_subj)

# View summary
summary(model_sleep_ord)
```

```
## Cumulative Link Mixed Model fitted with the Laplace approximation
##
## formula: KSS ~ drug * time + (1 | record_id)
## data:    data_subj
##
##  link  threshold nobs logLik  AIC    niter      max.grad cond.H
##  logit flexible  108  -184.67 397.33 1011(3037) 1.74e-04 1.6e+02
##
## Random effects:
##  Groups    Name        Variance Std.Dev.
##  record_id (Intercept) 1.333    1.155
## Number of groups:  record_id 18
##
```

```
## Coefficients:
##                           Estimate Std. Error z value Pr(>|z|)
## drugMPH                    -1.7507     0.6125  -2.858  0.00426 **
## drugREB                     0.1456     0.6299   0.231  0.81725
## timepost-fatigue           -0.7733     0.6121  -1.263  0.20649
## drugMPH:timepost-fatigue    0.4146     0.8578   0.483  0.62888
## drugREB:timepost-fatigue   -1.3224     0.8793  -1.504  0.13261
## ---
## Signif. codes:  0 '***' 0.001 '**' 0.01 '*' 0.05 '.' 0.1 ' ' 1
##
## Threshold coefficients:
##     Estimate Std. Error z value
## 1|2  -6.6631     1.1743  -5.674
## 2|3  -4.4144     0.6934  -6.366
## 3|4  -2.1454     0.5590  -3.838
## 4|5  -0.7244     0.5285  -1.371
## 5|6   0.2991     0.5280   0.566
## 6|7   1.3926     0.5505   2.530
## 7|8   2.6718     0.6426   4.158
## 8|9   4.6861     1.1275   4.156
```

```
report_clmm_effects(model_sleep_ord)
```

```
##                         Term Estimate_log SE_log   OR CI_lower CI_upper  p_value
## 1                        1|2       -6.663  1.174   NA       NA       NA 1.40e-08
## 2                        2|3       -4.414  0.693   NA       NA       NA 1.94e-10
## 3                        3|4       -2.145  0.559   NA       NA       NA 1.24e-04
## 4                        4|5       -0.724  0.528   NA       NA       NA 1.70e-01
## 5                        5|6        0.299  0.528   NA       NA       NA 5.71e-01
## 6                        6|7        1.393  0.550   NA       NA       NA 1.14e-02
## 7                        7|8        2.672  0.643   NA       NA       NA 3.22e-05
## 8                        8|9        4.686  1.127   NA       NA       NA 3.23e-05
## 9                    drugMPH       -1.751  0.612 0.17     0.05     0.58 4.26e-03
## 10                   drugREB        0.146  0.630 1.16     0.34     3.98 8.17e-01
## 11          timepost-fatigue       -0.773  0.612 0.46     0.14     1.53 2.06e-01
## 12 drugMPH:timepost-fatigue        0.415  0.858 1.51     0.28     8.13 6.29e-01
## 13 drugREB:timepost-fatigue       -1.322  0.879 0.27     0.05     1.49 1.33e-01
```

```
#---------------------------------------Model assumption check
# there is no standard for Proportional odd assumption if  random effects are included so i fit the sam

# Fit a clm model (no random effects)
model_clm_ass <- clm(KSS ~ drug * time, data = data_subj)

# Test the proportional odds assumption
po_test <- nominal_test(model_clm_ass)

# View results
summary(po_test)
```

```
##        Df         logLik             AIC              LRT            Pr(>Chi)
##  Min.   :7   Min.   :-192.3   Min.   :410.7   Min.   :5.904   Min.   :0.551
##  1st Qu.:7   1st Qu.:-191.6   1st Qu.:412.7   1st Qu.:5.904   1st Qu.:0.551
```

```
## Median :7   Median :-190.9  Median :414.7  Median :5.904  Median :0.551
## Mean   :7   Mean   :-190.9  Mean   :414.7  Mean   :5.904  Mean   :0.551
## 3rd Qu.:7   3rd Qu.:-190.1  3rd Qu.:416.8  3rd Qu.:5.904  3rd Qu.:0.551
## Max.   :7   Max.   :-189.4  Max.   :418.8  Max.   :5.904  Max.   :0.551
## NA's   :3   NA's   :2       NA's   :2      NA's   :3      NA's   :3
```

```
# effects are not violated. p>0.05 --> all good
```

4.6 INTERNAL WORKLOAD

4.61 DATA descriptives internal workload

```
# --------------------------------------INTERNAL WORKLOAD  -----------------------------------
# Subscales of mental, physical, temporal, performance, effort and frustration and one averaged datapoi

# Compute descriptives of all subscales
mean_data_NASA <- data_perf %>%
  group_by(drug) %>%
  summarise(
    mental_mean = round(mean(NASA_Mental, na.rm = TRUE), 2),
    mental_sd = round(sd(NASA_Mental, na.rm = TRUE), 2),
    physical_mean = round(mean(NASA_Physical, na.rm = TRUE), 2),
    physical_sd = round(sd(NASA_Physical, na.rm = TRUE), 2),
    temporal_mean = round(mean(NASA_Temporal, na.rm = TRUE), 2),
    temporal_sd = round(sd(NASA_Temporal, na.rm = TRUE), 2),
    performance_mean = round(mean(NASA_Performance, na.rm = TRUE), 2),
    performance_sd = round(sd(NASA_Performance, na.rm = TRUE), 2),
    effort_mean = round(mean(NASA_Effort, na.rm = TRUE), 2),
    effort_sd = round(sd(NASA_Effort, na.rm = TRUE), 2),
    frustration_mean = round(mean(NASA_Frustration, na.rm = TRUE), 2),
    frustration_sd = round(sd(NASA_Frustration, na.rm = TRUE), 2),
    average_mean = round(mean(NASA_average, na.rm = TRUE), 2),
    average_sd = round(sd(NASA_average, na.rm = TRUE), 2)
  )

view(mean_data_NASA )
```

4.62 DATA VISUALIZATION AVERAGED WORKLOAD

```
# Visualize by boxplot
## ------------------------ AVERAGED
# Boxplot for NASA_average (Workload)
nasa_workload_plot <-
  ggplot(data_perf, aes(x = drug, y = NASA_average, fill = drug)) +
  geom_jitter(aes(color = drug),
              position = position_jitterdodge(jitter.width = 0.2, dodge.width = 0.8),
              size = 1.5, alpha = 0.6) +  # Show raw points
  geom_boxplot(aes(fill = drug), color = "firebrick", alpha = 0.3) +  # Boxplot with fill based on drug
  labs(title = "Averaged Workload per Drug",
       x = "Drug", y = "Workload (NASA Average)") +
```

```
  theme_minimal() +
  scale_fill_brewer(palette = "Set2") +  # Color palette for boxplot fill
  scale_color_brewer(palette = "Set2")   # Color palette for jittered points

# Histogram for NASA_average (Workload)
nasa_workload_hist <-
  ggplot(data_perf, aes(x = NASA_average, fill = drug)) +
  geom_histogram(binwidth = 1, position = "dodge", color = "black", alpha = 0.7) +
  facet_wrap(~ drug) +  # Facet by drug condition
  labs(title = "Workload Distribution by Drug",
       x = "Workload Score (NASA Average)", y = "Count") +
  theme_minimal() +
  scale_fill_brewer(palette = "Set2")  # Consistent color palette for the histogram

# Combine plots
nasa_workload_plot / nasa_workload_hist + plot_layout(heights = c(2, 1))
```

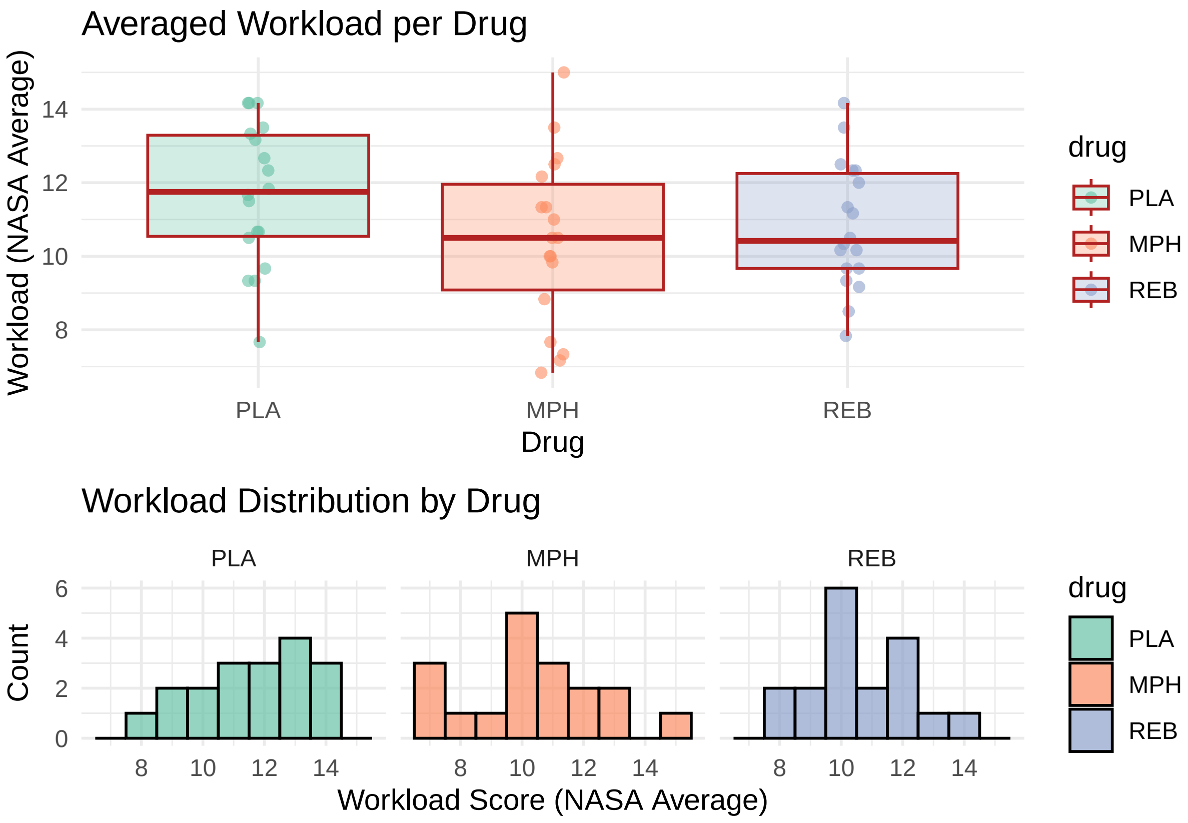

4.63 DATA ANALYSIS AVERAGED WORKLOAD

```
model_NASA <- lmer(NASA_average ~ drug + (1 | record_id), data = data_perf) # the data behaves normal s
summary(model_NASA)
```

```
## Linear mixed model fit by REML. t-tests use Satterthwaite's method [
```

```
## lmerModLmerTest]
## Formula: NASA_average ~ drug + (1 | record_id)
##    Data: data_perf
##
## REML criterion at convergence: 202.9
##
## Scaled residuals:
##     Min      1Q  Median      3Q     Max
## -1.5618 -0.5874 -0.1381  0.6525  1.9507
##
## Random effects:
##  Groups    Name        Variance Std.Dev.
##  record_id (Intercept) 2.540    1.594
##  Residual              1.424    1.193
## Number of obs: 54, groups:  record_id, 18
##
## Fixed effects:
##             Estimate Std. Error      df t value Pr(>|t|)
## (Intercept)  11.6852     0.4693 28.0042  24.898  < 2e-16 ***
## drugMPH      -1.2315     0.3978 34.0000  -3.096  0.00392 **
## drugREB      -0.8704     0.3978 34.0000  -2.188  0.03565 *
## ---
## Signif. codes:  0 '***' 0.001 '**' 0.01 '*' 0.05 '.' 0.1 ' ' 1
##
## Correlation of Fixed Effects:
##         (Intr) drgMPH
## drugMPH -0.424
## drugREB -0.424  0.500
```

```
data_perf$NASA_average_log <- log(data_perf$NASA_average + 1)
model_NASA_log <- lmer(NASA_average_log ~ drug + (1 | record_id), data = data_perf)
summary(model_NASA_log)
```

```
## Linear mixed model fit by REML. t-tests use Satterthwaite's method [
## lmerModLmerTest]
## Formula: NASA_average_log ~ drug + (1 | record_id)
##    Data: data_perf
##
## REML criterion at convergence: -47.5
##
## Scaled residuals:
##     Min      1Q  Median      3Q     Max
## -1.7704 -0.5622 -0.0679  0.6308  1.7379
##
## Random effects:
##  Groups    Name        Variance Std.Dev.
##  record_id (Intercept) 0.01923  0.1387
##  Residual              0.01042  0.1021
## Number of obs: 54, groups:  record_id, 18
##
## Fixed effects:
##             Estimate Std. Error       df t value Pr(>|t|)
## (Intercept)  2.52889    0.04058 27.69776  62.313  < 2e-16 ***
## drugMPH     -0.11008    0.03402 34.00000  -3.235  0.00271 **
```

```
## drugREB     -0.06962    0.03402 34.00000  -2.046  0.04854 *
## ---
## Signif. codes:  0 '***' 0.001 '**' 0.01 '*' 0.05 '.' 0.1 ' ' 1
##
## Correlation of Fixed Effects:
##         (Intr) drgMPH
## drugMPH -0.419
## drugREB -0.419  0.500
```

```
summary_log <- summarize_lmer_effects(model_NASA_log, inverse = exp)


anova(model_NASA, model_NASA_log) # model 2 is better! AIC and BIC improved considerably
```

```
## refitting model(s) with ML (instead of REML)

## Data: data_perf
## Models:
## model_NASA: NASA_average ~ drug + (1 | record_id)
## model_NASA_log: NASA_average_log ~ drug + (1 | record_id)
##                npar     AIC     BIC   logLik deviance  Chisq Df Pr(>Chisq)
## model_NASA        5 212.531 222.476 -101.266  202.531
## model_NASA_log    5 -52.512 -42.567   31.256  -62.512 265.04  0
```

4.64 DATA VISUALIZATION OF ALL OTHER SUBSCALES

```
##--------------------------- MENTAL
# Boxplot for NASA_Mental (Mental Workload)
nasa_mental_plot <-
  ggplot(data_perf, aes(x = drug, y = NASA_Mental, fill = drug)) +
  geom_jitter(aes(color = drug),
              position = position_jitterdodge(jitter.width = 0.2, dodge.width = 0.8),
              size = 1.5, alpha = 0.6) +  # Show raw points
  geom_boxplot(aes(fill = drug), color = "darkorange", alpha = 0.3) +  # Boxplot with fill based on dru
  labs(title = "Mental Workload per Drug",
       x = "Drug", y = "Workload (NASA Mental)") +
  theme_minimal() +
  scale_fill_brewer(palette = "Set1") +  # Color palette for boxplot fill
  scale_color_brewer(palette = "Set1")   # Color palette for jittered points

# Histogram for NASA_Mental (Mental Workload)
nasa_mental_hist <-
  ggplot(data_perf, aes(x = NASA_Mental, fill = drug)) +
  geom_histogram(binwidth = 1, position = "dodge", color = "black", alpha = 0.7) +
  facet_wrap(~ drug) +  # Facet by drug condition
  labs(title = "Mental Workload Distribution by Drug",
       x = "Mental Workload Score (NASA Mental)", y = "Count") +
  theme_minimal() +
  scale_fill_brewer(palette = "Set1")  # Consistent color palette for the histogram

# Combine plots
nasa_mental_plot / nasa_mental_hist + plot_layout(heights = c(2, 1))
```

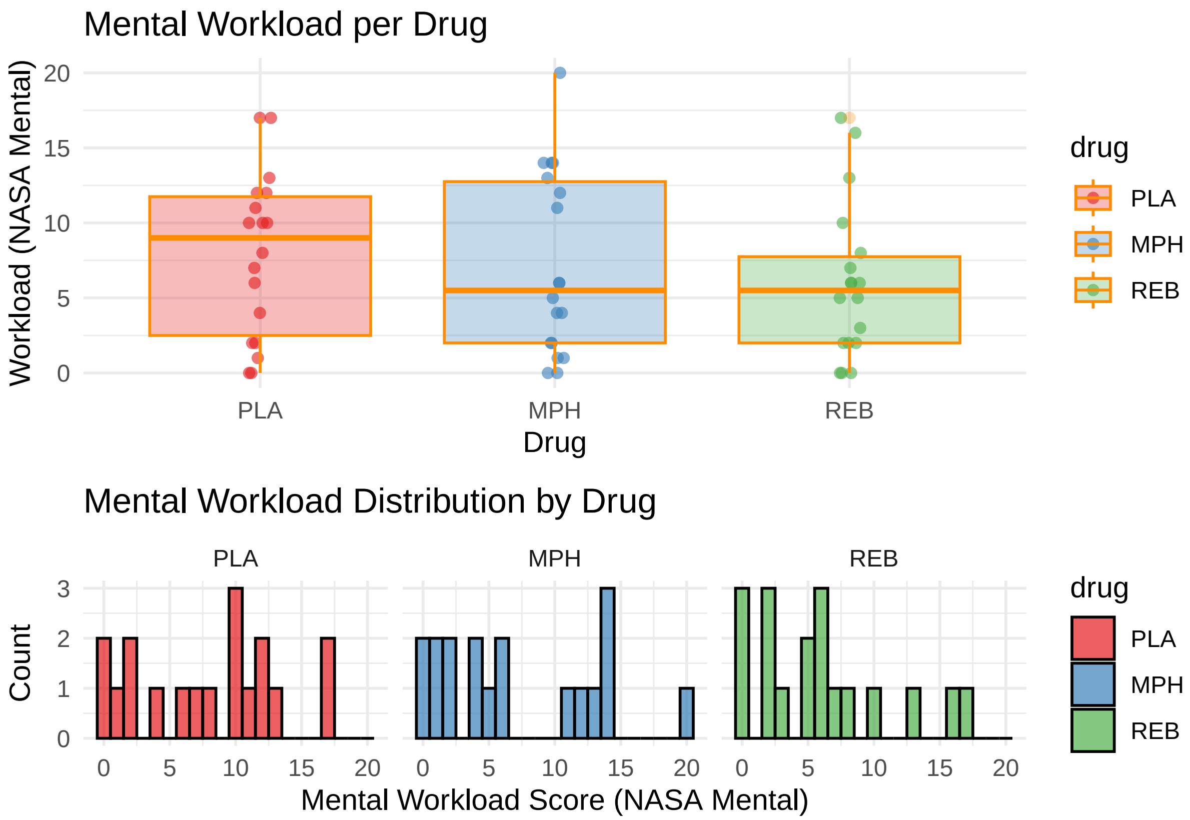


```r
## ---------------------------PHYSICAL
# Boxplot for NASA_Physical (Physical Workload)
nasa_physical_plot <-
  ggplot(data_perf, aes(x = drug, y = NASA_Physical, fill = drug)) +
  geom_jitter(aes(color = drug),
              position = position_jitterdodge(jitter.width = 0.2, dodge.width = 0.8),
              size = 1.5, alpha = 0.6) +  # Show raw points
  geom_boxplot(aes(fill = drug), color = "purple", alpha = 0.3) +  # Boxplot with fill based on drug
  labs(title = "Physical Workload per Drug",
       x = "Drug", y = "Physical Workload (NASA Physical)") +
  theme_minimal() +
  scale_fill_brewer(palette = "Set3") +  # Color palette for boxplot fill
  scale_color_brewer(palette = "Set3")   # Color palette for jittered points

# Histogram for NASA_Physical (Physical Workload)
nasa_physical_hist <-
  ggplot(data_perf, aes(x = NASA_Physical, fill = drug)) +
  geom_histogram(binwidth = 1, position = "dodge", color = "black", alpha = 0.7) +
  facet_wrap(~ drug) +  # Facet by drug condition
  labs(title = "Physical Workload Distribution by Drug",
       x = "Physical Workload Score (NASA Physical)", y = "Count") +
  theme_minimal() +
  scale_fill_brewer(palette = "Set3")  # Consistent color palette for the histogram

# Combine plots
nasa_physical_plot / nasa_physical_hist + plot_layout(heights = c(2, 1))
```

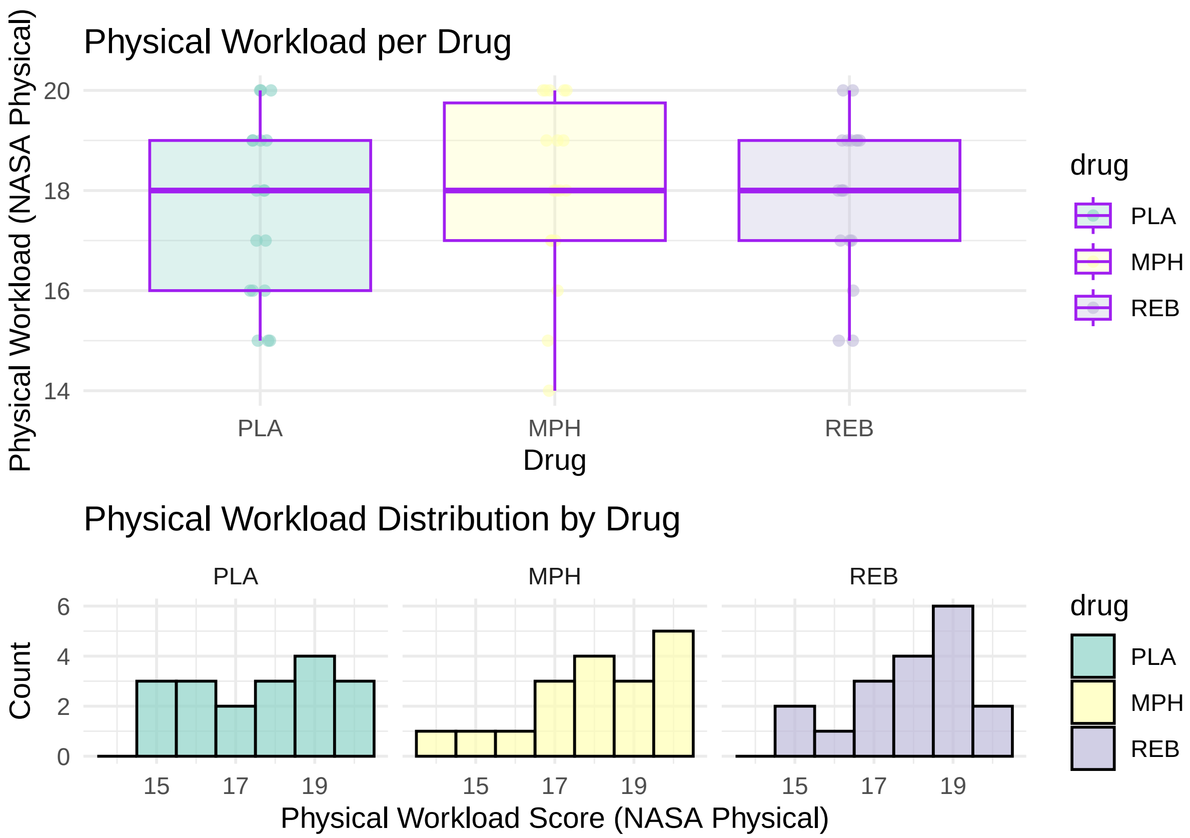


```
##--------------------------- TEMPORAL
# Boxplot for NASA_Temporal (Temporal Workload)
nasa_temporal_plot <-
  ggplot(data_perf, aes(x = drug, y = NASA_Temporal, fill = drug)) +
  geom_jitter(aes(color = drug),
              position = position_jitterdodge(jitter.width = 0.2, dodge.width = 0.8),
              size = 1.5, alpha = 0.6) +  # Show raw points
  geom_boxplot(aes(fill = drug), color = "midnightblue", alpha = 0.3) +  # Boxplot with fill based on d
  labs(title = "Temporal Workload per Drug",
       x = "Drug", y = "Workload (NASA Temporal)") +
  theme_minimal() +
  scale_fill_brewer(palette = "Set2") +  # Color palette for boxplot fill
  scale_color_brewer(palette = "Set2")   # Color palette for jittered points

# Histogram for NASA_Temporal (Temporal Workload)
nasa_temporal_hist <-
  ggplot(data_perf, aes(x = NASA_Temporal, fill = drug)) +
  geom_histogram(binwidth = 1, position = "dodge", color = "black", alpha = 0.7) +
  facet_wrap(~ drug) +  # Facet by drug condition
  labs(title = "Temporal Workload Distribution by Drug",
       x = "Temporal Workload Score (NASA Temporal)", y = "Count") +
  theme_minimal() +
  scale_fill_brewer(palette = "Set2")  # Consistent color palette for the histogram

# Combine plots
nasa_temporal_plot / nasa_temporal_hist + plot_layout(heights = c(2, 1))
```

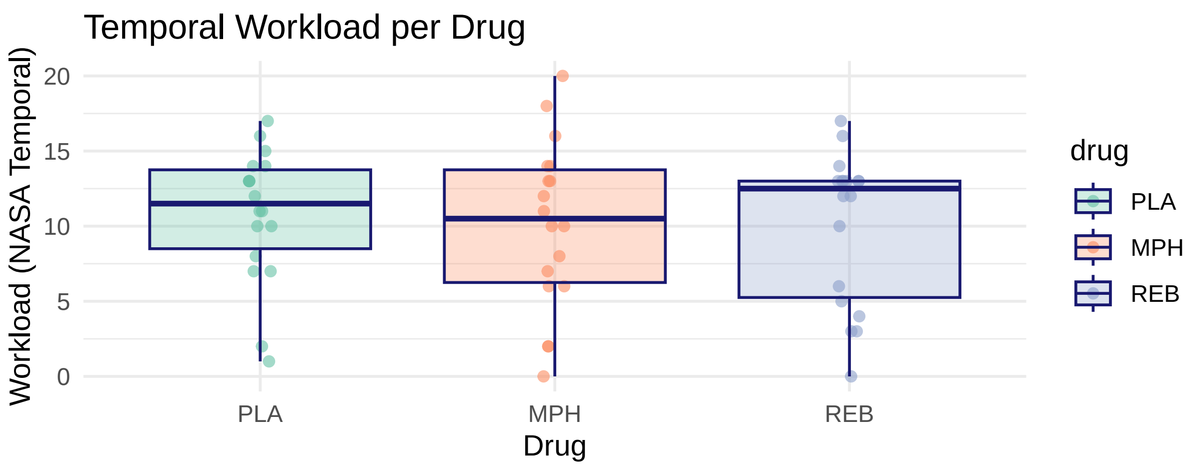

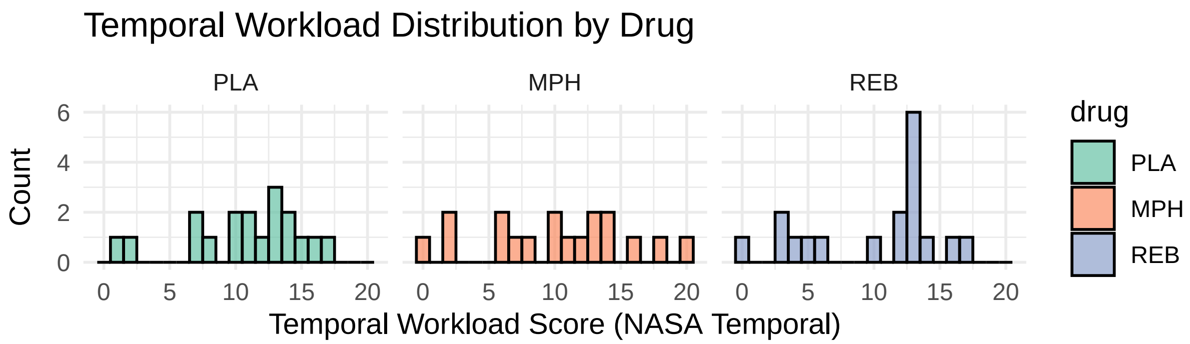


```
##---------------------------- PERFORMANCE
# Boxplot for NASA_Performance (Performance Workload)
nasa_performance_plot <-
  ggplot(data_perf, aes(x = drug, y = NASA_Performance, fill = drug)) +
  geom_jitter(aes(color = drug),
              position = position_jitterdodge(jitter.width = 0.2, dodge.width = 0.8),
              size = 1.5, alpha = 0.6) +  # Show raw points
  geom_boxplot(aes(fill = drug), color = "seagreen", alpha = 0.3) +  # Boxplot with fill based on drug
  labs(title = "Performance Workload per Drug",
       x = "Drug", y = "Workload (NASA Performance)") +
  theme_minimal() +
  scale_fill_brewer(palette = "Set3") +  # Color palette for boxplot fill
  scale_color_brewer(palette = "Set3")   # Color palette for jittered points

# Histogram for NASA_Performance (Performance Workload)
nasa_performance_hist <-
  ggplot(data_perf, aes(x = NASA_Performance, fill = drug)) +
  geom_histogram(binwidth = 1, position = "dodge", color = "black", alpha = 0.7) +
  facet_wrap(~ drug) +  # Facet by drug condition
  labs(title = "Performance Workload Distribution by Drug",
       x = "Performance Workload Score (NASA Performance)", y = "Count") +
  theme_minimal() +
  scale_fill_brewer(palette = "Set3")  # Consistent color palette for the histogram

# Combine plots
nasa_performance_plot / nasa_performance_hist + plot_layout(heights = c(2, 1))
```

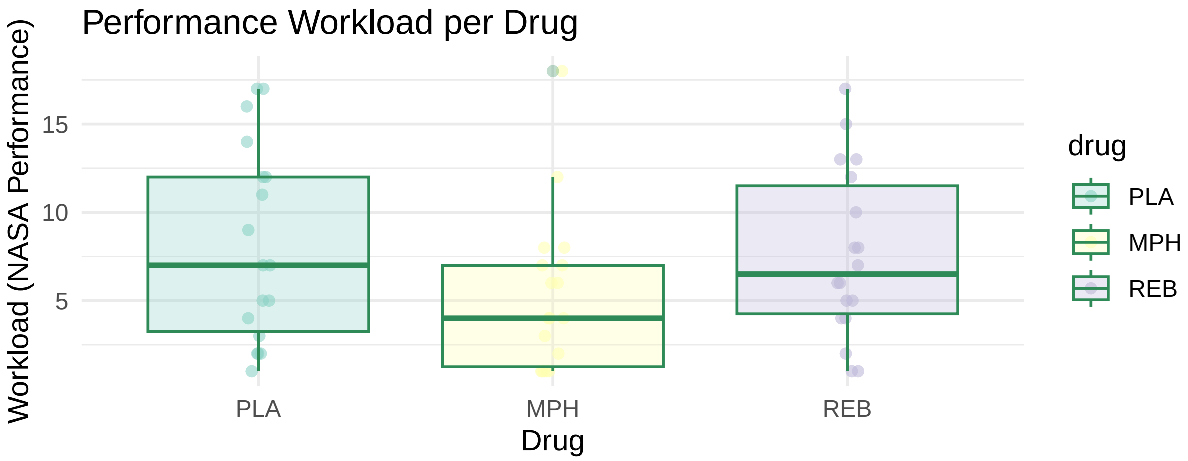

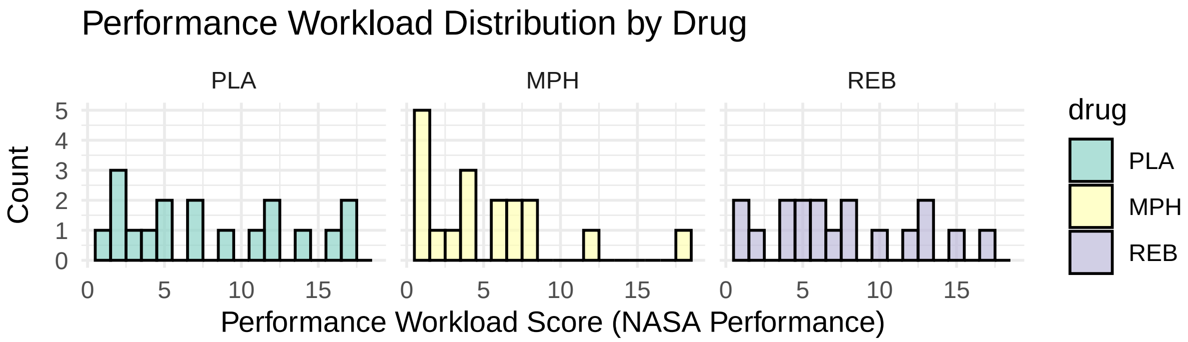


```
##----------------------------EFFORT

# Boxplot for NASA_Effort (Effort Workload)
nasa_effort_plot <-
  ggplot(data_perf, aes(x = drug, y = NASA_Effort, fill = drug)) +
  geom_jitter(aes(color = drug),
              position = position_jitterdodge(jitter.width = 0.2, dodge.width = 0.8),
              size = 1.5, alpha = 0.6) +  # Show raw points
  geom_boxplot(aes(fill = drug), color = "slateblue", alpha = 0.3) +  # Boxplot with fill based on drug
  labs(title = "Effort Workload per Drug",
       x = "Drug", y = "Workload (NASA Effort)") +
  theme_minimal() +
  scale_fill_brewer(palette = "Set1") +  # Color palette for boxplot fill
  scale_color_brewer(palette = "Set1")   # Color palette for jittered points

# Histogram for NASA_Effort (Effort Workload)
nasa_effort_hist <-
  ggplot(data_perf, aes(x = NASA_Effort, fill = drug)) +
  geom_histogram(binwidth = 1, position = "dodge", color = "black", alpha = 0.7) +
  facet_wrap(~ drug) +  # Facet by drug condition
  labs(title = "Effort Workload Distribution by Drug",
       x = "Effort Workload Score (NASA Effort)", y = "Count") +
  theme_minimal() +
  scale_fill_brewer(palette = "Set1")  # Consistent color palette for the histogram

# Combine plots
```

```
nasa_effort_plot / nasa_effort_hist + plot_layout(heights = c(2, 1))
```

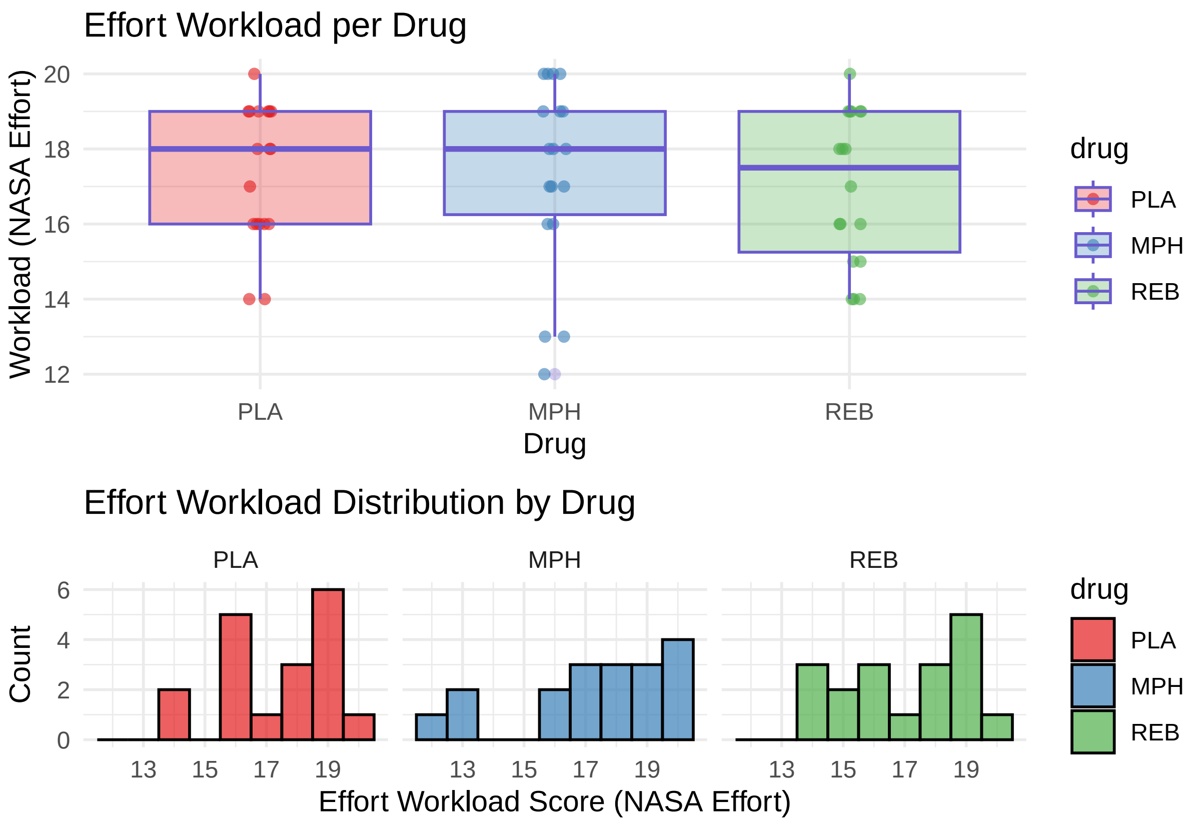


```
##--------------------------- fRUSTRATION

# Boxplot for NASA_Frustration (Frustration Workload)
nasa_frustration_plot <-
  ggplot(data_perf, aes(x = drug, y = NASA_Frustration, fill = drug)) +
  geom_jitter(aes(color = drug),
              position = position_jitterdodge(jitter.width = 0.2, dodge.width = 0.8),
              size = 1.5, alpha = 0.6) +  # Show raw points
  geom_boxplot(aes(fill = drug), color = "saddlebrown", alpha = 0.3) +  # Boxplot with fill based on dr
  labs(title = "Frustration Workload per Drug",
       x = "Drug", y = "Workload (NASA Frustration)") +
  theme_minimal() +
  scale_fill_brewer(palette = "Set2") +  # Color palette for boxplot fill
  scale_color_brewer(palette = "Set2")   # Color palette for jittered points

# Histogram for NASA_Frustration (Frustration Workload)
nasa_frustration_hist <-
  ggplot(data_perf, aes(x = NASA_Frustration, fill = drug)) +
  geom_histogram(binwidth = 1, position = "dodge", color = "black", alpha = 0.7) +
  facet_wrap(~ drug) +  # Facet by drug condition
  labs(title = "Frustration Workload Distribution by Drug",
       x = "Frustration Workload Score (NASA Frustration)", y = "Count") +
  theme_minimal() +
```

```r
  scale_fill_brewer(palette = "Set2")  # Consistent color palette for the histogram

# Combine plots
nasa_frustration_plot / nasa_frustration_hist + plot_layout(heights = c(2, 1))
```

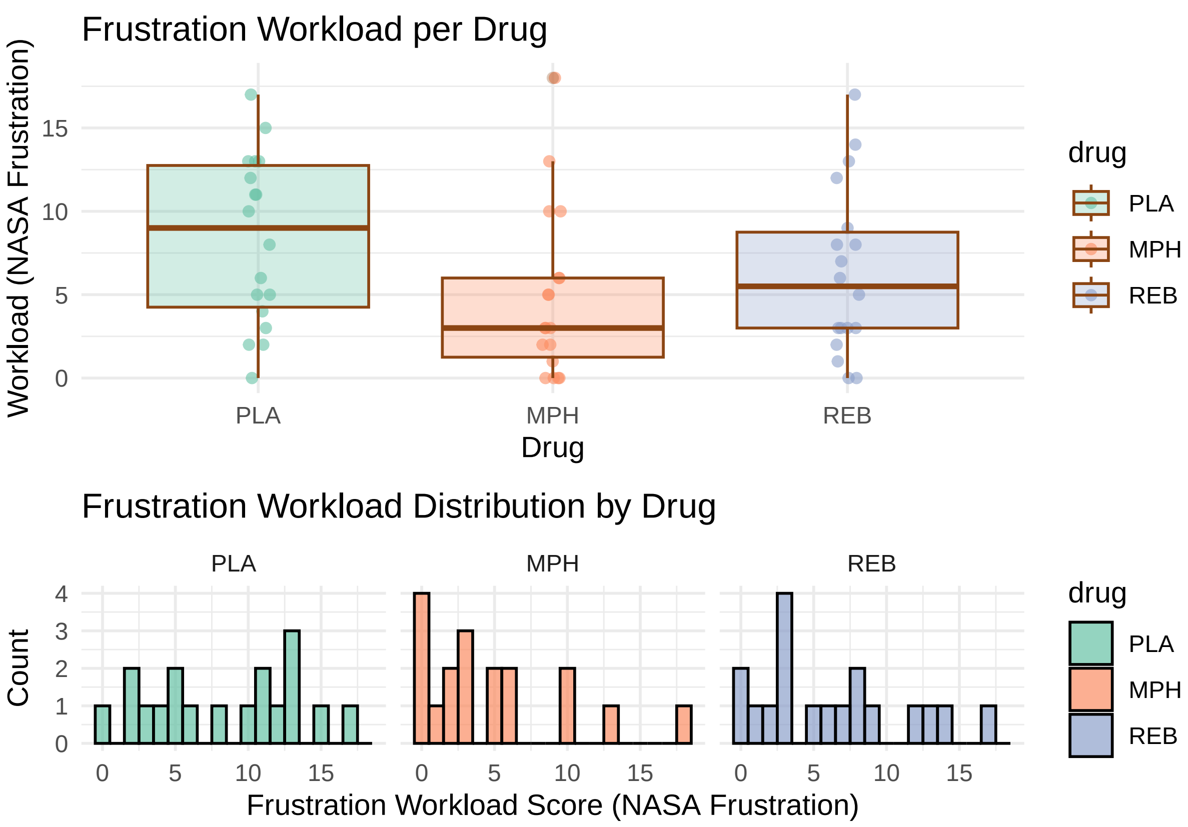

4.65 DATA ANALYSIS of all other subscales of NASATLX

```r
# orndial data from a scale of 0-20.

data_perf$NASA_Mental <- factor(data_perf$NASA_Mental, levels = 1:20, ordered = TRUE)
model_NASA_Mental <- clmm(NASA_Mental ~ drug + (1 | record_id), data = data_perf)
```

```
## Warning in update.uC(rho): Non finite negative log-likelihood
##   at iteration 155
```

```r
summary(model_NASA_Mental)
```

```
## Cumulative Link Mixed Model fitted with the Laplace approximation
##
## formula: NASA_Mental ~ drug + (1 | record_id)
## data:    data_perf
##
```

```
##  link  threshold nobs logLik  AIC    niter      max.grad cond.H
##  logit flexible  47   -112.05 260.09 1604(9984) 8.03e-05 8.0e+02
##
## Random effects:
##  Groups    Name        Variance Std.Dev.
##  record_id (Intercept) 10.78    3.283
## Number of groups:  record_id 17
##
## Coefficients:
##         Estimate Std. Error z value Pr(>|z|)
## drugMPH  -0.4207     0.6950  -0.605    0.545
## drugREB  -0.8933     0.6849  -1.304    0.192
##
## Threshold coefficients:
##       Estimate Std. Error z value
## 1|2    -5.8721     1.6543  -3.550
## 2|3    -2.6611     1.0951  -2.430
## 3|4    -2.3494     1.0734  -2.189
## 4|5    -1.6298     1.0441  -1.561
## 5|6    -1.0822     1.0313  -1.049
## 6|7    -0.1216     1.0140  -0.120
## 7|8     0.2064     1.0104   0.204
## 8|10    0.5553     1.0102   0.550
## 10|11   1.3091     1.0291   1.272
## 11|12   1.7353     1.0535   1.647
## 12|13   2.4270     1.1097   2.187
## 13|14   3.2165     1.1887   2.706
## 14|16   4.4179     1.3574   3.255
## 16|17   5.0018     1.4563   3.435
## 17|20   7.3148     1.8874   3.876
## (7 observations deleted due to missingness)
```

```r
report_clmm_effects(model_NASA_Mental)
```

```
##       Term Estimate_log SE_log   OR CI_lower CI_upper  p_value
## 1      1|2       -5.872  1.654   NA       NA       NA 0.000386
## 2      2|3       -2.661  1.095   NA       NA       NA 0.015100
## 3      3|4       -2.349  1.073   NA       NA       NA 0.028600
## 4      4|5       -1.630  1.044   NA       NA       NA 0.119000
## 5      5|6       -1.082  1.031   NA       NA       NA 0.294000
## 6      6|7       -0.122  1.014   NA       NA       NA 0.905000
## 7      7|8        0.206  1.010   NA       NA       NA 0.838000
## 8     8|10        0.555  1.010   NA       NA       NA 0.583000
## 9    10|11        1.309  1.029   NA       NA       NA 0.203000
## 10   11|12        1.735  1.054   NA       NA       NA 0.099500
## 11   12|13        2.427  1.110   NA       NA       NA 0.028700
## 12   13|14        3.217  1.189   NA       NA       NA 0.006810
## 13   14|16        4.418  1.357   NA       NA       NA 0.001140
## 14   16|17        5.002  1.456   NA       NA       NA 0.000594
## 15   17|20        7.315  1.887   NA       NA       NA 0.000106
## 16 drugMPH       -0.421  0.695 0.66     0.17     2.56 0.545000
## 17 drugREB       -0.893  0.685 0.41     0.11     1.57 0.192000
```

```r
data_perf$NASA_Physical <- factor(data_perf$NASA_Physical, levels = 1:20, ordered = TRUE)
model_NASA_Physical <- clmm(NASA_Physical ~ drug + (1 | record_id), data = data_perf) # the data behave
summary(model_NASA_Physical)
```

```
## Cumulative Link Mixed Model fitted with the Laplace approximation
##
## formula: NASA_Physical ~ drug + (1 | record_id)
## data:    data_perf
##
##  link  threshold nobs logLik AIC    niter     max.grad cond.H
##  logit flexible  54   -94.27 206.53 439(1320) 5.29e-06 6.7e+01
##
## Random effects:
##  Groups    Name        Variance Std.Dev.
##  record_id (Intercept) 1.584    1.259
## Number of groups:  record_id 18
##
## Coefficients:
##         Estimate Std. Error z value Pr(>|z|)
## drugMPH   0.5398     0.6203   0.870    0.384
## drugREB   0.4564     0.6132   0.744    0.457
##
## Threshold coefficients:
##       Estimate Std. Error z value
## 14|15  -4.4649     1.1872  -3.761
## 15|16  -2.1347     0.6648  -3.211
## 16|17  -1.3093     0.5998  -2.183
## 17|18  -0.3661     0.5687  -0.644
## 18|19   0.7421     0.5746   1.291
## 19|20   2.2595     0.6610   3.418
```

```r
report_clmm_effects(model_NASA_Physical)
```

```
##      Term Estimate_log SE_log   OR CI_lower CI_upper  p_value
## 1   14|15       -4.465  1.187   NA       NA       NA 0.000169
## 2   15|16       -2.135  0.665   NA       NA       NA 0.001320
## 3   16|17       -1.309  0.600   NA       NA       NA 0.029000
## 4   17|18       -0.366  0.569   NA       NA       NA 0.520000
## 5   18|19        0.742  0.575   NA       NA       NA 0.197000
## 6   19|20        2.260  0.661   NA       NA       NA 0.000630
## 7 drugMPH        0.540  0.620 1.72     0.51     5.79 0.384000
## 8 drugREB        0.456  0.613 1.58     0.47     5.25 0.457000
```

```r
data_perf$NASA_Temporal <- factor(data_perf$NASA_Temporal, levels = 1:20, ordered = TRUE)
model_NASA_Temporal <- clmm(NASA_Temporal ~ drug + (1 | record_id), data = data_perf) # the data behave
summary(model_NASA_Temporal)
```

```
## Cumulative Link Mixed Model fitted with the Laplace approximation
##
## formula: NASA_Temporal ~ drug + (1 | record_id)
## data:    data_perf
```

```
##
##  link  threshold nobs logLik  AIC    niter       max.grad cond.H
##  logit flexible  52   -131.17 302.35 2891(14050) 8.06e+00 5.1e+05
##
## Random effects:
##  Groups    Name        Variance Std.Dev.
##  record_id (Intercept) 4.036    2.009
## Number of groups:  record_id 18
##
## Coefficients:
##          Estimate Std. Error z value Pr(>|z|)
## drugMPH -0.331792   0.002497 -132.89   <2e-16 ***
## drugREB -0.133497   0.002497  -53.46   <2e-16 ***
## ---
## Signif. codes:  0 '***' 0.001 '**' 0.01 '*' 0.05 '.' 0.1 ' ' 1
##
## Threshold coefficients:
##        Estimate Std. Error  z value
## 1|2   -5.696341   1.230811   -4.628
## 2|3   -3.840827   0.776290   -4.948
## 3|4   -3.223101   0.693579   -4.647
## 4|5   -2.969469   0.665580   -4.461
## 5|6   -2.730660   0.641252   -4.258
## 6|7   -2.148862   0.594652   -3.614
## 7|8   -1.639895   0.560669   -2.925
## 8|10  -1.309234   0.539769   -2.426
## 10|11 -0.526688   0.492654   -1.069
## 11|12 -0.100448   0.469843   -0.214
## 12|13  0.425088   0.444269    0.957
## 13|14  1.925585   0.341862    5.633
## 14|15  2.812014   0.002495 1126.845
## 15|16  3.022217   0.002495 1211.244
## 16|17  3.789694   0.002497 1517.454
## 17|18  4.589315   0.002497 1837.924
## 18|20  5.295791   0.716658    7.390
## (2 observations deleted due to missingness)
```

```
report_clmm_effects(model_NASA_Temporal)
```

```
##       Term Estimate_log SE_log   OR CI_lower CI_upper  p_value
## 1      1|2       -5.696  1.231   NA       NA       NA 3.69e-06
## 2      2|3       -3.841  0.776   NA       NA       NA 7.51e-07
## 3      3|4       -3.223  0.694   NA       NA       NA 3.37e-06
## 4      4|5       -2.969  0.666   NA       NA       NA 8.14e-06
## 5      5|6       -2.731  0.641   NA       NA       NA 2.06e-05
## 6      6|7       -2.149  0.595   NA       NA       NA 3.02e-04
## 7      7|8       -1.640  0.561   NA       NA       NA 3.45e-03
## 8     8|10       -1.309  0.540   NA       NA       NA 1.53e-02
## 9    10|11       -0.527  0.493   NA       NA       NA 2.85e-01
## 10   11|12       -0.100  0.470   NA       NA       NA 8.31e-01
## 11   12|13        0.425  0.444   NA       NA       NA 3.39e-01
## 12   13|14        1.926  0.342   NA       NA       NA 1.77e-08
## 13   14|15        2.812  0.002   NA       NA       NA 0.00e+00
## 14   15|16        3.022  0.002   NA       NA       NA 0.00e+00
```

```
## 15   16|17        3.790  0.002  NA       NA       NA 0.00e+00
## 16   17|18        4.589  0.002  NA       NA       NA 0.00e+00
## 17   18|20        5.296  0.717  NA       NA       NA 1.47e-13
## 18 drugMPH       -0.332  0.002 0.72     0.71     0.72 0.00e+00
## 19 drugREB       -0.133  0.002 0.88     0.87     0.88 0.00e+00
```

```r
data_perf$NASA_Effort <- factor(data_perf$NASA_Effort, levels = 1:20, ordered = TRUE)
model_NASA_Effort <- clmm(NASA_Effort ~ drug + (1 | record_id), data = data_perf) # the data behaves no
summary(model_NASA_Effort)
```

```
## Cumulative Link Mixed Model fitted with the Laplace approximation
##
## formula: NASA_Effort ~ drug + (1 | record_id)
## data:    data_perf
##
##  link  threshold nobs logLik AIC    niter     max.grad cond.H
##  logit flexible  54   -99.59 221.19 665(3156) 4.31e-06 1.5e+02
##
## Random effects:
##  Groups    Name        Variance Std.Dev.
##  record_id (Intercept) 3.46     1.86
## Number of groups:  record_id 18
##
## Coefficients:
##         Estimate Std. Error z value Pr(>|z|)
## drugMPH  0.02641    0.62552   0.042    0.966
## drugREB -0.53317    0.60108  -0.887    0.375
##
## Threshold coefficients:
##       Estimate Std. Error z value
## 12|13  -5.8198     1.3968  -4.167
## 13|14  -4.5641     1.0902  -4.186
## 14|15  -2.9457     0.8242  -3.574
## 15|16  -2.4603     0.7607  -3.234
## 16|17  -0.8935     0.6580  -1.358
## 17|18  -0.2951     0.6479  -0.455
## 18|19   0.7697     0.6546   1.176
## 19|20   2.9928     0.8059   3.714
```

```r
report_clmm_effects(model_NASA_Effort)
```

```
##       Term Estimate_log SE_log   OR CI_lower CI_upper  p_value
## 1    12|13       -5.820  1.397   NA       NA       NA 3.09e-05
## 2    13|14       -4.564  1.090   NA       NA       NA 2.83e-05
## 3    14|15       -2.946  0.824   NA       NA       NA 3.52e-04
## 4    15|16       -2.460  0.761   NA       NA       NA 1.22e-03
## 5    16|17       -0.894  0.658   NA       NA       NA 1.74e-01
## 6    17|18       -0.295  0.648   NA       NA       NA 6.49e-01
## 7    18|19        0.770  0.655   NA       NA       NA 2.40e-01
## 8    19|20        2.993  0.806   NA       NA       NA 2.04e-04
## 9  drugMPH        0.026  0.626 1.03     0.30     3.50 9.66e-01
## 10 drugREB       -0.533  0.601 0.59     0.18     1.91 3.75e-01
```

```r
data_perf$NASA_Performance <- factor(data_perf$NASA_Performance, levels = 1:20, ordered = TRUE)
model_NASA_Performance <- clmm(NASA_Performance ~ drug + (1 | record_id), data = data_perf) # the data
```

```
## Warning in update.uC(rho): Non finite negative log-likelihood
##   at iteration 171
```

```r
summary(model_NASA_Performance)
```

```
## Cumulative Link Mixed Model fitted with the Laplace approximation
##
## formula: NASA_Performance ~ drug + (1 | record_id)
## data:    data_perf
##
##  link  threshold nobs logLik  AIC    niter       max.grad cond.H
##  logit flexible  54   -129.85 299.70 2189(11009) 1.28e-04 1.3e+03
##
## Random effects:
##  Groups    Name        Variance Std.Dev.
##  record_id (Intercept) 7.463    2.732
## Number of groups:  record_id 18
##
## Coefficients:
##         Estimate Std. Error z value Pr(>|z|)
## drugMPH  -1.7470     0.6617  -2.640  0.00828 **
## drugREB  -0.2621     0.6120  -0.428  0.66848
## ---
## Signif. codes:  0 '***' 0.001 '**' 0.01 '*' 0.05 '.' 0.1 ' ' 1
##
## Threshold coefficients:
##       Estimate Std. Error z value
## 1|2    -4.1914     1.0685  -3.923
## 2|3    -2.9636     0.9399  -3.153
## 3|4    -2.5772     0.9167  -2.812
## 4|5    -1.4646     0.8623  -1.698
## 5|6    -0.8097     0.8458  -0.957
## 6|7    -0.2247     0.8427  -0.267
## 7|8     0.4988     0.8483   0.588
## 8|9     1.1302     0.8584   1.317
## 9|10    1.3013     0.8619   1.510
## 10|11   1.4730     0.8663   1.700
## 11|12   1.6456     0.8715   1.888
## 12|13   2.4268     0.9131   2.658
## 13|14   2.9448     0.9561   3.080
## 14|15   3.2808     0.9935   3.302
## 15|16   3.6771     1.0468   3.513
## 16|17   4.1819     1.1374   3.677
## 17|18   6.6741     1.8465   3.614
```

```r
report_clmm_effects(model_NASA_Performance)
```

```
##       Term Estimate_log SE_log   OR CI_lower CI_upper  p_value
## 1      1|2       -4.191  1.069   NA       NA       NA 8.76e-05
```

```
## 2       2|3       -2.964  0.940   NA       NA       NA 1.62e-03
## 3       3|4       -2.577  0.917   NA       NA       NA 4.93e-03
## 4       4|5       -1.465  0.862   NA       NA       NA 8.94e-02
## 5       5|6       -0.810  0.846   NA       NA       NA 3.38e-01
## 6       6|7       -0.225  0.843   NA       NA       NA 7.90e-01
## 7       7|8        0.499  0.848   NA       NA       NA 5.57e-01
## 8       8|9        1.130  0.858   NA       NA       NA 1.88e-01
## 9      9|10        1.301  0.862   NA       NA       NA 1.31e-01
## 10    10|11        1.473  0.866   NA       NA       NA 8.91e-02
## 11    11|12        1.646  0.872   NA       NA       NA 5.90e-02
## 12    12|13        2.427  0.913   NA       NA       NA 7.86e-03
## 13    13|14        2.945  0.956   NA       NA       NA 2.07e-03
## 14    14|15        3.281  0.993   NA       NA       NA 9.58e-04
## 15    15|16        3.677  1.047   NA       NA       NA 4.43e-04
## 16    16|17        4.182  1.137   NA       NA       NA 2.36e-04
## 17    17|18        6.674  1.847   NA       NA       NA 3.01e-04
## 18 drugMPH        -1.747  0.662 0.17     0.05     0.64 8.28e-03
## 19 drugREB        -0.262  0.612 0.77     0.23     2.55 6.68e-01
```

```
data_perf$NASA_Frustration <- factor(data_perf$NASA_Frustration, levels = 1:20, ordered = TRUE)
model_NASA_Frustration <- clmm(NASA_Frustration ~ drug + (1 | record_id), data = data_perf) # the data
summary(model_NASA_Frustration)
```

```
## Cumulative Link Mixed Model fitted with the Laplace approximation
##
## formula: NASA_Frustration ~ drug + (1 | record_id)
## data:    data_perf
##
##  link  threshold nobs logLik  AIC    niter      max.grad cond.H
##  logit flexible  47   -116.92 271.83 1991(7621) 5.84e-05 8.3e+02
##
## Random effects:
##  Groups    Name        Variance Std.Dev.
##  record_id (Intercept) 3.277    1.81
## Number of groups:  record_id 18
##
## Coefficients:
##         Estimate Std. Error z value Pr(>|z|)
## drugMPH  -1.3281     0.6953  -1.910   0.0561 .
## drugREB  -1.2770     0.6987  -1.828   0.0676 .
## ---
## Signif. codes:  0 '***' 0.001 '**' 0.01 '*' 0.05 '.' 0.1 ' ' 1
##
## Threshold coefficients:
##       Estimate Std. Error z value
## 1|2    -5.4040     1.2657  -4.270
## 2|3    -3.6002     0.9637  -3.736
## 3|4    -1.9807     0.7582  -2.612
## 4|5    -1.8046     0.7407  -2.436
## 5|6    -0.9994     0.6858  -1.457
## 6|7    -0.4302     0.6699  -0.642
## 7|8    -0.2914     0.6680  -0.436
## 8|9     0.1149     0.6657   0.173
## 9|10    0.2505     0.6658   0.376
```

```
## 10|11   0.6978     0.6693   1.043
## 11|12   1.0114     0.6775   1.493
## 12|13   1.3409     0.6917   1.939
## 13|14   2.4246     0.7822   3.100
## 14|15   2.7302     0.8233   3.316
## 15|17   3.0845     0.8787   3.510
## 17|18   4.2682     1.2029   3.548
## (7 observations deleted due to missingness)
```

```
report_clmm_effects(model_NASA_Frustration)
```

```
##        Term Estimate_log SE_log   OR CI_lower CI_upper  p_value
## 1       1|2       -5.404  1.266   NA       NA       NA 1.96e-05
## 2       2|3       -3.600  0.964   NA       NA       NA 1.87e-04
## 3       3|4       -1.981  0.758   NA       NA       NA 8.99e-03
## 4       4|5       -1.805  0.741   NA       NA       NA 1.48e-02
## 5       5|6       -0.999  0.686   NA       NA       NA 1.45e-01
## 6       6|7       -0.430  0.670   NA       NA       NA 5.21e-01
## 7       7|8       -0.291  0.668   NA       NA       NA 6.63e-01
## 8       8|9        0.115  0.666   NA       NA       NA 8.63e-01
## 9      9|10        0.251  0.666   NA       NA       NA 7.07e-01
## 10    10|11        0.698  0.669   NA       NA       NA 2.97e-01
## 11    11|12        1.011  0.677   NA       NA       NA 1.35e-01
## 12    12|13        1.341  0.692   NA       NA       NA 5.25e-02
## 13    13|14        2.425  0.782   NA       NA       NA 1.94e-03
## 14    14|15        2.730  0.823   NA       NA       NA 9.12e-04
## 15    15|17        3.084  0.879   NA       NA       NA 4.47e-04
## 16    17|18        4.268  1.203   NA       NA       NA 3.88e-04
## 17 drugMPH        -1.328  0.695 0.26     0.07     1.04 5.61e-02
## 18 drugREB        -1.277  0.699 0.28     0.07     1.10 6.76e-02
```

4.66 MODEL ASSUMPTION CHECK

```
#  the nominal_test and scale_test functions don't work with clmm model objects, so we will define a si

model_POA_mental <- clm(NASA_Mental ~ drug, data = data_perf)
po_test_mental <- nominal_test(model_POA_mental)
summary(po_test_mental)
```

```
##         Df           logLik            AIC              LRT           Pr(>Chi)
##  Min.   : NA   Min.   :-123.5   Min.   :280.9   Min.   : NA   Min.   : NA
##  1st Qu.: NA   1st Qu.:-123.5   1st Qu.:280.9   1st Qu.: NA   1st Qu.: NA
##  Median : NA   Median :-123.5   Median :280.9   Median : NA   Median : NA
##  Mean   :NaN   Mean   :-123.5   Mean   :280.9   Mean   :NaN   Mean   :NaN
##  3rd Qu.: NA   3rd Qu.:-123.5   3rd Qu.:280.9   3rd Qu.: NA   3rd Qu.: NA
##  Max.   : NA   Max.   :-123.5   Max.   :280.9   Max.   : NA   Max.   : NA
##  NA's   :2     NA's   :1        NA's   :1       NA's   :2     NA's   :2
```

```r
model_POA_physical <- clm(NASA_Physical ~ drug, data = data_perf)
po_test_physical <- nominal_test(model_POA_physical)
summary(po_test_physical)
```

```
##        Df          logLik             AIC              LRT             Pr(>Chi)
##  Min.   :10   Min.   :-96.87   Min.   :209.7   Min.   :6.897   Min.   :0.7351
##  1st Qu.:10   1st Qu.:-96.00   1st Qu.:213.0   1st Qu.:6.897   1st Qu.:0.7351
##  Median :10   Median :-95.14   Median :216.3   Median :6.897   Median :0.7351
##  Mean   :10   Mean   :-95.14   Mean   :216.3   Mean   :6.897   Mean   :0.7351
##  3rd Qu.:10   3rd Qu.:-94.28   3rd Qu.:219.6   3rd Qu.:6.897   3rd Qu.:0.7351
##  Max.   :10   Max.   :-93.42   Max.   :222.8   Max.   :6.897   Max.   :0.7351
##  NA's   :1                                     NA's   :1       NA's   :1
```

```r
model_POA_temporal <- clm(NASA_Temporal ~ drug, data = data_perf)
po_test_temporal <- nominal_test(model_POA_temporal)
summary(po_test_temporal)
```

```
##        Df           logLik             AIC              LRT           Pr(>Chi)
##  Min.   : NA   Min.   :-136.8   Min.   :311.6   Min.   : NA   Min.   : NA
##  1st Qu.: NA   1st Qu.:-136.8   1st Qu.:311.6   1st Qu.: NA   1st Qu.: NA
##  Median : NA   Median :-136.8   Median :311.6   Median : NA   Median : NA
##  Mean   :NaN   Mean   :-136.8   Mean   :311.6   Mean   :NaN   Mean   :NaN
##  3rd Qu.: NA   3rd Qu.:-136.8   3rd Qu.:311.6   3rd Qu.: NA   3rd Qu.: NA
##  Max.   : NA   Max.   :-136.8   Max.   :311.6   Max.   : NA   Max.   : NA
##  NA's   :2     NA's   :1        NA's   :1       NA's   :2     NA's   :2
```

```r
model_POA_effort <- clm(NASA_Effort ~ drug, data = data_perf)
po_test_effort <- nominal_test(model_POA_effort)
summary(po_test_effort)
```

```
##        Df           logLik             AIC              LRT           Pr(>Chi)
##  Min.   : NA   Min.   :-105.7   Min.   :231.4   Min.   : NA   Min.   : NA
##  1st Qu.: NA   1st Qu.:-105.7   1st Qu.:231.4   1st Qu.: NA   1st Qu.: NA
##  Median : NA   Median :-105.7   Median :231.4   Median : NA   Median : NA
##  Mean   :NaN   Mean   :-105.7   Mean   :231.4   Mean   :NaN   Mean   :NaN
##  3rd Qu.: NA   3rd Qu.:-105.7   3rd Qu.:231.4   3rd Qu.: NA   3rd Qu.: NA
##  Max.   : NA   Max.   :-105.7   Max.   :231.4   Max.   : NA   Max.   : NA
##  NA's   :2     NA's   :1        NA's   :1       NA's   :2     NA's   :2
```

```r
model_POA_performance <- clm(NASA_Performance ~ drug, data = data_perf)
po_test_performance <- nominal_test(model_POA_performance)
summary(po_test_performance)
```

```
##        Df           logLik             AIC              LRT           Pr(>Chi)
##  Min.   : NA   Min.   :-141.8   Min.   :321.6   Min.   : NA   Min.   : NA
##  1st Qu.: NA   1st Qu.:-141.8   1st Qu.:321.6   1st Qu.: NA   1st Qu.: NA
##  Median : NA   Median :-141.8   Median :321.6   Median : NA   Median : NA
##  Mean   :NaN   Mean   :-141.8   Mean   :321.6   Mean   :NaN   Mean   :NaN
##  3rd Qu.: NA   3rd Qu.:-141.8   3rd Qu.:321.6   3rd Qu.: NA   3rd Qu.: NA
##  Max.   : NA   Max.   :-141.8   Max.   :321.6   Max.   : NA   Max.   : NA
##  NA's   :2     NA's   :1        NA's   :1       NA's   :2     NA's   :2
```

```r
model_POA_frustration <- clm(NASA_Frustration ~ drug, data = data_perf)
po_test_frustration <- nominal_test(model_POA_frustration)
summary(po_test_frustration)
```

```
##        Df          logLik            AIC             LRT          Pr(>Chi)
##  Min.   : NA   Min.   :-121.2   Min.   :278.3   Min.   : NA   Min.   : NA
##  1st Qu.: NA   1st Qu.:-121.2   1st Qu.:278.3   1st Qu.: NA   1st Qu.: NA
##  Median : NA   Median :-121.2   Median :278.3   Median : NA   Median : NA
##  Mean   :NaN   Mean   :-121.2   Mean   :278.3   Mean   :NaN   Mean   :NaN
##  3rd Qu.: NA   3rd Qu.:-121.2   3rd Qu.:278.3   3rd Qu.: NA   3rd Qu.: NA
##  Max.   : NA   Max.   :-121.2   Max.   :278.3   Max.   : NA   Max.   : NA
##  NA's   :2     NA's   :1        NA's   :1       NA's   :2     NA's   :2
```

4.7 PHYSIOLOGY - HR AND LACTATE

4.71 DESCRIPTIVES (HR AND LACTATE)

```r
# --------------------------------------HR/LACTATE -----------------------------------
# one delta score of pre- and post-fatigue
mean_data_phys <- data_phys_sub %>%
  group_by(drug, type) %>%
  summarise(
    mean_lactate = round(mean(lactate, na.rm = TRUE), 2),
    sd_lactate = round(sd(lactate, na.rm = TRUE), 2),
    mean_hr = round(mean(hr, na.rm = TRUE), 2),
    sd_hr = round(sd(hr, na.rm = TRUE), 2)
  )
```

```
## `summarise()` has grouped output by 'drug'. You can override using the
## `.groups` argument.
```

```r
# View the result
view(mean_data_phys)
```

4.72 DATA ANALYSES (HR AND LACTATE)

```r
# -----------------------------------ANALYSES MODELS
model_hr <- lmer(hr ~ drug + type + (1 | record_id), data = data_phys_sub)
model_hr_log <- lmer(log(hr) ~ drug * type + (1 | record_id), data = data_phys_sub)
summary(model_hr_log)
```

```
## Linear mixed model fit by REML. t-tests use Satterthwaite's method [
## lmerModLmerTest]
## Formula: log(hr) ~ drug * type + (1 | record_id)
##    Data: data_phys_sub
##
## REML criterion at convergence: -131.9
```

```
##
## Scaled residuals:
##      Min       1Q   Median       3Q      Max
## -2.14291 -0.65128  0.00681  0.59729  2.04217
##
## Random effects:
##  Groups    Name        Variance Std.Dev.
##  record_id (Intercept) 0.013149 0.11467
##  Residual              0.009318 0.09653
## Number of obs: 108, groups:  record_id, 18
##
## Fixed effects:
##                   Estimate Std. Error       df t value Pr(>|t|)
## (Intercept)        4.50882    0.03533 37.60191 127.623  < 2e-16 ***
## drugMPH            0.06553    0.03218 85.00000   2.037   0.0448 *
## drugREB            0.14365    0.03218 85.00000   4.465 2.45e-05 ***
## typepost           0.43998    0.03218 85.00000  13.674  < 2e-16 ***
## drugMPH:typepost   0.03439    0.04550 85.00000   0.756   0.4519
## drugREB:typepost  -0.05265    0.04550 85.00000  -1.157   0.2505
## ---
## Signif. codes:  0 '***' 0.001 '**' 0.01 '*' 0.05 '.' 0.1 ' ' 1
##
## Correlation of Fixed Effects:
##             (Intr) drgMPH drgREB typpst drMPH:
## drugMPH     -0.455
## drugREB     -0.455  0.500
## typepost    -0.455  0.500  0.500
## drgMPH:typp  0.322 -0.707 -0.354 -0.707
## drgREB:typp  0.322 -0.354 -0.707 -0.707  0.500
```

```
summarize_lmer_effects(model_hr_log, inverse = exp)
```

```
##               Term Estimate   SE Estimate_inv CI_lower CI_upper p_value
## 1      (Intercept)     4.51 0.04        90.81    84.74    97.33   0.000
## 2          drugMPH     0.07 0.03         1.07     1.00     1.14   0.045
## 3          drugREB     0.14 0.03         1.15     1.08     1.23   0.000
## 4         typepost     0.44 0.03         1.55     1.46     1.65   0.000
## 5 drugMPH:typepost     0.03 0.05         1.03     0.95     1.13   0.452
## 6 drugREB:typepost    -0.05 0.05         0.95     0.87     1.04   0.251
```

```
anova(model_hr,model_hr_log)
```

```
## refitting model(s) with ML (instead of REML)
```

```
## Data: data_phys_sub
## Models:
## model_hr: hr ~ drug + type + (1 | record_id)
## model_hr_log: log(hr) ~ drug * type + (1 | record_id)
##              npar     AIC     BIC logLik deviance  Chisq Df Pr(>Chisq)
## model_hr        6  892.00  908.10 -440.0   880.00
## model_hr_log    8 -148.21 -126.75   82.1  -164.21 1044.2  2  < 2.2e-16 ***
## ---
## Signif. codes:  0 '***' 0.001 '**' 0.01 '*' 0.05 '.' 0.1 ' ' 1
```

```r
sim_res2 <- simulateResiduals(model_hr) #
# Plot diagnostics
plot(sim_res2) #OK
```

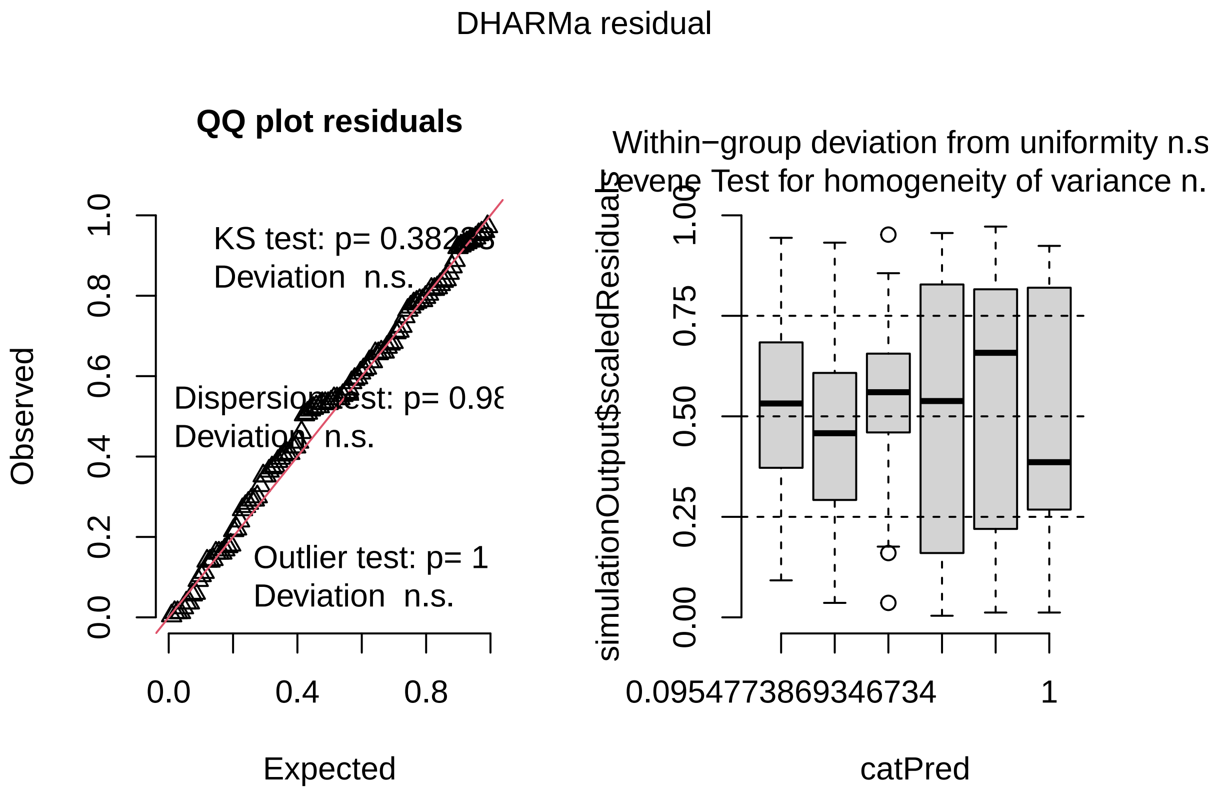


```r
sim_res2 <- simulateResiduals(model_hr_log) #
# Plot diagnostics
plot(sim_res2) #OK
```

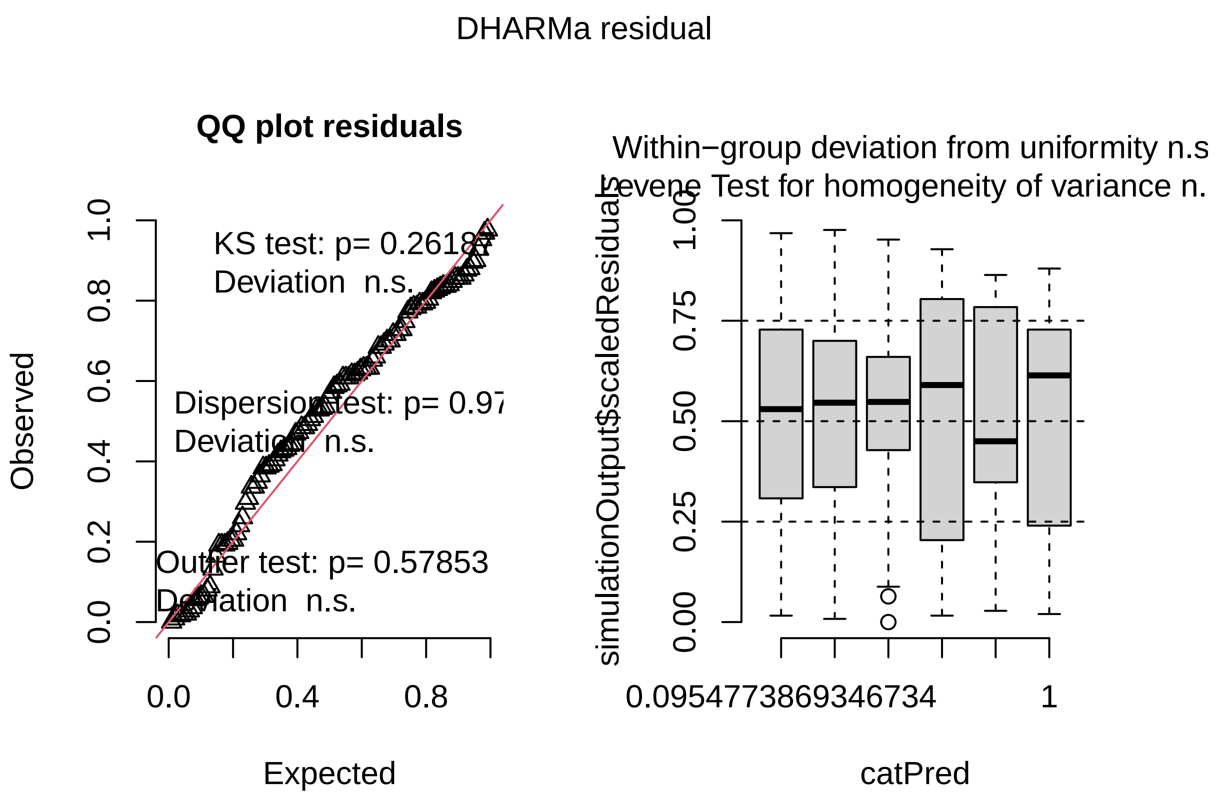


```r
model_lactate <- lmer(lactate ~ drug + type + (1 | record_id), data = data_phys_sub)
summary(model_lactate)
```

```
## Linear mixed model fit by REML. t-tests use Satterthwaite's method [
## lmerModLmerTest]
## Formula: lactate ~ drug + type + (1 | record_id)
##    Data: data_phys_sub
##
## REML criterion at convergence: 418.5
##
## Scaled residuals:
##      Min       1Q   Median       3Q      Max
## -2.24102 -0.65872  0.01624  0.63106  2.87096
##
## Random effects:
##  Groups    Name        Variance Std.Dev.
##  record_id (Intercept) 1.459    1.208
##  Residual              2.200    1.483
## Number of obs: 108, groups:  record_id, 18
##
## Fixed effects:
##             Estimate Std. Error      df t value Pr(>|t|)
## (Intercept)   2.6431     0.4032 40.7669   6.555 7.20e-08 ***
## drugMPH       0.1797     0.3496 87.0000   0.514   0.6085
## drugREB       0.8633     0.3496 87.0000   2.469   0.0155 *
```

```
## typepost      2.4639     0.2855 87.0000   8.631 2.56e-13 ***
## ---
## Signif. codes:  0 '***' 0.001 '**' 0.01 '*' 0.05 '.' 0.1 ' ' 1
##
## Correlation of Fixed Effects:
##          (Intr) drgMPH drgREB
## drugMPH  -0.434
## drugREB  -0.434  0.500
## typepost -0.354  0.000  0.000
```

```
model_lactate_log <- lmer(log(lactate) ~ drug * type + (1 | record_id), data = data_phys_sub)
summary(model_lactate_log) # this one
```

```
## Linear mixed model fit by REML. t-tests use Satterthwaite's method [
## lmerModLmerTest]
## Formula: log(lactate) ~ drug * type + (1 | record_id)
##    Data: data_phys_sub
##
## REML criterion at convergence: 119.1
##
## Scaled residuals:
##      Min       1Q   Median       3Q      Max
## -2.56871 -0.57561  0.09114  0.72666  1.97831
##
## Random effects:
##  Groups    Name        Variance Std.Dev.
##  record_id (Intercept) 0.06112  0.2472
##  Residual              0.12655  0.3557
## Number of obs: 108, groups:  record_id, 18
##
## Fixed effects:
##                   Estimate Std. Error       df t value Pr(>|t|)
## (Intercept)        0.96904    0.10211 66.65580   9.490 5.37e-14 ***
## drugMPH            0.04520    0.11858 85.00000   0.381    0.704
## drugREB            0.07931    0.11858 85.00000   0.669    0.505
## typepost           0.53762    0.11858 85.00000   4.534 1.88e-05 ***
## drugMPH:typepost   0.01106    0.16770 85.00000   0.066    0.948
## drugREB:typepost   0.14570    0.16770 85.00000   0.869    0.387
## ---
## Signif. codes:  0 '***' 0.001 '**' 0.01 '*' 0.05 '.' 0.1 ' ' 1
##
## Correlation of Fixed Effects:
##             (Intr) drgMPH drgREB typpst drMPH:
## drugMPH     -0.581
## drugREB     -0.581  0.500
## typepost    -0.581  0.500  0.500
## drgMPH:typp  0.411 -0.707 -0.354 -0.707
## drgREB:typp  0.411 -0.354 -0.707 -0.707  0.500
```

```
summarize_lmer_effects(model_lactate_log, inverse = exp)
```

```
##                Term Estimate   SE Estimate_inv CI_lower CI_upper p_value
## 1       (Intercept)     0.97 0.10         2.64     2.16     3.22   0.000
```

```
## 2          drugMPH     0.05 0.12          1.05      0.83      1.32    0.704
## 3          drugREB     0.08 0.12          1.08      0.86      1.37    0.505
## 4         typepost     0.54 0.12          1.71      1.36      2.16    0.000
## 5 drugMPH:typepost     0.01 0.17          1.01      0.73      1.40    0.948
## 6 drugREB:typepost     0.15 0.17          1.16      0.83      1.61    0.387
```

```r
sim_res2 <- simulateResiduals(model_lactate_log) #
# Plot diagnostics
plot(sim_res2) #OK
```

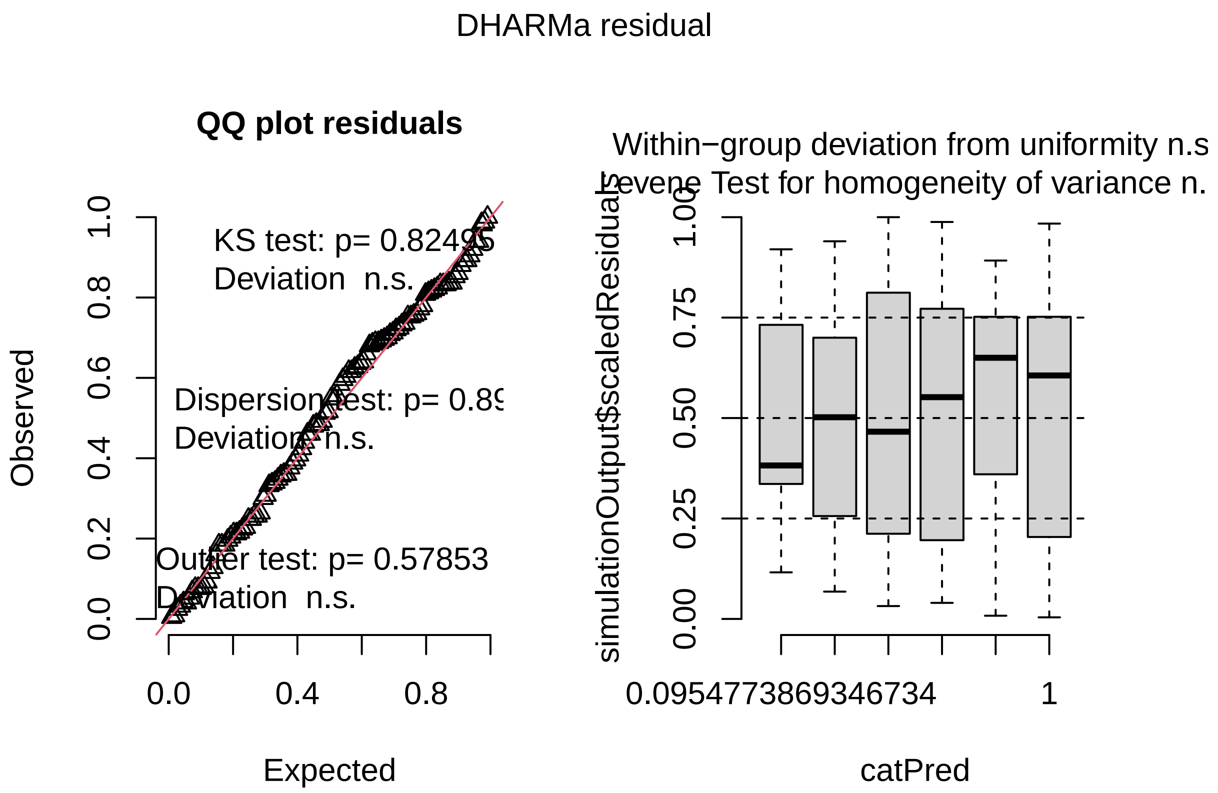

4.8 TMG - PHYSIOLOGY

4.81 DESCRIPTIVES and visualization FOR TC AND DM

```r
# --------------------------------------TMG --------------------
#  datapoints ( pre, post) with three conditions. Really a check!!!

# Now compute group summaries on the filtered data
mean_data_tmg <- data_tmg_sub %>%
  group_by(drug, time) %>%
  summarise(
    mean_tc = round(mean(tc, na.rm = TRUE), 2),
    sd_tc   = round(sd(tc, na.rm = TRUE), 2),
```

```r
    mean_dm = round(mean(dm, na.rm = TRUE), 2),
    sd_dm   = round(sd(dm, na.rm = TRUE), 2),
    .groups = "drop"  # optional: ungroups result after summarise
  )

# View the summary table
view(mean_data_tmg)
```

```r
###---------------------------- Tc
# Boxplot for Contraction Time (tc)
tc_boxplot <-
  ggplot(data_tmg_sub, aes(x = factor(time), y = tc, fill = drug)) +
  geom_boxplot(alpha = 0.3, outlier.size = 2) +  # Boxplot with transparency
  geom_jitter(aes(color = drug),
              position = position_jitterdodge(jitter.width = 0.2, dodge.width = 0.8),
              size = 1.5, alpha = 0.6) +  # Show raw points
  stat_summary(fun = mean, geom = "point",
               position = position_dodge(width = 0.8),
               size = 3, shape = 18, aes(color = drug)) +
  labs(title = "Contraction Time by Time and Drug",
       x = "Time", y = "Contraction Time (ms)") +
  theme_minimal() +
  scale_fill_brewer(palette = "Set2") +  # Color palette for fill
  scale_color_brewer(palette = "Set2")   # Color palette for points

tc_boxplot
```

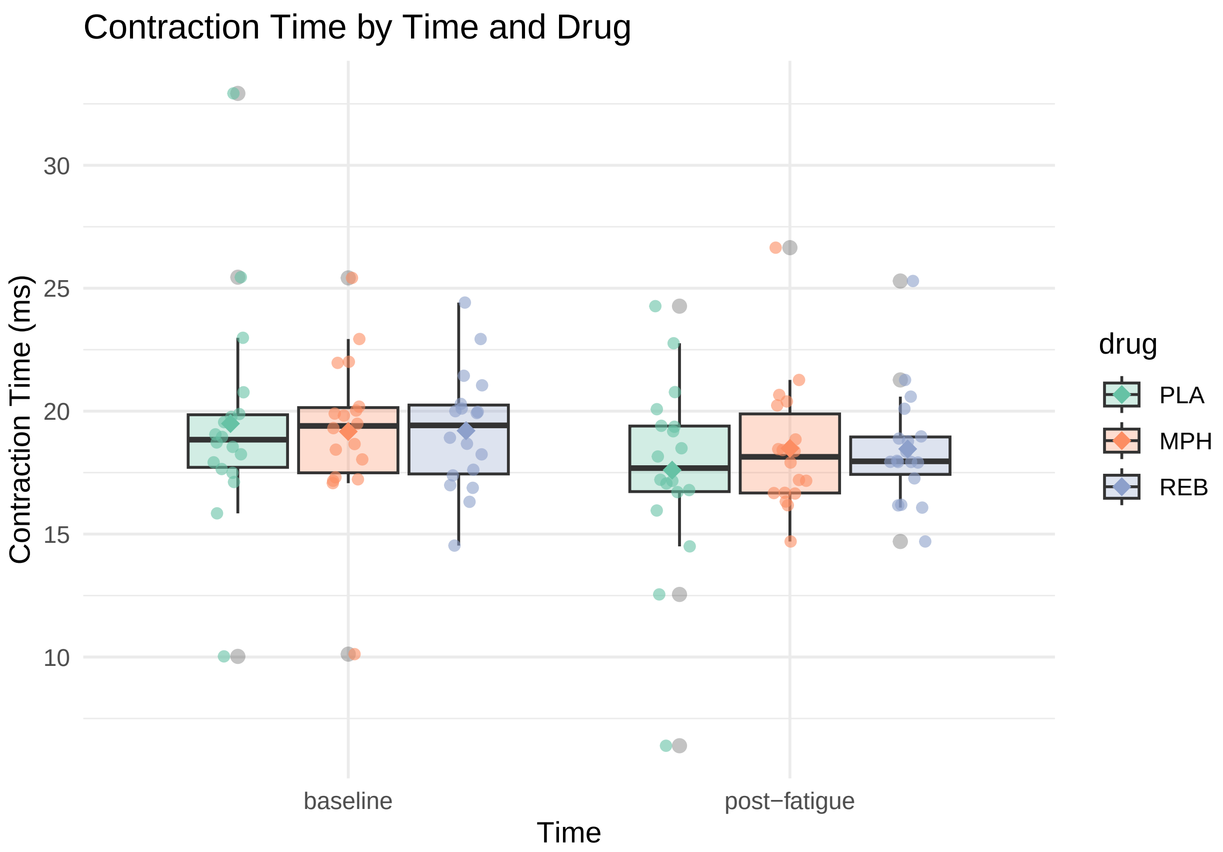


```r
# Histogram for Contraction Time (tc)
tc_hist <-
  ggplot(data_tmg_sub, aes(x = tc, fill = drug)) +
  geom_histogram(binwidth = 1, position = "dodge", color = "black", alpha = 0.7) +
  facet_wrap(~ time) +
  labs(title = "Contraction Time Distribution by Time and Drug",
       x = "Contraction Time (ms)", y = "Count") +
  theme_minimal() +
  scale_fill_brewer(palette = "Set2")

tc_hist
```

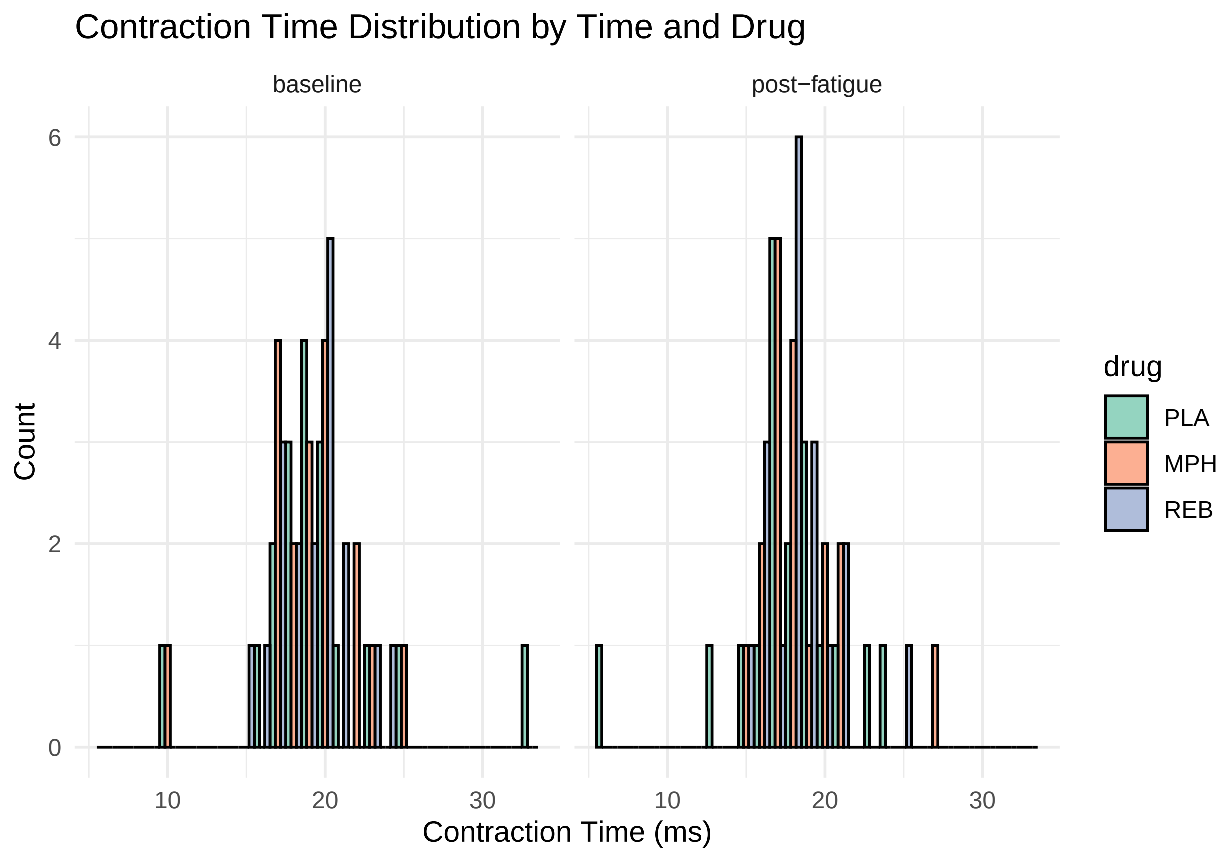


```r
# Combine boxplot and histogram
tc_boxplot / tc_hist + plot_layout(heights = c(2, 1))
```

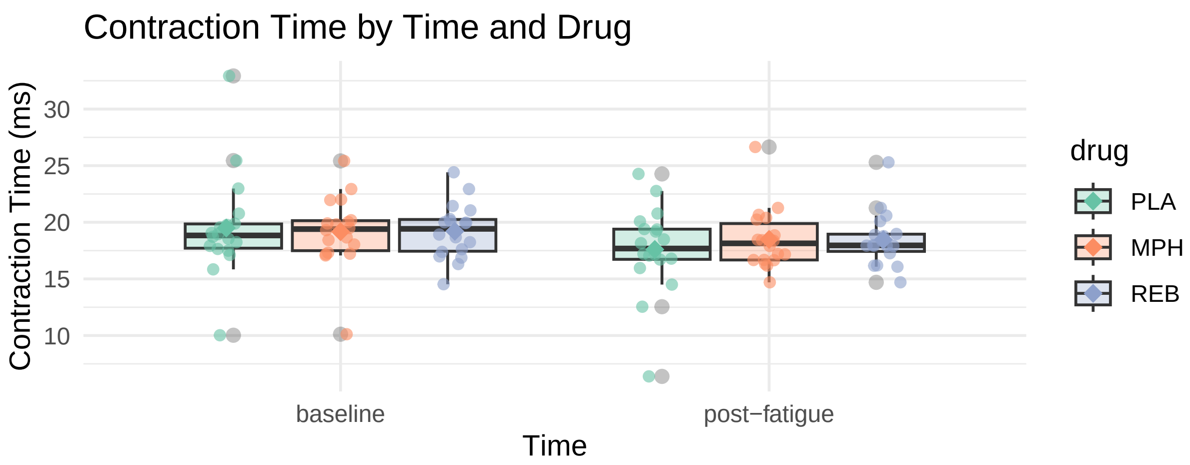

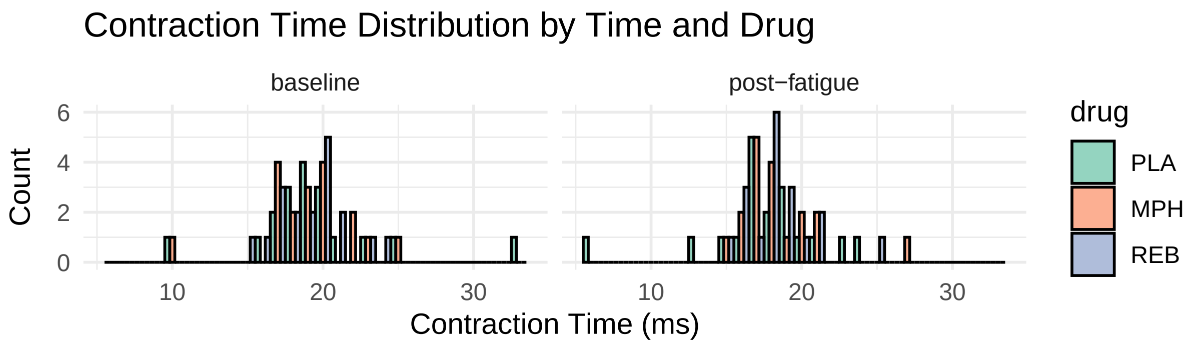

4.82 DATA ANALYSIS FOR TC

```r
# Fit the linear mixed-effects model on the subset
model_tmgtc <- lmer(tc ~ drug * time + (1 | record_id), data = data_tmg_sub)

# Show model summary
summary(model_tmgtc)
```

```
## Linear mixed model fit by REML. t-tests use Satterthwaite's method [
## lmerModLmerTest]
## Formula: tc ~ drug * time + (1 | record_id)
##    Data: data_tmg_sub
##
## REML criterion at convergence: 501.7
##
## Scaled residuals:
##     Min      1Q  Median      3Q     Max
## -2.6628 -0.4976 -0.0374  0.3763  4.2779
##
## Random effects:
##  Groups    Name        Variance Std.Dev.
##  record_id (Intercept) 6.148    2.480
##  Residual              4.701    2.168
## Number of obs: 108, groups:  record_id, 18
```

```
##
## Fixed effects:
##                          Estimate Std. Error      df t value Pr(>|t|)
## (Intercept)               19.4945     0.7763 39.1443  25.111   <2e-16 ***
## drugMPH                   -0.3231     0.7227 85.0000  -0.447   0.6559
## drugREB                   -0.2894     0.7227 85.0000  -0.400   0.6898
## timepost-fatigue          -1.8937     0.7227 85.0000  -2.620   0.0104 *
## drugMPH:timepost-fatigue   1.2101     1.0221 85.0000   1.184   0.2397
## drugREB:timepost-fatigue   1.1515     1.0221 85.0000   1.127   0.2631
## ---
## Signif. codes:  0 '***' 0.001 '**' 0.01 '*' 0.05 '.' 0.1 ' ' 1
##
## Correlation of Fixed Effects:
##              (Intr) drgMPH drgREB tmpst- dMPH:-
## drugMPH      -0.465
## drugREB      -0.465  0.500
## timepst-ftg -0.465  0.500  0.500
## drgMPH:tmp-  0.329 -0.707 -0.354 -0.707
## drgREB:tmp-  0.329 -0.354 -0.707 -0.707  0.500
```

```r
summarize_lmer_effects(model_tmgtc)
```

```
##                       Term Estimate   SE Estimate_inv CI_lower CI_upper p_value
## 1              (Intercept)    19.49 0.78        19.49    17.97    21.02   0.000
## 2                  drugMPH    -0.32 0.72        -0.32    -1.74     1.09   0.656
## 3                  drugREB    -0.29 0.72        -0.29    -1.71     1.13   0.690
## 4         timepost-fatigue    -1.89 0.72        -1.89    -3.31    -0.48   0.010
## 5 drugMPH:timepost-fatigue     1.21 1.02         1.21    -0.79     3.21   0.240
## 6 drugREB:timepost-fatigue     1.15 1.02         1.15    -0.85     3.15   0.263
```

```r
# Fit the linear mixed-effects model on the subset with sqrt
data_tmg_sub$tc_sqrt <- sqrt(data_tmg_sub$tc)
model_tmgtc_sqrt <- lmer(tc_sqrt ~ drug * time + (1 | record_id), data = data_tmg_sub)
summary(model_tmgtc_sqrt)
```

```
## Linear mixed model fit by REML. t-tests use Satterthwaite's method [
## lmerModLmerTest]
## Formula: tc_sqrt ~ drug * time + (1 | record_id)
##    Data: data_tmg_sub
##
## REML criterion at convergence: 66.6
##
## Scaled residuals:
##     Min      1Q  Median      3Q     Max
## -3.5702 -0.4820 -0.0255  0.3676  3.4928
##
## Random effects:
##  Groups    Name        Variance Std.Dev.
##  record_id (Intercept) 0.08607  0.2934
##  Residual              0.06604  0.2570
## Number of obs: 108, groups:  record_id, 18
##
## Fixed effects:
```

```
##                            Estimate Std. Error       df t value Pr(>|t|)    
## (Intercept)                 4.38809    0.09193 39.21704  47.735   <2e-16 ***
## drugMPH                    -0.02554    0.08566 85.00000  -0.298   0.7664    
## drugREB                    -0.01404    0.08566 85.00000  -0.164   0.8702    
## timepost-fatigue           -0.22349    0.08566 85.00000  -2.609   0.0107 *  
## drugMPH:timepost-fatigue    0.15069    0.12114 85.00000   1.244   0.2169    
## drugREB:timepost-fatigue    0.13838    0.12114 85.00000   1.142   0.2565    
## ---
## Signif. codes:  0 '***' 0.001 '**' 0.01 '*' 0.05 '.' 0.1 ' ' 1
##
## Correlation of Fixed Effects:
##             (Intr) drgMPH drgREB tmpst- dMPH:-
## drugMPH     -0.466                            
## drugREB     -0.466  0.500                     
## timepst-ftg -0.466  0.500  0.500              
## drgMPH:tmp-  0.329 -0.707 -0.354 -0.707       
## drgREB:tmp-  0.329 -0.354 -0.707 -0.707  0.500
```

```
summarize_lmer_effects(model_tmgtc_sqrt, inverse = function(x) x^2)
```

```
##                       Term Estimate   SE Estimate_inv CI_lower CI_upper p_value
## 1              (Intercept)     4.39 0.09        19.26    17.71    20.87   0.000
## 2                  drugMPH    -0.03 0.09         0.00     0.04     0.02   0.766
## 3                  drugREB    -0.01 0.09         0.00     0.03     0.02   0.870
## 4         timepost-fatigue    -0.22 0.09         0.05     0.15     0.00   0.011
## 5 drugMPH:timepost-fatigue     0.15 0.12         0.02     0.01     0.15   0.217
## 6 drugREB:timepost-fatigue     0.14 0.12         0.02     0.01     0.14   0.257
```

```
#--------------------------------------------------------ASSUMPTION CHECK
sim_res2 <- simulateResiduals(model_tmgtc) #
# Plot diagnostics
plot(sim_res2) # mweh can be better!
```

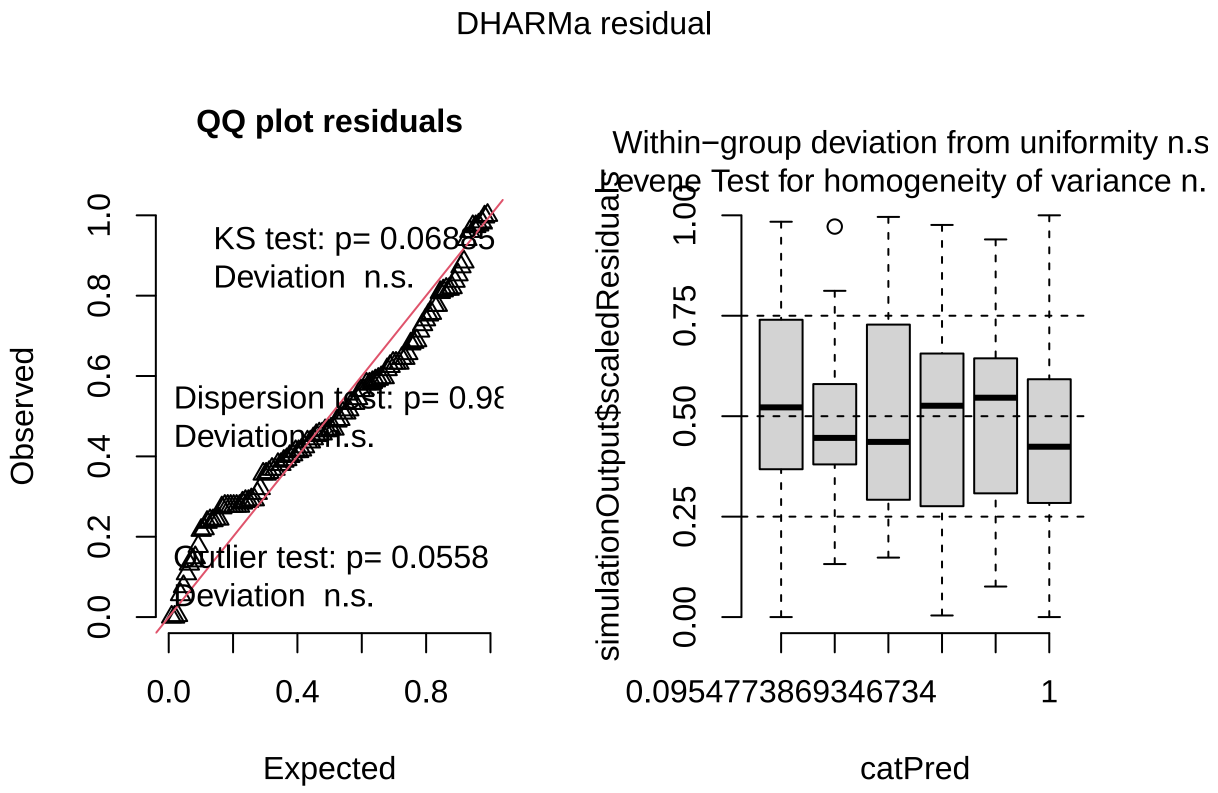


```
sim_res2 <- simulateResiduals(model_tmgtc_sqrt) #
# Plot diagnostics
plot(sim_res2) # NO deviation is significant so take other model? first with sqrt
```

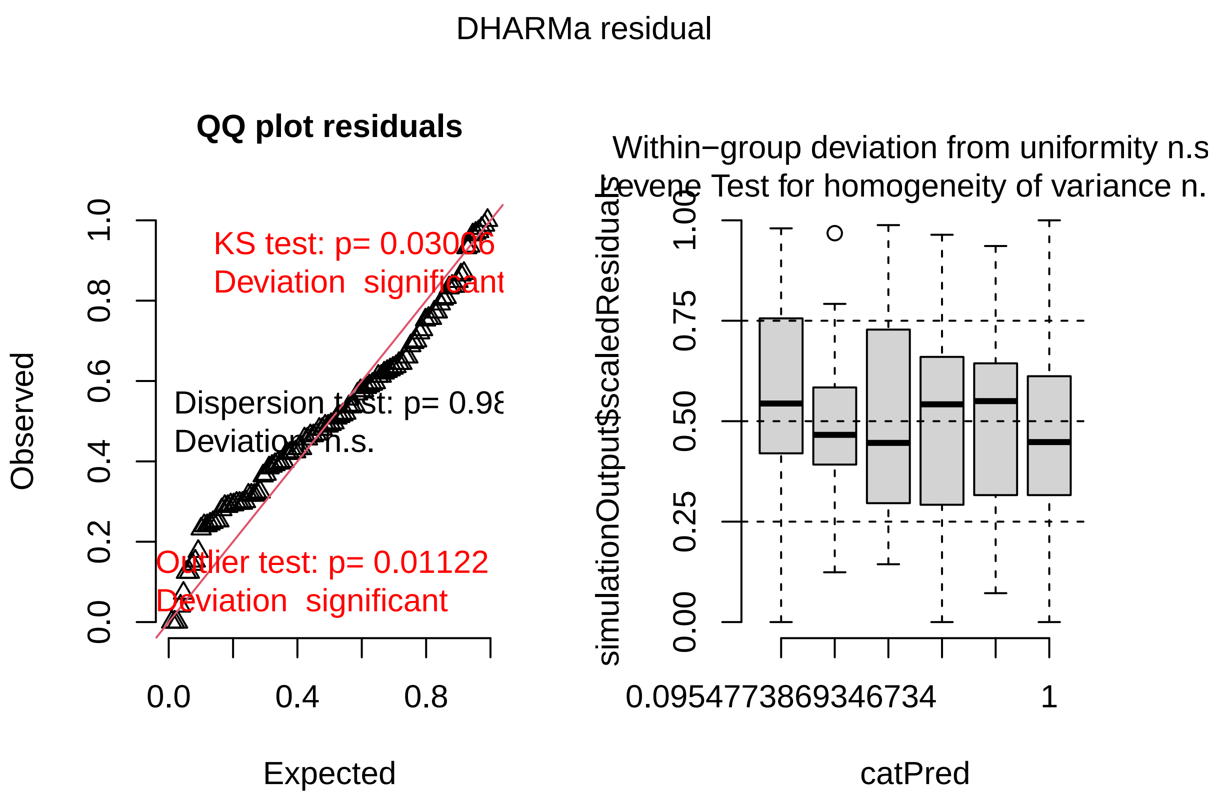


```
anova(model_tmgtc, model_tmgtc_sqrt) # sqrt is better but dharma is not correct so we go with lmm
```

```
## refitting model(s) with ML (instead of REML)

## Data: data_tmg_sub
## Models:
## model_tmgtc: tc ~ drug * time + (1 | record_id)
## model_tmgtc_sqrt: tc_sqrt ~ drug * time + (1 | record_id)
##                  npar    AIC    BIC   logLik deviance  Chisq Df Pr(>Chisq)
## model_tmgtc         8 522.72 544.17 -253.358   506.72
## model_tmgtc_sqrt    8  62.01  83.47  -23.005    46.01 460.71  0
```

4.83 DATA VISUALTIZATION FOR DM

```
###---------------------------- Dm
# Boxplot for DM Measure (dm_measure)
dm_boxplot <-
 ggplot(data_tmg_sub, aes(x = factor(time), y = dm, fill = drug)) +
  geom_boxplot(alpha = 0.3, outlier.size = 2) +  # Boxplot with transparency
  geom_jitter(aes(color = drug),
              position = position_jitterdodge(jitter.width = 0.2, dodge.width = 0.8),
              size = 1.5, alpha = 0.6) +  # Show raw points
  stat_summary(fun = mean, geom = "point",
```

```
                position = position_dodge(width = 0.8),
                size = 3, shape = 18, aes(color = drug)) +
  labs(title = "DM Measure by Time and Drug",
       x = "Time", y = "Dm") +
  theme_minimal() +
  scale_fill_brewer(palette = "Set2") +  # Color palette for fill
  scale_color_brewer(palette = "Set2")   # Color palette for points

dm_boxplot
```

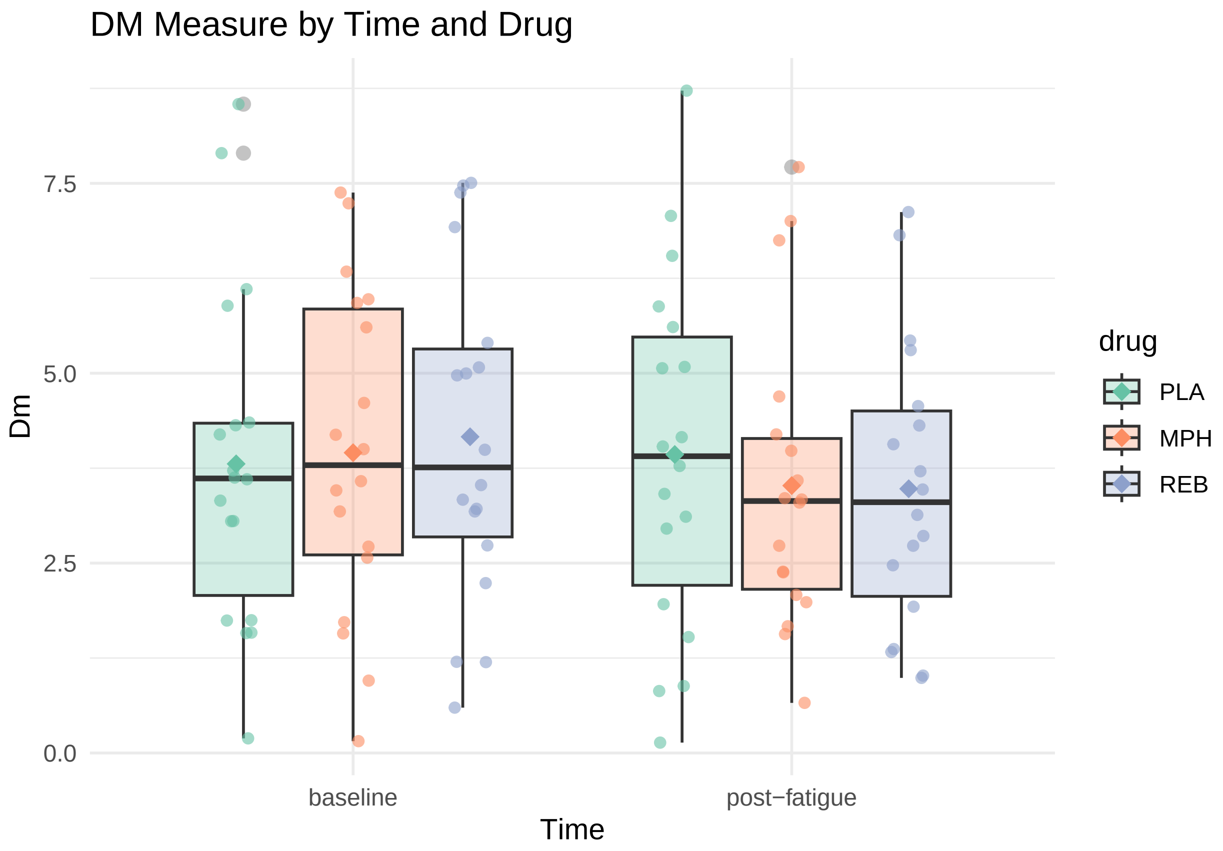


```
# Histogram for DM Measure (dm_measure)
dm_hist <-
 ggplot(data_tmg, aes(x = dm, fill = drug)) +
  geom_histogram(binwidth = 1, position = "dodge", color = "black", alpha = 0.7) +
  facet_wrap(~ time) +
  labs(title = "DM Measure Distribution by Time and Drug",
       x = "DM Measure", y = "Count") +
  theme_minimal() +
  scale_fill_brewer(palette = "Set2")

dm_hist
```

```
## Warning: Removed 1 row containing non-finite outside the scale range
## (‘stat_bin()‘).
```

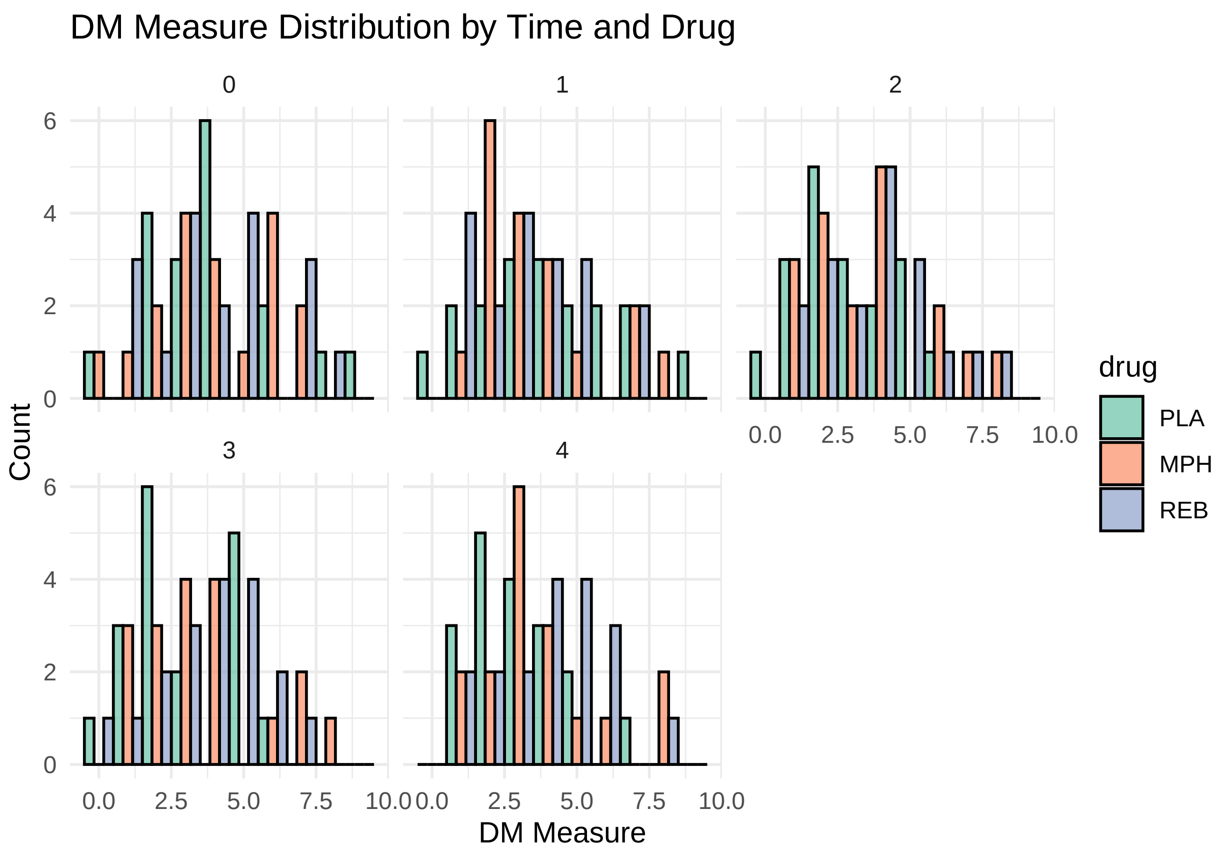


```r
# Combine boxplot and histogram
dm_boxplot / dm_hist + plot_layout(heights = c(2, 1))
```

```
## Warning: Removed 1 row containing non-finite outside the scale range
## (‘stat_bin()‘).
```

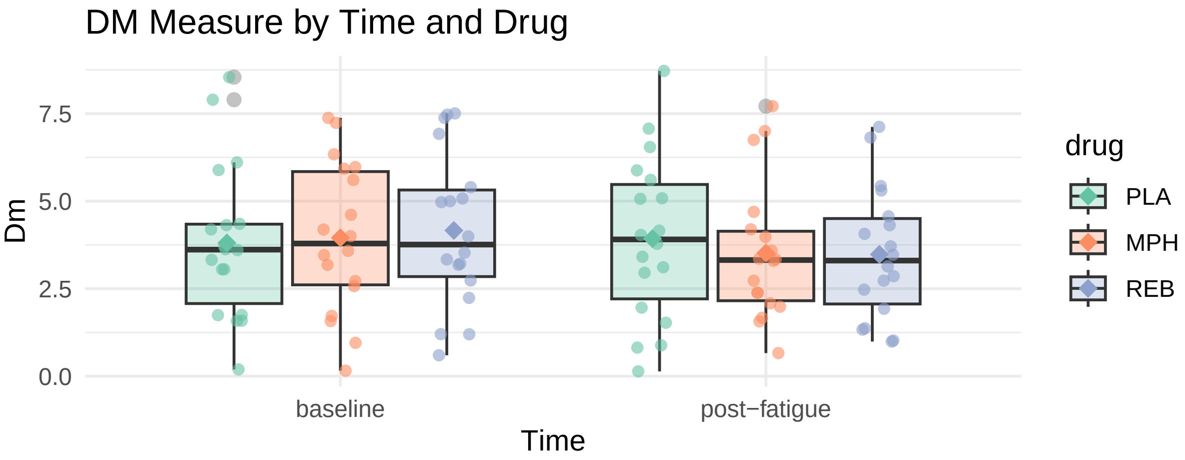

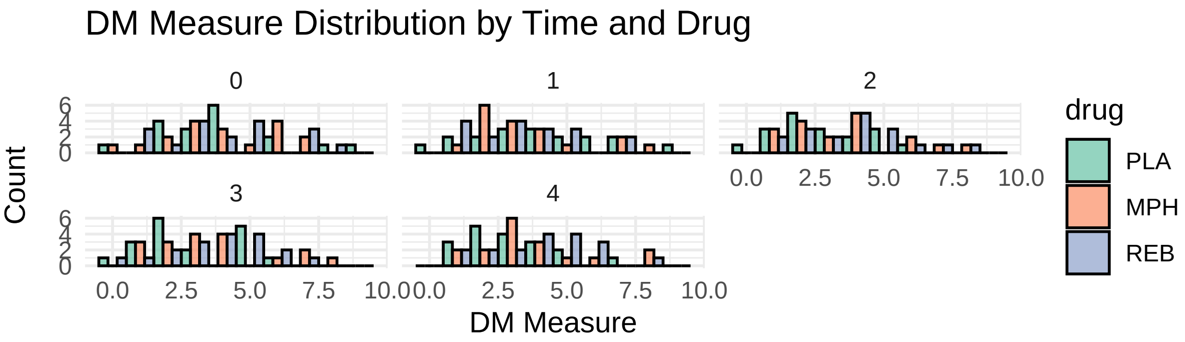

4.83 DATA ANALYSES FOR DM

```r
# Fit the linear mixed-effects model on the subset
model_tmgdm <- lmer(dm ~ drug * time + (1 | record_id), data = data_tmg_sub)
# Show model summary
summary(model_tmgdm)
```

```
## Linear mixed model fit by REML. t-tests use Satterthwaite's method [
## lmerModLmerTest]
## Formula: dm ~ drug * time + (1 | record_id)
##    Data: data_tmg_sub
##
## REML criterion at convergence: 400.4
##
## Scaled residuals:
##      Min       1Q   Median       3Q      Max
## -2.33165 -0.69315 -0.09293  0.56889  1.97301
##
## Random effects:
##  Groups    Name        Variance Std.Dev.
##  record_id (Intercept) 2.856    1.690
##  Residual              1.673    1.294
## Number of obs: 108, groups:  record_id, 18
##
```

```
## Fixed effects:
##                            Estimate Std. Error      df t value Pr(>|t|)
## (Intercept)                  3.8066     0.5016 34.1324   7.588 7.95e-09 ***
## drugMPH                      0.1469     0.4312 85.0000   0.341    0.734
## drugREB                      0.3566     0.4312 85.0000   0.827    0.410
## timepost-fatigue             0.1235     0.4312 85.0000   0.286    0.775
## drugMPH:timepost-fatigue  -0.5567     0.6098 85.0000  -0.913    0.364
## drugREB:timepost-fatigue  -0.8078     0.6098 85.0000  -1.325    0.189
## ---
## Signif. codes:  0 '***' 0.001 '**' 0.01 '*' 0.05 '.' 0.1 ' ' 1
##
## Correlation of Fixed Effects:
##             (Intr) drgMPH drgREB tmpst- dMPH:-
## drugMPH     -0.430
## drugREB     -0.430  0.500
## timepst-ftg -0.430  0.500  0.500
## drgMPH:tmp-  0.304 -0.707 -0.354 -0.707
## drgREB:tmp-  0.304 -0.354 -0.707 -0.707  0.500
```

```
summarize_lmer_effects(model_tmgdm)
```

```
##                       Term Estimate   SE Estimate_inv CI_lower CI_upper p_value
## 1              (Intercept)     3.81 0.50         3.81     2.82     4.79   0.000
## 2                  drugMPH     0.15 0.43         0.15    -0.70     0.99   0.734
## 3                  drugREB     0.36 0.43         0.36    -0.49     1.20   0.410
## 4         timepost-fatigue     0.12 0.43         0.12    -0.72     0.97   0.775
## 5 drugMPH:timepost-fatigue    -0.56 0.61        -0.56    -1.75     0.64   0.364
## 6 drugREB:timepost-fatigue    -0.81 0.61        -0.81    -2.00     0.39   0.189
```

```
data_tmg_sub$dm_sqrt <- sqrt(data_tmg_sub$dm)
model_tmgdm_sqrt <- lmer(dm_sqrt ~ drug * time + (1 | record_id),
                         data = data_tmg_sub)
summary(model_tmgdm_sqrt)
```

```
## Linear mixed model fit by REML. t-tests use Satterthwaite's method [
## lmerModLmerTest]
## Formula: dm_sqrt ~ drug * time + (1 | record_id)
##    Data: data_tmg_sub
##
## REML criterion at convergence: 132
##
## Scaled residuals:
##      Min       1Q   Median       3Q      Max
## -1.82573 -0.74889  0.00631  0.54684  1.95429
##
## Random effects:
##  Groups    Name        Variance Std.Dev.
##  record_id (Intercept) 0.2321   0.4818
##  Residual              0.1177   0.3431
## Number of obs: 108, groups:  record_id, 18
##
## Fixed effects:
##                             Estimate Std. Error        df t value Pr(>|t|)
```

```
## (Intercept)                1.860385   0.139403 31.863299  13.345 1.36e-14 ***
## drugMPH                    0.033377   0.114361 85.000001   0.292    0.771
## drugREB                    0.098094   0.114361 85.000001   0.858    0.393
## timepost-fatigue           0.008352   0.114361 85.000001   0.073    0.942
## drugMPH:timepost-fatigue -0.094013   0.161731 85.000001  -0.581    0.563
## drugREB:timepost-fatigue -0.170668   0.161731 85.000001  -1.055    0.294
## ---
## Signif. codes:  0 '***' 0.001 '**' 0.01 '*' 0.05 '.' 0.1 ' ' 1
##
## Correlation of Fixed Effects:
##             (Intr) drgMPH drgREB tmpst- dMPH:-
## drugMPH     -0.410
## drugREB     -0.410  0.500
## timepst-ftg -0.410  0.500  0.500
## drgMPH:tmp-  0.290 -0.707 -0.354 -0.707
## drgREB:tmp-  0.290 -0.354 -0.707 -0.707  0.500
```

```
summarize_lmer_effects(model_tmgdm_sqrt, inverse = function(x) x^2)
```

```
##                       Term Estimate   SE Estimate_inv CI_lower CI_upper p_value
## 1              (Intercept)     1.86 0.14         3.46     2.52     4.55   0.000
## 2                  drugMPH     0.03 0.11         0.00     0.04     0.07   0.771
## 3                  drugREB     0.10 0.11         0.01     0.02     0.10   0.393
## 4         timepost-fatigue     0.01 0.11         0.00     0.05     0.05   0.942
## 5 drugMPH:timepost-fatigue    -0.09 0.16         0.01     0.17     0.05   0.563
## 6 drugREB:timepost-fatigue    -0.17 0.16         0.03     0.24     0.02   0.294
```

```
anova(model_tmgdm, model_tmgdm_sqrt) # sqrt better , but dharma doesnt approve so we stick to lmm BETTE
```

```
## refitting model(s) with ML (instead of REML)
```

```
## Data: data_tmg_sub
## Models:
## model_tmgdm: dm ~ drug * time + (1 | record_id)
## model_tmgdm_sqrt: dm_sqrt ~ drug * time + (1 | record_id)
##                  npar    AIC    BIC   logLik deviance  Chisq Df Pr(>Chisq)
## model_tmgdm         8 415.46 436.92 -199.731   399.46
## model_tmgdm_sqrt    8 131.18 152.63  -57.589   115.18 284.28  0
```

```
sim_res2 <- simulateResiduals(model_tmgdm) #
# Plot diagnostics
plot(sim_res2)
```

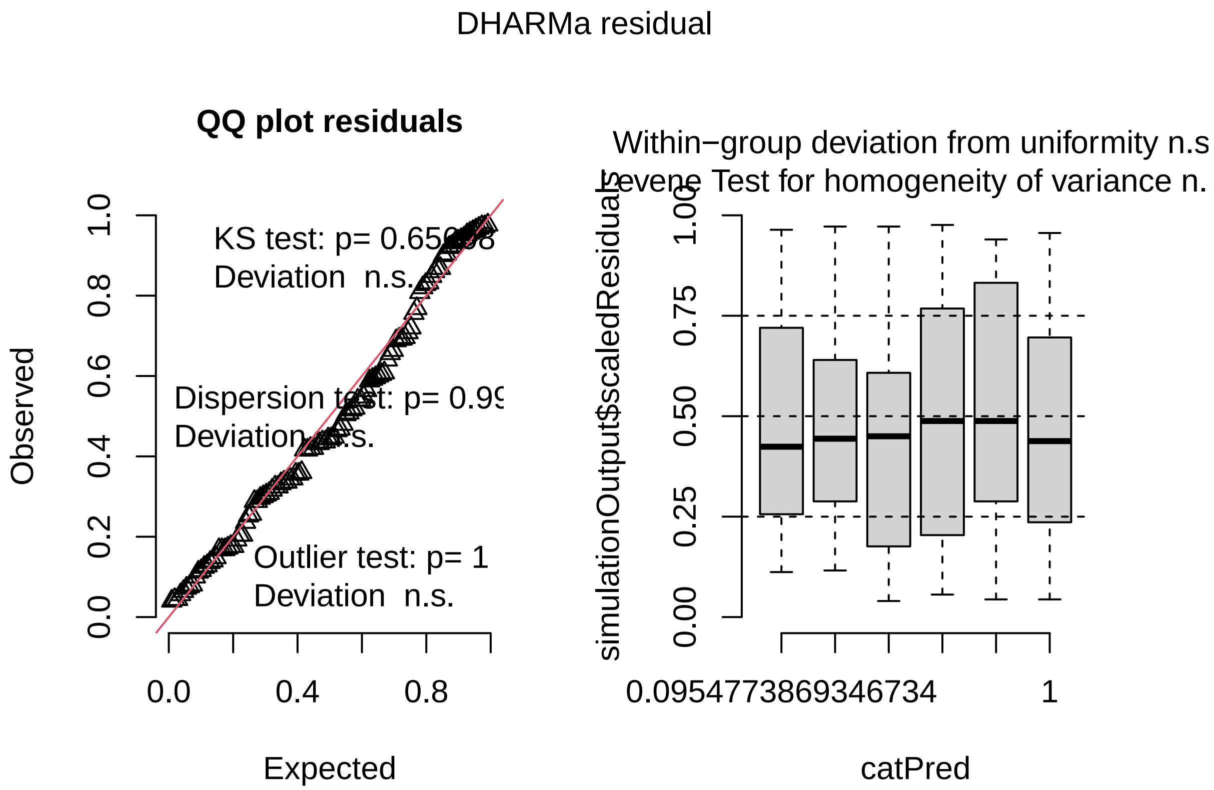


```r
sim_res2 <- simulateResiduals(model_tmgtc_sqrt) #
# Plot diagnostics
plot(sim_res2) # try with sqrt but dharma is not significant anymore
```

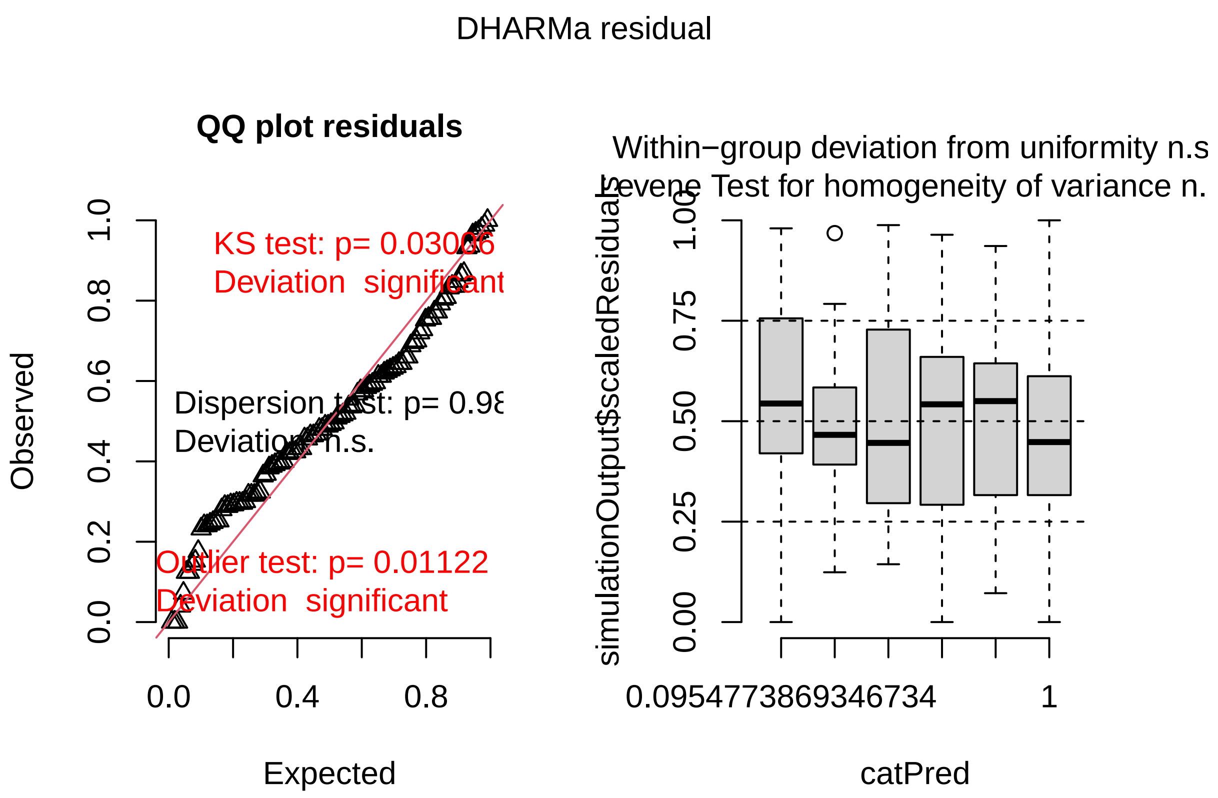


```r
anova(model_tmgdm, model_tmgdm_sqrt) # tmgdm is better so stick with lmm
```

```
## refitting model(s) with ML (instead of REML)
```

```
## Data: data_tmg_sub
## Models:
## model_tmgdm: dm ~ drug * time + (1 | record_id)
## model_tmgdm_sqrt: dm_sqrt ~ drug * time + (1 | record_id)
##                  npar    AIC    BIC   logLik deviance  Chisq Df Pr(>Chisq)
## model_tmgdm         8 415.46 436.92 -199.731   399.46
## model_tmgdm_sqrt    8 131.18 152.63  -57.589   115.18 284.28  0
```

NOTES AND CHECKS OF LAURISA

```r
# LEARNING EFFECT is large so we have to take this into consideration!
model_learn <- glmer(rep_nr ~ visit + (1 | record_id), data = data_perf, family = poisson())
summary(model_learn) # this model takes the record ID in consideration
```

```
## Generalized linear mixed model fit by maximum likelihood (Laplace
##   Approximation) [glmerMod]
##  Family: poisson  ( log )
## Formula: rep_nr ~ visit + (1 | record_id)
```

```
##    Data: data_perf
##
##      AIC      BIC   logLik deviance df.resid
##    781.0    789.0   -386.5    773.0       50
##
## Scaled residuals:
##     Min      1Q  Median      3Q     Max
## -5.7070 -1.4013 -0.3263  1.5108  5.6678
##
## Random effects:
##  Groups    Name        Variance Std.Dev.
##  record_id (Intercept) 0.2018   0.4492
## Number of obs: 54, groups:  record_id, 18
##
## Fixed effects:
##              Estimate Std. Error z value Pr(>|z|)
## (Intercept)   4.94193    0.10766  45.904  < 2e-16 ***
## visitvisit 2  0.09382    0.02603   3.604 0.000313 ***
## visitvisit 3  0.08701    0.02608   3.337 0.000847 ***
## ---
## Signif. codes:  0 '***' 0.001 '**' 0.01 '*' 0.05 '.' 0.1 ' ' 1
##
## Correlation of Fixed Effects:
##             (Intr) vstvs2
## visitvisit2 -0.127
## visitvisit3 -0.126  0.523
```

```
# people get less motivated over time. important consideration
#data_perf$motivation_logit <- log((data_perf$motivation + 0.01) / (100 - data_perf$motivation + 0.01))
#model_motivation_trans_interac <- lmer(log(motivation) ~ visit + (1 | record_id), data = data_perf)
#summary(model_motivation_trans_interac)

# old checks, elke suggested to use dharma instead
#plot(residuals(model_tmgdm)) #
#qqnorm(residuals(model_tmg_dm)) #
#qqline(residuals(model_tmg_dm)) # light tails!
#plot(fitted(model_tmg_dm), residuals(model_tmg_dm)) # light tails!
```
